# Selective labelling of GBA2 in cells with fluorescent β-d-arabinofuranosyl cyclitol aziridines†

**DOI:** 10.1039/d3sc06146a

**Published:** 2024-09-03

**Authors:** Qin Su, Max Louwerse, Rob F. Lammers, Elmer Maurits, Max Janssen, Rolf G. Boot, Valentina Borlandelli, Wendy A. Offen, Daniël Linzel, Sybrin P. Schröder, Gideon J. Davies, Herman S. Overkleeft, Marta Artola, Johannes M. F. G. Aerts

**Affiliations:** a Department of Medical Biochemistry, Leiden Institute of Chemistry, Leiden University P. O. Box 9502 2300 RA Leiden The Netherlands j.m.f.g.aerts@lic.leidenuniv.nl m.e.artola@lic.leidenuniv.nl; b Department of Bioorganic Synthesis, Leiden Institute of Chemistry, Leiden University P. O. Box 9502 2300 RA Leiden The Netherlands; c York Structural Biology Laboratory, Department of Chemistry, The University of York Heslington York YO10 5DD UK

## Abstract

GBA2, the non-lysosomal β-glucosylceramidase, is an enzyme involved in glucosylceramide metabolism. Pharmacological inhibition of GBA2 by *N*-alkyl iminosugars is well tolerated and benefits patients suffering from Sandhoff and Niemann–Pick type C diseases, and GBA2 inhibitors have been proposed as candidate-clinical drugs for the treatment of parkinsonism. With the ultimate goal to unravel the role of GBA2 in (patho)physiology, we sought to develop a GBA2-specific activity-based probe (ABP). A library of probes was tested for activity against GBA2 and the two other cellular retaining β-glucosidases, lysosomal GBA1 and cytosolic GBA3. We show that β-d-arabinofuranosyl cyclitol aziridine (β-d-Araf aziridine) reacts with the GBA2 active site nucleophile to form a covalent and irreversible bond. Fluorescent β-d-Araf aziridine probes potently and selectively label GBA2 both *in vitro* and *in cellulo*, allowing for visualization of the localization of overexpressed GBA2 using fluorescence microscopy. Co-staining with an antibody selective for the lysosomal β-glucosylceramidase GBA1, shows distinct subcellular localization of the two enzymes. We proffer our ABP technology for further delineating the role and functioning of GBA2 in disease and propose the β-d-Araf aziridine scaffold as a good starting point for the development of GBA2-specific inhibitors for clinical development.

## Abstract 2

Fluorescent β-d-arabinofuranosyl cyclitol aziridine activity-based probes potently and selectively label GBA2 both *in vitro* and *in cellulo*, allowing visualization of overexpressed GBA2 using fluorescent microscopy.

## Graphical abstract

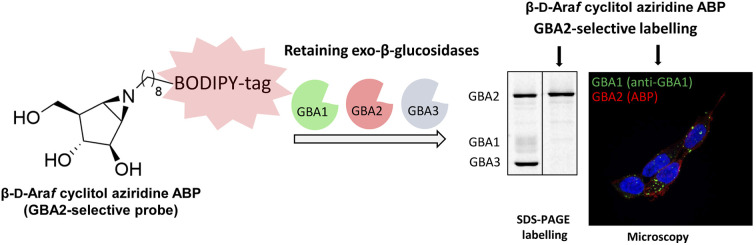

## Introduction

GBA2 (EC 3.2.1.45, CAZY^1^ GH116), a retaining β-glucosidase, was first discovered during the analysis of NBD-GlcCer metabolism in cultured cells. Ensuing studies demonstrated that GBA2, initially named non-lysosomal glucosylceramidase, is capable of hydrolysing glucosylceramide (GlcCer), which until that date was thought to be the exclusive activity of the enzyme deficient in Gaucher disease (GD), lysosomal glucocerebrosidase (GBA1, EC 3.2.1.45, GH30).^2^ GBA2 is now recognised to play a role in several inherited metabolic disorders.^3–6^ As well, companies have announced the development of GBA2 inhibitors for the treatment of parkinsonism.^7^ Despite this, the physiological role of GBA2, the consequences of cytosolic GlcCer metabolism and the interplay of GBA2 with lysosomal GlcCer breakdown is unclear.

GBA2 is a tightly membrane-bound enzyme whose activity can be assessed in cell and tissue lysates using the artificial fluorogenic substrate, 4-methylumbelliferyl-β-d-glucopyranoside (4MU-β-d-Glc). Compared to GBA1, GBA2 is less sensitive to inactivation by conduritol B epoxide (CBE), but more susceptible to inactivation by various detergents.^2^ The loss of enzymatic activity following its extraction from membranes complicates its purification, and the enzyme's identity was definitively elucidated only through the independent cloning of its cDNA.^8,9^ GBA2 homologues are found in several species, including archaea and bacteria,^10,11^ and GBA2 proteins degrading GlcCer are found, besides mammals, in plant and fish.^12–15^ To date mammalian GBA2 has defied resolution of a 3D structure, but structures of bacterial homologs (such as SSO1353 (GH116) in *S. solfataricus* and *Tx*GH116 in *T. xylanolyticum*^10,11,16^) provide insight in the catalytic machinery of the enzyme.

Human GBA2 is encoded by the GBA2 gene at locus *9p13.3* and is a 927 amino acid β-glucosidase with E527 as the catalytic nucleophile and D677 as the catalytic acid/base.^17^ It is a retaining glycosidase processing its substrate following a classical Koshland double displacement mechanism. GBA2 is initially synthesized as a soluble cytosolic protein that rapidly and tightly associates with membranes by an unknown mechanism. Various subcellular localizations of GBA2 have been reported in the literature. These include localization to the endoplasmic reticulum, Golgi apparatus, endosomes and the plasma membrane, as observed in studies where GBA2 was either overexpressed and visualized by western blotting (WB) or genetically tagged at the N-terminus or C-terminus with GFP.^9,18^ Notably, an investigation using cultured human melanoma cells and employing a subcellular fractionation technique combining density gradient centrifugation and free-flow electrophoresis revealed GBA2 activity in fractions coinciding with light endosomal structures.^19^ Furthermore, there is only one study using endogenous GBA2 (in cultured mouse embryonic neuronal cells) where the localization of GBA2 at the ER and Golgi apparatus was reported using a monoclonal antibody.^18^ The enzyme's localization may vary among cells, potentially reflecting their metabolic status. Unlike GBA1, GBA2 is able to hydrolyse both β-glucosidic and β-galactosidic substrates.^2^ GBA2 can also act as a transglycosidase, transferring glucose from GlcCer to, for instance, cholesterol, further adding to the mystery of the physiological role of GBA2.^20,21^

GBA2 is increasingly considered as therapeutic target for the treatment of a variety of diseases. Inhibition of GBA2 is a side effect of *N*-butyl-deoxynojirimycin (miglustat), a registered treatment for GBA1-deficient type 1 GD and Niemann–Pick disease type C (NPC) patients. While miglustat acts by pharmacological inhibition of glucosylceramide synthase (GCS),^22–24^ individuals under this treatment appear to develop no overt side effects upon long-term therapy. In line with this, inhibition of GBA2 activity with *N*-adamantanemethyloxypentyl-deoxynojirimycin (AMP-DNM) or its genetic ablation has been found to increase the life span of NPC mice.^23^ Tissues of NPC mice show partial increase in GBA2 and partial reduced GBA1 levels suggesting a compensatory mechanism between these enzymes.^15^ In type 1 GD mice generated by knockdown of GBA1 in hematopoietic stem cell lineage, GBA2 gene deletion was found to exert beneficial effects.^25^ In addition, increased GBA2 activities have been documented in leukocytes of GBA1-deficient GD patients.^26^ Finally and importantly, GBA2 knockout (KO) mice develop no overt pathology besides a partially reduced fertility, a phenomenon not observed in primates.^10,27^

Several classes of GBA2 inhibitors have been identified in the past decades. Competitive GBA2 inhibitors include iminosugars such as AMP-DNM with an IC_50_ value of approximately 1 nM for GBA2, and *N*-butyl-deoxynojirimycin (miglustat) with an IC_50_ value of 150–300 nM for GBA2.^19^ Mechanism-based, covalent and irreversible inhibitors, such as cyclophellitol aziridine act on GBA2 but also potently inhibit GBA1, and activity-based probes (ABPs) derived from these are not ideal for selective GBA2 detection and imaging in cells.^28^

**Fig. 1:**
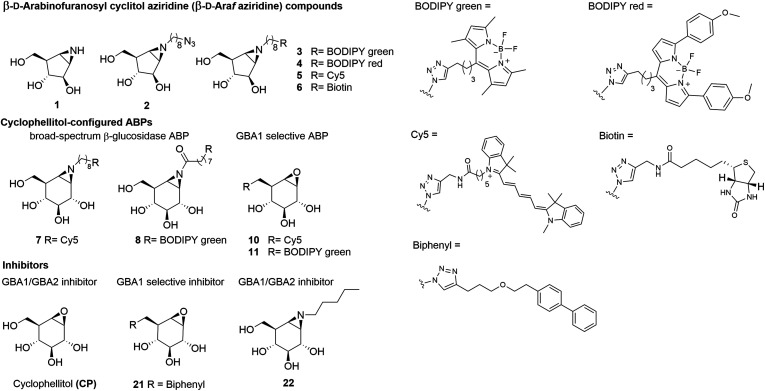
Chemical structures of β-d-arabinofuranosyl cyclitol aziridine 1–6, cyclophellitol epoxide and aziridine activity-based probes (ABPs) 7–11, and inhibitors 21 and 22.

For this reason, we sought to develop a GBA2-selective activity-based probe for *in cellulo* GBA2 imaging, and the results of studies in this direction are presented here.^29^ Previous studies showed the value of cyclophellitols as GBA1-specific probes,^15,30^ however the closely related cyclophellitol aziridine ABPs (ABP 7 and 8, Fig. 1) label both GBA1 and GBA2.^28,31^ Some cell types also express a soluble, cytosolic β-glucosidase with broad substrate specificity, termed GBA3 (EC 3.2.1.21, GH1), which also reacts with these ABPs.^28^ Since none of these ABPs react selectively with GBA2, we set out to investigate cyclophellitol-type compounds with varying configurations on their reactivity with GBA2 and related cellular retaining β-glucosidases. Our findings demonstrate that ABPs with a β-d-Araf aziridine configuration, published here for the first time (Fig. 1), potently and selectively label GBA2 by reacting with its catalytic nucleophile to form a covalent and irreversible bond.

## Results

### *In vitro* activity and selectivity of β-d-Araf cyclitol aziridine ABPs towards human β-glucosidases

**Fig. 2:**
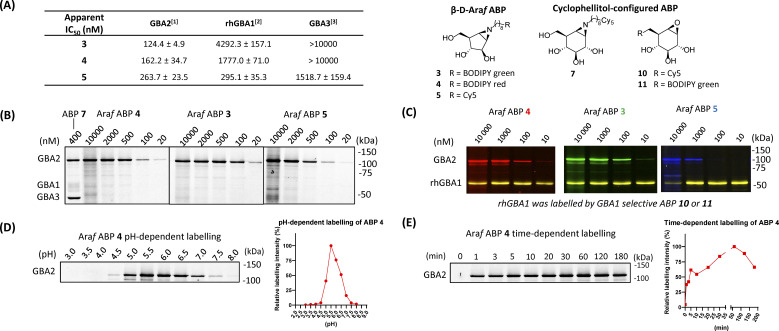
Fluorescent gel images of *in vitro* labelling of lysates using β-d-Araf aziridine ABPs. (A) Apparent IC_50_ values (nM) of β-d-Araf ABPs, determined in a 4MU fluorogenic substrate assay: [1] GBA2 = GBA1/GBA2 KO GBA2 OE HEK293T cell lysate, [2] rhGBA1 = imiglucerase (Cerezyme®). [3] GBA3 = GBA1/GBA2 KO GBA3 OE HEK293T cell lysate. Error ranges = ±SD, *n* = 3 replicates. (B) *In vitro* labelling after 30 min incubation at pH 6.0. HEK293T GBA1 endogenous and GBA2/3 OE cell lysate were used as enzyme source in these experiments. (C) β-d-Araf ABPs labelled mixture of HEK293T GBA1/2 KO, GBA2 OE lysate spiked with rhGBA1 (3 ng). Labelling was performed at pH 5.8. After incubation of β-d-Araf ABPs, rhGBA1 was labelled by ABP 10 at 500 nM (for 3 and 4) or ABP 11 at 500 nM (for 5). (D) *In vitro* pH-dependent labelling of ABP 4 (500 nM). Band quantification is shown on the right graph. (E) *In vitro* time-dependent labelling of ABP 4 (500 nM). Band quantification is shown on the right graph.

To identify an ABP that selectively labels human GBA2 over GBA1 and GBA3, a library of cyclophellitol-based ABPs with varying configurations (ESI Fig. S1†) was screened for their selectivity towards human retaining β-glucosidases. For this purpose, we initially assessed the inhibition properties of the compounds using recombinant human GBA1 (rhGBA1, imiglucerase), lysates of cells overexpressing GBA2 or GBA3 (both in combination with knockout of the other retaining β-glucosidases). In a preliminary screen, enzymes were pre-incubated for 30 min with the tested compounds, followed by the addition of the fluorogenic substrate, 4MU-β-d-Glc, and subsequent quantification of released fluorescent 4MU after 30 minutes. This screening brought our attention to a new set of β-d-Araf cyclitol aziridines as potential GBA2 inhibitors. The synthesis of these compounds is detailed in the ESI.† Pre-incubation of the enzymes with β-d-Araf cyclitol aziridine 1 did not show inhibitory effect towards any of the β-glucosidases assayed up to 50 μM, whereas *N*-azido-octyl aziridine 2 displayed inhibition of all three β-glucosidases (apparent IC_50_ values: GBA2 630 nM, GBA1 2730 nM, GBA3 8150 nM) with some selectivity for GBA2 over GBA1 and GBA3 (ESI Table S1†). Interestingly, BODIPY green- and BODIPY red-tagged ABPs 3 and 4 exhibited substantial activity towards GBA2 (apparent IC_50_ value: 120–160 nM) with clear selectivity (defined as IC_50_ enzyme 1/IC_50_ enzyme 2) for GBA2 over GBA1 and GBA3 (Fig. 2A). In contrast, β-d-Araf ABP 5 equipped with a Cy5 fluorophore inhibited GBA1 and GBA2 with about equal potency (apparent IC_50_ values of 250–300 nM). On the other hand, the biotin-tagged β-d-Araf compound 6 is a poor inhibitor of all three glucosidases (apparent IC_50_ > 8 μM). Arabinofuranosyl cyclitol 12–20 with various configurations (α-l-Araf and β-l-Araf) were also evaluated (ESI Fig. S1†). Most of these proved to be poor GBA2 inhibitors (apparent IC_50_ values > 20 μM), and none matched the selectivity of β-d-Araf cyclitol aziridines 3 and 4 for GBA2 (Table S1†).

Armed with ABPs 3 and 4, both of which displayed high selectivity for GBA2 over GBA1 and GBA3, we next sought to analyse their activity in cell lysates. To this end, lysates of HEK293T cells containing all β-glucosidases (endogenous GBA1 with overexpressed GBA2 and GBA3) were treated with ABPs 3, 4, 5 or 7, followed by protein separation by SDS-PAGE and fluorescence scanning of the wet gel slabs. As illustrated in Fig. 2B, ABP 7 labelled all three β-glucosidases, consistent with the previous report.^31^ In contrast, the β-d-Araf aziridine ABPs 3 and 4 selectively labelled GBA2 at a concentration of 100 nM, while Cy5 tagged ABP 5 did so at 500 nM. The GBA2 selectivity of β-d-Araf cyclitol aziridines 3, 4 and 5 was confirmed in a mixture of a lysate of overexpressed GBA2 cells spiked with 3 ng rhGBA1 (Fig. 2C and ESI Fig. S3†). In this experiment, the enzyme mixture was first incubated with β-d-Araf aziridine ABPs 3, 4, or 5 for 30 minutes, after which selective GBA1 ABP 10 ^32^ or 11 ^30^ was added to label the remaining active rhGBA1 active site. This experiment revealed the ability of β-d-Araf cyclitol aziridine ABPs, and in particular 3 and 4, to selectively label GBA2 without significant rhGBA1 labelling (marginal GBA1 labelling occurring at 10 μM). The pH and incubation time dependence labelling of GBA2 in HEK293T cells containing all three retaining β-glucosidases by β-d-Araf ABPs was next investigated (Fig. 2D, E and ESI Fig. S4†). All ABPs selectively label GBA2 at a pH range of 4.5–7.5. Using 500 nM of ABP 4, GBA2 labelling occurred within a minute, and maximal labelling was observed after 20–30 minutes. Labelling kinetic studies in HEK293T GBA1/2 KO, GBA2 OE lysate showed that ABP 4 irreversibly inhibited GBA2 within one minute. Therefore, only a combined inactivation rate constant (*k*_inact_) and binding constant (*K*_I_) ratio (*k*_inact_/*K*_I_) could be measured. ABP 4 exhibited rapid first-order labelling kinetics with a *k*_inact_/*K*_I_ of 1.15 ± 0.565 min^−1^ μM^−1^, demonstrating similar fast kinetics as reported for GBA1-targeting cyclophellitol aziridine-based probes (ESI Fig. S4-1† for SDS-PAGE gels and labelling kinetics).^33^ Importantly, no labelling of GBA1 or GBA3 was detected under varying pH and time conditions (ESI Fig. S4-2†).

### Identification of the catalytic nucleophile of GBA2 reacted with β-d-Araf cyclitol aziridines

**Fig. 3:**
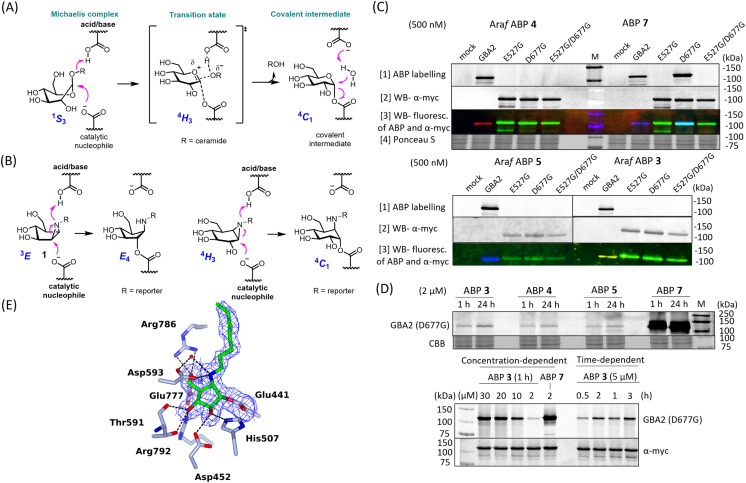
Mechanism of β-d-Araf aziridine inhibition of GBA2. (A) Conformational itinerary of the Koshland double-displacement mechanism employed by retaining β-d-glucosidases from the Michaelis complex to the covalent intermediate. (B) β-d-Arabinofuranosyl cyclitol aziridine 1 inhibits retaining β-d-glucosidases by adopting an envelope-like ^3^E conformation prior to reacting with the catalytic amino acid similar to the ^1^S_3_ Michaelis complex conformation, whereas cyclophellitol aziridines mimic the ^4^H_3_ transition state conformation. (C) Fluorescent gel images of 500 nM β-d-Araf aziridine ABPs (3–5) and cyclophellitol-aziridine ABP 7 labelling HEK293T lysate with overexpressed GBA2 E527G, D677G or E527G/D677G mutants containing a myc tag. mock = HEK293T GBA1/GBA2 KO lysate, GBA2 = HEK293T GBA1/GBA2 KO GBA2 OE cell lysate. Fluorescent gel images were captured by: row [1] = fluorescence scanning of ABP labelling in slab-gel, row [2] = fluorescence scanning of anti-myc antibody (α-myc) using western blotting to examine the expression of GBA2 mutant, row [3] = western blot of ABP fluorescence and α-myc overlap, row [4] = Ponceau S staining to show protein loading for sets of ABP 4 and ABP 7. (D) Fluorescent gel images of concentration-dependent and time-dependent labelling of β-d-Araf aziridine ABPs of D677G mutant GBA2. Upper image: 2 μM β-d-Araf ABPs 3–5 and ABP 7 incubated with GBA2 D677G mutant for 1 h (at 37 °C) or 24 h (at 4 °C). Lower image: β-d-Araf ABP 3 labelling of GBA2 D677G mutant at 37 °C at increasing concentrations and incubation times. (E) Structure of *Tx*GH116 complexed with 2 showing electron density difference map calculated for the ligand and side chain of Glu441, contoured at 2.5*σ* (0.275 electrons per Å^3^), and showing hydrogen bonds represented as dashed lines.

GBA2, like *Tx*GH116 (the bacterial homologue of GBA2), induces hydrolysis through the conventional Koshland two-step double-displacement conformational pathway typical of retaining β-glucosidases, progressing from ^1^S_3_ to ^4^H_3_ to ultimately adopt the ^4^C_1_ in the covalent complex (Fig. 3A).^11^ In contrast to cyclophellitol aziridines, which mimic the ^4^H_3_ transition state, β-d-Araf aziridine adopts an ^3^E conformation^34^ which resembles the ^1^S_3_ initial Michaelis complex conformation (Fig. 3B).^35^ Previous research had established that E527 (catalytic nucleophile) and D677 (catalytic acid/base) are the catalytic residues in the human GBA2 active site.^17^ To investigate whether β-d-Araf aziridine ABPs bind GBA2 in an activity-based manner, first mutants of GBA2 were generated by substituting either the E527 nucleophile or the D677 acid/base. Lysates from cells expressing these mutant GBA2 proteins were then incubated with β-d-Araf ABPs 3–5 or cyclophellitol ABP 7 and their labelling pattern was analysed (Fig. 3C). None of the ABPs were found to label the E527G mutant or the E527G/D677G double mutant, demonstrating that these probes require the nucleophile E527 for reaction with GBA2. Notably, β-d-Araf ABPs 3–5 exhibited poor reactivity with the acid/base mutant D677G when compared to ABP 7 (Fig. 3D). At higher ABP concentrations and/or longer incubation times higher degree of labelling of the acid/base mutant GBA2 was detected also with ABP 3.

To firmly establish the mode of action of β-d-Araf cyclitol aziridines as mechanism-based GBA2 inhibitors, the 3-D structure of the GH116 bacterial GBA2 homolog, *Tx*GH116 from *Thermoanaerobacterium xylanolyticum* in complex with β-d-Araf compound 2 was solved at 1.9 Å resolution. The −1 subsite of *Tx*GH116 is well conserved relative to human GBA2. Electron density shows unambiguous covalent reaction of 2 with the catalytic nucleophile of *Tx*GH116 (Fig. 3E). Reacted compound 2 is anchored by several hydrogen bonds. The OH group on C2 interacts with NE2 His507 and OD2 Asp452, and the OH on C3 with OD2 Asp452, NH2 Arg792 and OG1 and the alcohol of Thr591. The OH on C5 is hydrogen-bonded to OE2 Glu777 and to NH1 and NH2 Arg786. In addition, the amine from the ring-opened aziridine group forms a hydrogen bond to OD2 Asp593 and to a water molecule (which also interacts with OD1 Asp593). There is insufficient electron density to allow modelling of the end of the octyl chain and the azido group, which extend into a more open region at the edge of the protein where they are less constrained.

When the structure of *Tx*GH116 with cyclophellitol aziridine (8R06.pdb) is superposed on our structure with 2 (ESI Fig. S13†), the ligand ring C atoms lie in similar positions, apart from C1 and C2 of the former, which straddle the position of the covalently bound C1 atom of 2. There are very similar hydrogen bonding interactions with the active site residues to those for 2. However, both the hydroxyl groups on C2 and C3 of the cyclophellitol-configured aziridine form hydrogen bonds to His507, whereas for 2, only a single hydrogen bond is possible with the side chain. The hydroxyl group on C2 of the former also forms a hydrogen bond to the carboxyl oxygen of Glu441 which does not participate in the covalent link to the ligand. Having demonstrated specific active site nucleophile labelling, and GBA2 selectivity, we felt confident to next investigate the use of the β-d-Araf ABPs for studying GBA2 orthologs across species.

### *In vitro* labelling across species labelling of β-d-Araf cyclitol aziridine ABPs

**Fig. 4:**
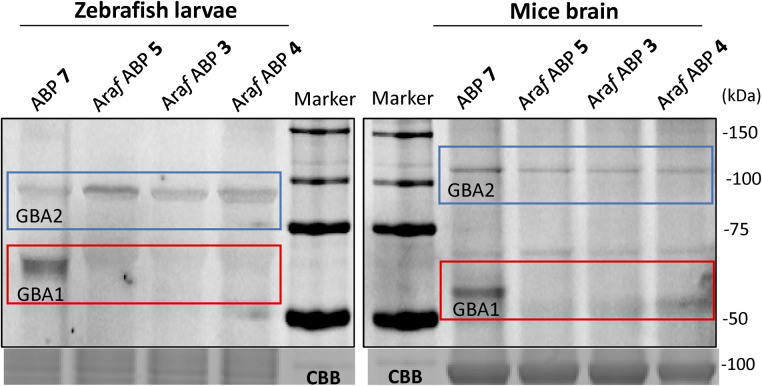
Fluorescent gel images of selective labelling of GBA2 orthologues by β-d-Araf aziridine ABPs in different species. Cyclophellitol-aziridine ABP 7 (1 μM) and β-d-Araf ABPs (ABP 3 at 1 μM and ABP 4 and 5 at 2.5 μM) were incubated with homogenates of zebrafish (15 μg total protein) and mice brain (15 μg total protein) for 1 h at 37 °C.

GBA2 orthologs are highly conserved among different species. BLAST analysis revealed that human GBA2 shares 87% sequence identity and 93% similarity in the catalytic domain with murine GBA2 and 66% identity and 79% similarity with the zebrafish (*Danio rerio*) enzyme.^14^ The labelling capability of β-d-Araf aziridine ABPs toward GBA2 orthologs in these species was therefore evaluated. Homogenates of zebrafish larvae or mice brain homogenates were incubated with β-d-Araf ABPs 3–5 or broad-spectrum ABP 7 (Fig. 4). As observed in human cell extracts, β-d-Araf ABPs 3–5 also selectively label GBA2 in these species, whereas ABP 7 show cross-reactivity towards GBA1.

**Fig. 5:**
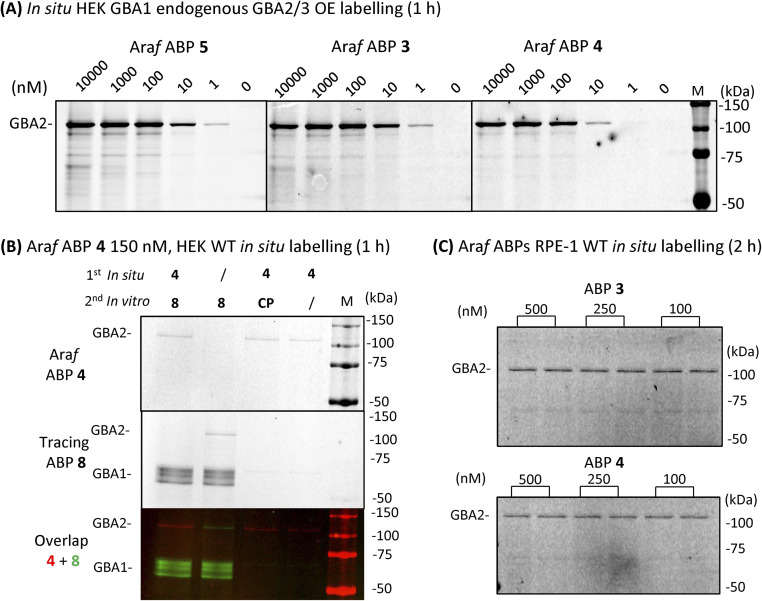
Fluorescent gel images of β-d-Araf ABPs labelled GBA2 in intact cells. (A) *In situ* cell labelling of endogenous GBA1 and overexpressed GBA2/3 in intact cells by β-d-Araf ABPs at varying concentrations and 1 h incubation. (B) Wild-type HEK293T cells treated *in situ* with 150 nM ABP 4 for 1 h, followed by incubation with competitor 8 or CP *in vitro* during the lysis procedure; slash = DMSO replacement, CP = cyclophellitol (1 μM), ABP 8 (1 μM). (C) ABP 3 and 4*in situ* labelled GBA2 in wild-type RPE-1 intact cells after 2 h incubation.

We next sought to investigate whether we could identify and localize active GBA2 molecules in human cells using our β-d-Araf aziridine ABPs. For this purpose, HEK293T cells with endogenous GBA1 and overexpressed GBA2/GBA3 were treated with varying concentrations of β-d-Araf aziridine ABPs 3–5 for 1 h, after which the cells were harvested and washed multiple times prior to lysis. The lysates were then denatured, their protein content separated by SDS-PAGE and the resulting wet gel slabs scanned for fluorescence. Following this procedure, we observed that all three β-d-Araf ABPs 3–5 enter intact cells, where they react with GBA2, and given the enzyme lifetime (>24 h), also with newly synthesized GBA2 (Fig. 5A, ESI Fig. S8A and B†). BODIPY tagged ABPs 3 and 4 proved to be the most effective GBA2 probes in these experiments and inactivate GBA2 almost completely at 100 nM final concentration (ESI Fig. S8B†).

To address the concern that our ABPs may attach to the cell surface and subsequently label GBA2 *in vitro* following cell lysis, a non-tagged GBA2 inhibitor was added to the lysis buffer. The presence of high concentrations of cyclophellitol (CP) or cyclophellitol aziridine ABP 8, both potent human GBA1 and GBA2 inactivators, did not diminish GBA2 labelling efficiency by ABP 4 (Fig. 5B), thus indicating that these ABPs indeed labelled GBA2 *in situ*.

Importantly, the GBA2 selectivity of β-d-Araf ABPs 3–5 was maintained during *in situ* labelling of wild-type HEK293T cells (ESI Fig. S9†). GBA1 labelling only occurred when using a high concentration (500 nM) and longer incubation time (2.5 h) for ABP 5, while ABP 3 and 4 did not visibly label GBA1 under these conditions (Fig. 5B and ESI Fig. S9†). Even after 24 h incubation, similar results were obtained: ABP 4 selectively labelled GBA2 at 10 nM, with only slight concomitant GBA1 labelling at concentrations higher than 100 nM (ESI Fig. S10†). Selective GBA2 labelling by ABP 3 and 4 finally was also observed in human retinal pigment epithelial-1 cells (Fig. 5C).

### Localization of GBA2 with an β-d-Araf cyclitol aziridine ABP

**Fig. 6:**
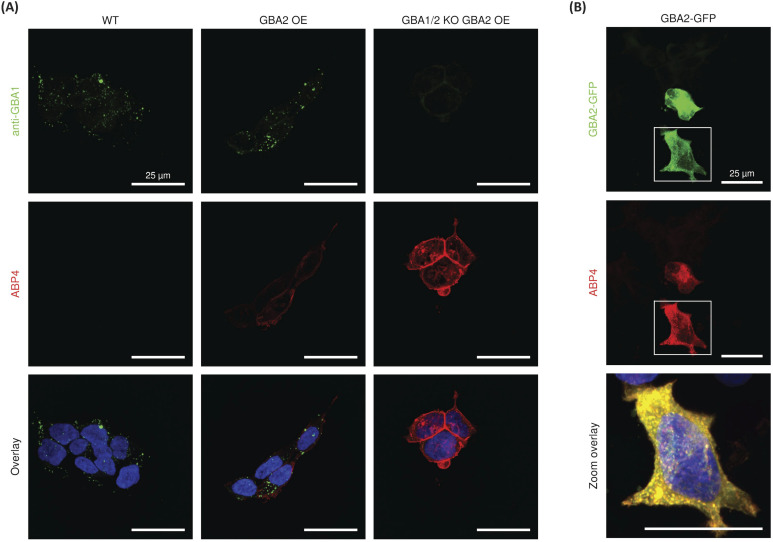
Confocal fluorescence microscopy of (A) HEK293T wild-type (WT), GBA2 overexpressed (OE), and GBA1/2 knock out (KO) + GBA2 overexpressed (OE) cells labelled with β-d-Araf aziridine BODIPY-red ABP 4 (50 nM) for 2 h, and after fixation with an α-GBA1 antibody (green). (B) Labelling of HEK293T cells transiently overexpressing mouse GBA2 with a C-terminal GFP-tag (green) with BODIPY-red ABP 4 (50 nM) for 2 h. In all samples nuclei were stained with 10 μg mL^−1^ DAPI (blue).

For the final set of experiments, red fluorescent ABP 4 was used to study the localization of GBA2 in HEK293T cells. After incubation with ABP 4 at 50 nM for 2 h, the samples were fixed and also co-stained with a green-fluorescent anti-GBA1-antibody in order to discern the difference in localization between GBA1 and GBA2. Confocal microscopy of wild-type (WT) HEK293T cells showed an unambiguous staining for GBA1 with a distinct perinuclear lysosomal distribution pattern. However, no clear signals for ABP 4 modified proteins were observed (Fig. 6). When using wild-type RAW 264.7 cells, which show slightly more *in vitro* labelled GBA2 than wild-type HEK293T, it was neither possible to visualize endogenous GBA2 (ESI Fig. 11B†). Attention was therefore redirected to the use of GBA2 overexpression (OE) cells and GBA1/2 knockout (KO) + GBA2 OE cells.^20,31^ These cells did present clear GBA2 labelling, localized primarily to the cell membrane (Fig. 6). It is apparent that GBA1 and GBA2 have different sub-cellular localization, since no overlap of their labelling is seen.

To confirm the specific staining of GBA2, cells overexpressing GBA2 were pre-treated with either the specific GBA1 inhibitor 21 ^32^ or the GBA1/GBA2 inhibitor 22.^36^ Confocal microscopy showed no change in the staining of GBA2 after pre-treatment with the GBA1 specific inhibitor 21, whereas the signal of GBA2 was completely abrogated by pre-treatment with the dual GBA1/GBA2 inhibitor 22 (Fig. S11A†). In addition, labelling of GBA2 with ABP 4 (50 nM) in HEK293T GBA2 KO cells transiently overexpressing mouse GBA2 C-terminally tagged with GFP showed highly overlay of the GFP and BODIPY-red signals (Fig. 6B). In all, this demonstrates that ABP 4 is able to specifically label overexpressed GBA2 *in situ*.

## Discussion

GBA2 attracts increasing attention given its potential role in pathophysiological mechanisms in a variety of human diseases. While the catalytic machinery, the mode of action and the substrate specificity of the enzyme has now been firmly established, little is known about its physiological role and its subcellular localization is a matter of debate. The acquisition of such knowledge is hampered by the absence of cell-permeable, GBA2-selective chemical probes, comparable to the counterpart developed for the selective visualization of active GBA1 in living cells.^30,37–39^ The work described here was aimed to rectify this situation. Screening of our activity-based glycosidase probes library led to the discovery of fluorescent β-d-Araf cyclitol aziridines that selectively label GBA2 both *in vitro* and in intact cells. The labelling occurs through covalent mechanism-based binding of the ABP and is abolished by mutagenesis of the catalytic nucleophile. Fluorescent microscopy of GBA2-labelled cells clearly demonstrates that, unlike GBA1, GBA2 is not located within lysosomes.

While labelled GBA2 is easily detected in cells overexpressing the enzyme, the intensity of the fluorescent signal in wild type HEK293T cells (Fig. 6A) and RAW264.7 cells (ESI Fig. 11B†), is relatively weak. Future use of more advanced fluorescence microscopy (for instance, including spectral imaging and/or use of a supersensitive camera with higher quantum efficiency) may improve detection of endogenous GBA2. Also, functionalization of the β-d-Araf cyclitol aziridine scaffold with fluorophores exhibiting higher quantum yields and photostability may aid in the detection of endogenous GBA2.^40^ It should be kept in mind that disperse distribution of GBA2 among membranes, contrary to the intrinsic concentration of GBA1 molecules in lysosomes, does not favour detection by simple fluorescence microscopy.

The observed high affinity and selectivity of β-d-Araf cyclitol aziridines equipped with a hydrophobic fluorescent tag for labelling GBA2 is quite remarkable. Very recent publication by Shimokawa and coworkers^41^ reports that a GH116 *exo*-β-d-arabino-furanosidase from *Microbacterium arabinogalactanolyticum* termed ExoMA2 shows similarities in structure to that of the GH116 β-glucosidase from *Thermoanaerobacterium xylanolyticum* (*Tx*GH116). Both enzymes have a two-domain structure consisting of N-terminal β-sandwich and C-terminal (α/α) 6-barrel domains, the latter being the catalytic domain. The two catalytic residues, and several residues in the pocket are conserved, but substrate recognition at subsite −1 differs. Given the similarities in the catalytic pocket, the catalytic residues and even the transglycosylation abilities^20,41,42^ of the three enzymes, it is perhaps not surprising that β-d-Araf cyclitol aziridines bind well to the GBA2 active site.

## Conclusions

In conclusion, screening of our ABP library towards β-glucosidase GBA1, GBA2 and GBA3 has led to the identification of β-d-Araf aziridine ABPs as a new class of covalent GBA2 selective ABPs. These probes address the need for selective GBA2 ABP tools, allowing for specifically monitoring of GBA2 and serving as specific GBA2 suicide inhibitors to selectively inactivate the enzyme. The β-d-arabinofuranosyl cyclitol aziridine ABPs represent novel tools for studying the intriguing enzyme GBA2, and future efforts to develop more GBA2-selective inhibitors based on the functionalized β-d-Araf scaffold is warranted.

## Data availability

The authors declare that all data supporting the findings of this study are available within the article and ESI, and raw data files are available from the corresponding author upon request.†

## Author contributions

J. M. F. G. A. and M. A. conceived and designed the investigations, interpreted experimental data, wrote and proofread the manuscript. Q. S. conducted most of chemical biological experiments, interpreted experimental data, wrote and proofread the manuscript. M. L. conducted microscopy experiment, R. F. L., E. M., S. P. S., D. L. and V. B. synthesized β-d-Araf-, β-l-Araf- and α-l-Araf-based probes, M. J. conducted chemical biological assays, R. G. B. assisted in the interpretation of experimental data, W. A. O. solved the 3-D structure of the enzyme–ligand complex, and together with G. J. D. and H. S. O wrote and proofread the manuscript.

## Conflicts of interest

There are no conflicts to declare.

## Supplementary Material

SC-OLF-D3SC06146A-s001
