# Supplementary material for: Selective labelling of GBA2 in cells with fluorescent β-d-arabinofuranosyl cyclitol aziridines
Source: Chem Sci. 2024 Sep 3;15(37):15212–20. doi: 10.1039/d3sc06146a (PMC11375437; doi:10.1039/d3sc06146a)

Supplementary Information (SI) for Chemical Science.
This journal is © The Royal Society of Chemistry 2024

Supporting Information

Selective labelling of GBA2 in cells with fluorescent β-D-arabinofuranosyl cyclitol aziridines

Qin Su,[a] Max Louwerse,[a] Rob F. Lammers,[a] Elmer Maurits,[b] Max Janssen,[a] Rolf G. Boot,[a] Valentina Borlandelli,[b] Wendy A. Offen,[c] Daniël Linzel,[b] Sybrin Schröder,[b] Gideon J. Davies,[c] Herman S. Overkleeft,[b] Marta Artola,[*a] Johannes M. F. G. Aerts[*a]

a. Department of Medical Biochemistry, Leiden Institute of Chemistry, Leiden University, P. O. Box 9502, 2300 RA Leiden, The Netherlands.

b. Department of Bioorganic Synthesis, Leiden Institute of Chemistry, Leiden University, P. O. Box 9502, 2300 RA Leiden, The Netherlands.

c. York Structural Biology Laboratory, Department of Chemistry, The University of York, Heslington, York, YO10 5DD (UK)

* Corresponding author.

Table of Contents

1 Supporting tables and figures 3
2 Biochemistry 16
2.1 Materials 16
2.2 Cell culture 16
2.3 Generation of cells genetically modified for β-glucosidase expression 16
2.4 Cell lysis 16
2.5 4MU fluorogenic substrate assay for apparent IC_{50} determination 16
2.6 Visualization of ABP labelling towards β-glucosidases by activity-based protein profiling (ABPP) with SDS-PAGE 17
2.7 Determination of kinetic parameters of ABP **4** 17
2.8 β-Glucosidase inactivation visualized by competitive ABPP with SDS-PAGE 18
2.9 Irreversibility evaluation by competition with irreversible ABP 18
2.10 Stability of β-D-Ara*f* aziridine binding towards GBA2 18
2.11 Reactivity of β-D-Ara*f* aziridines towards GBA2 mutants 18
2.12 Reactivity of β-D-Ara*f* aziridines towards GBA2 orthologue in other species 19
2.13 *In situ* intact cells labelling and inhibition assays 19
2.14 *In situ* visualization of GBA2 for confocal microscopy 19
2.15 Expression, purification and crystallization of *Tx*GH116 20
2.16 Data collection and refinement 20
3 Chemistry 21
3.1 General 21
3.2 Synthesis of β-D-arabinofuranosyl cyclitol aziridine ABPs 22
3.3 Experimental procedures and characterization data 22
3.4 NMR data 27
4 Abbreviations 36
5 References 37
Appendix - raw gels 40

1 Supporting tables and figures

Table S1. Apparent IC_{50} values (nM) of arabinofuranosyl cyclitol analogues toward β-glucosidases.

**β-D-Ara*f***	GBA2 [a]	rhGBA1 [b]	GBA3 [c]
**1** (aziridine)	> 50000	> 50000	> 50000
**2** (octyl-azido)	630.0 ± 95.6	2730.0 ± 907.2	8149.6 ± 1425.8
**3** (BODIPY green)	124.4 ± 4.9	4292.3 ± 157.1	>10000
**4** (BODIPY red)	162.2 ± 34.7	1777.0 ± 71.0	> 10000
**5** (Cy5)	263.7 ± 23.5	295.1 ± 35.3	1518.7 ± 159.4
**6** (Biotin)	8481.0 ± 762.6	10671.0 ± 786.5	10281.7 ± 1896.5
**α-L-Ara*f***	GBA2 [a]	rhGBA1 [b]	GBA3 [c]
**12** (epoxide)	> 50000	> 50000	> 50000
**13** (aziridine)	> 50000	> 50000	> 50000
**14** (aziridine Cy5)	1850 (30 min)<br>N.D. (3 h)	> 50000 (30 min)<br>1340 (3 h)	> 50000 (3 h)
**β-L-Ara*f***	GBA2 [a]	rhGBA1 [b]	GBA3 [c]
**15** (epoxide)	> 20000	> 20000	> 20000
**16** (epoxide Cy5)	> 20000	> 20000	> 20000
**17** (epoxide BODIPY green)	> 20000	> 20000	> 20000
**18** (aziridine)	> 20000	> 20000	> 20000
**19** (aziridine Cy5)	> 20000	> 20000	> 20000
**20** (aziridine BODIPY green)	20000	> 20000	> 20000

N.D. = not determined. Enzymes: [a] GBA2 = GBA1/GBA2 KO and GBA2 OE HEK293T cell lysate, [b] rhGBA1 = Imiglucerase (Cerezyme®), [c] GBA3 = GBA1/GBA2 KO and GBA3 OE HEK293T cell lysate. Error ranges = ± SD, n = 3 replicates for β-D-Ara*f* aziridine compounds, n = 1 replicates for α-L-Ara*f* and β-L-Ara*f* compounds.

Table S2. Data collection and refinement statistics for the complex of *Tx*GH116 and β-D-Ara*f* **2**

	***Tx*GH116-2**
**Data collection**	
Space group	P 21 21 21
Cell dimensions	
a, *b*, *c* (Å)	55.0, 165.1, 178.9
α, β, γ (°)	90.0, 90.0, 90.0
Resolution (Å)	56.09 – 1.90 (1.93 - 1.90)*
Total no. reflections	1381158
No. unique reflections	129333 (6302)
R_{sym} or R_{merge}	0.249 (2.052)
R_{pim}	0.079 (0.643)
$CC_{1/2}$	0.996 (0.554)
$I / \sigma I$	7.1 (1.3)
Completeness (%)	100.0 (100.0)
Redundancy	10.7 (10.9)
**Refinement**	
No. reflections working set	122902
No. reflections test set	6306
R_{work} / R_{free}	0.18/0.23
No. atoms	
Protein	12592
Ligand/ion	53
Water	880
B-factors	
Protein	24.9
Ligand/ion	31.9
Water	29.9
R.m.s deviations	
Bond lengths (Å)	0.006
Bond angles (°)	1.513
Ramachandran plot residues	
In most favorable regions (%)	95.1
In allowed regions (%)	4.4
PDB code	8R1M

* Figures for highest resolution shell given in parentheses

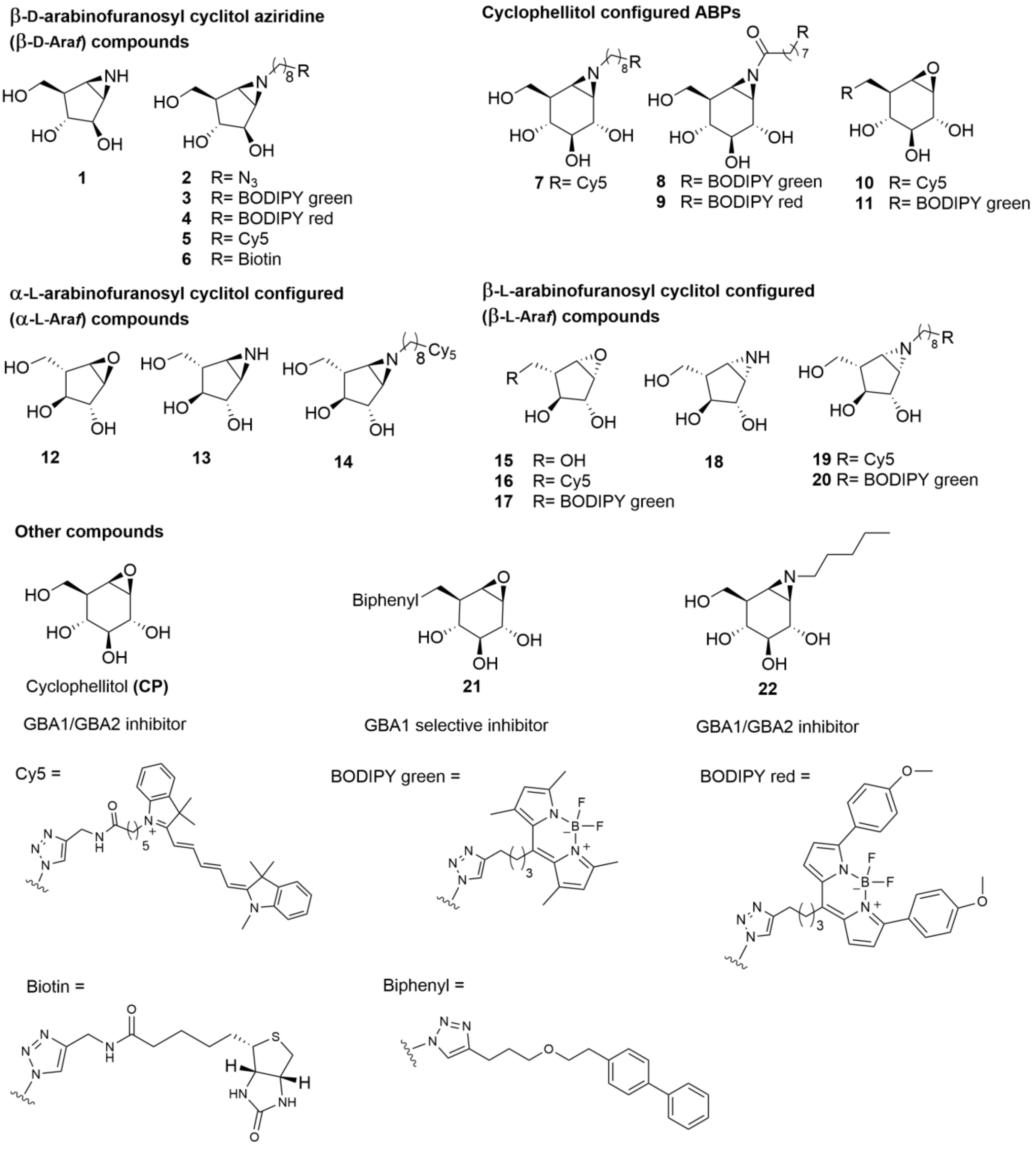

Figure S1. Compound library used in this study.

Apparent IC$_{50}$ curves of β-D-Ara*f* compounds

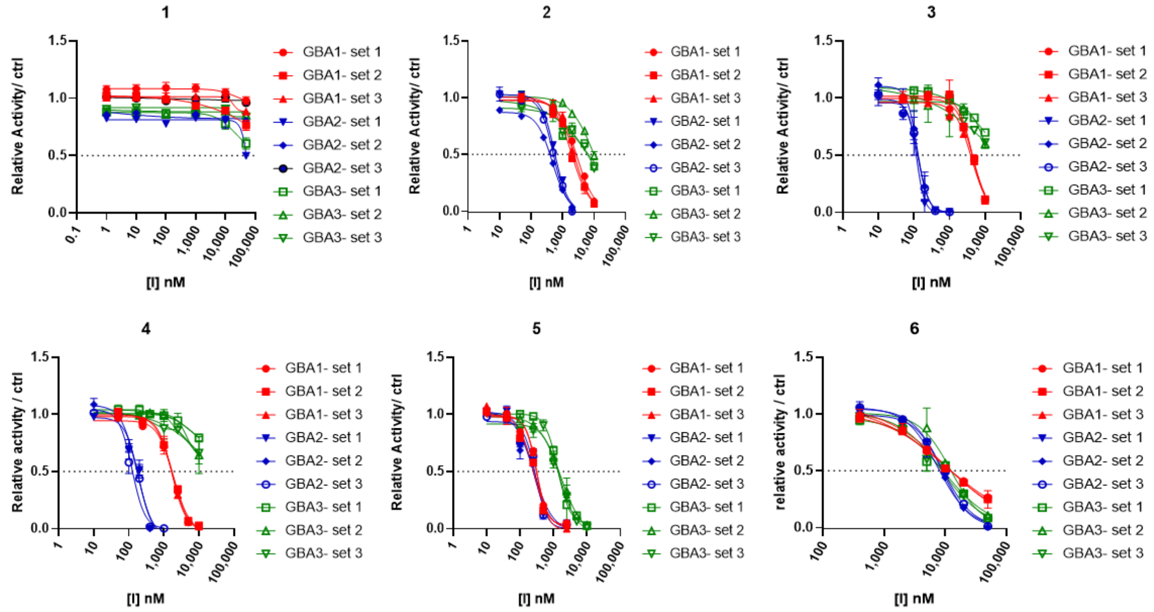

Apparent IC$_{50}$ curves of α-L-Ara*f* compounds

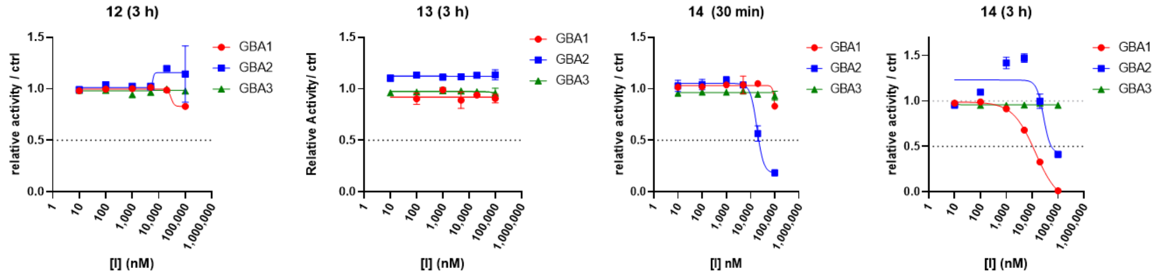

Apparent IC$_{50}$ curves of β-L-Ara*f* compoumds

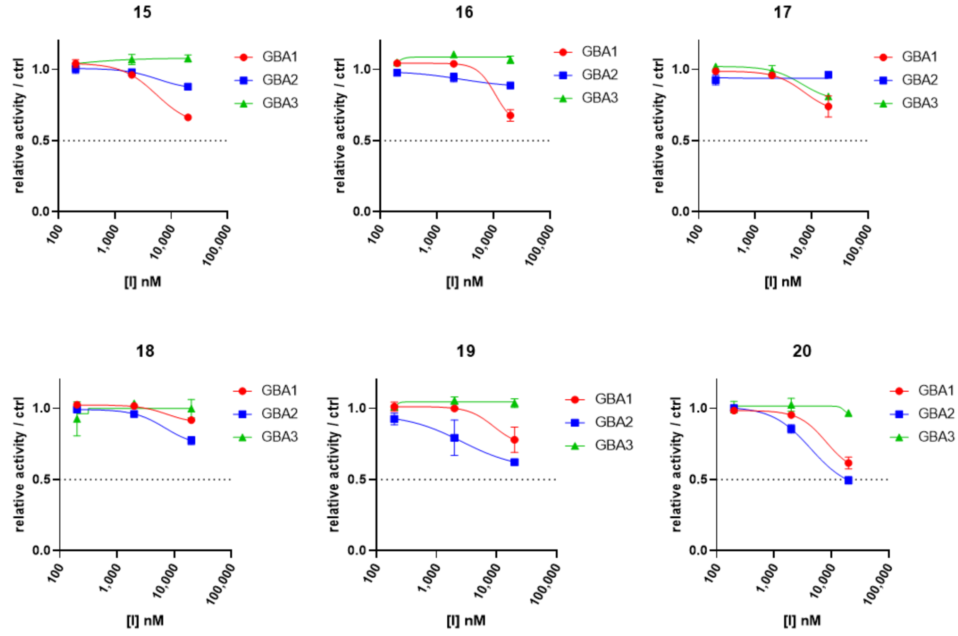

Figure S2. Apparent IC$_{50}$ curves of arabinofuranosyl configured compounds **1**-**6** and **12**-**20** using 4MU-β-D-Glc fluorogenic substrate for β-glucosidase activity assays.

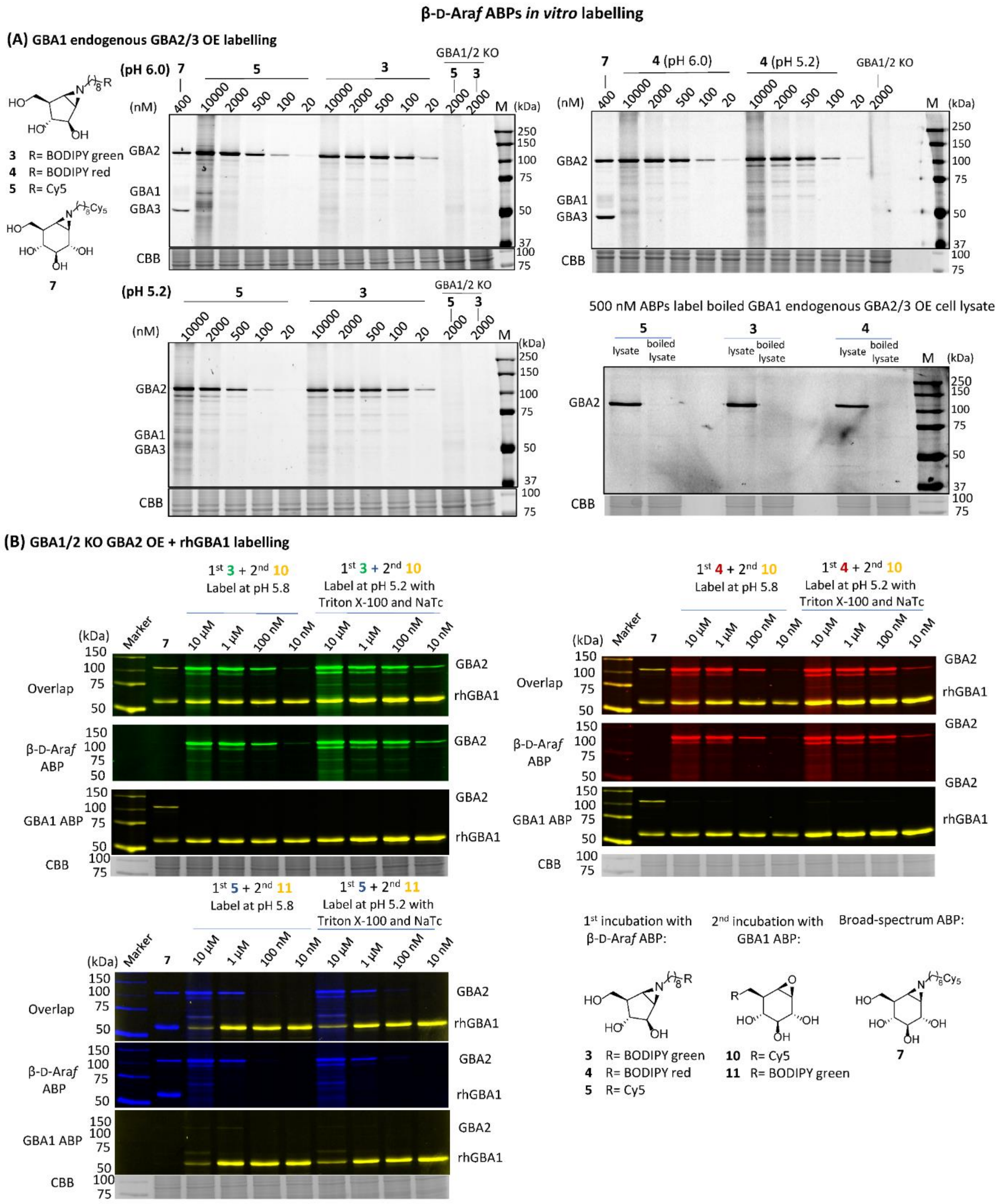

Figure S3-1. Fluorescent gel images of *in vitro* labelling of β-D-Ara*f* aziridine ABPs **3**-**5** towards β-glucosidases. A) β-D-Ara*f* ABPs **3**-**5** label β-glucosidases *in vitro* after 30 min incubation at 37 °C with HEK293T cell lystates containing endogenous GBA1 and overexpressed GBA2/GBA3. B) β-D-Ara*f* ABPs **3**-**5** label a mixture of HEK293T GBA1/2 KO GBA2 OE cell lysate spiked with recombinant human GBA1 (3 ng) at optimal conditions for GBA2 (left, pH 5.8) or for rhGBA1 (right, pH 5.2). Lysate mixture was first incubated with β-D-Ara*f* ABPs for 30 min at 37 °C, and rhGBA1 was subsequently labelled by ABP **10** at 500 nM (for **3** and **4**) or ABP **11** at 500 nM (for **5**). As a control, in lane '**7**', only broad spectrum ABP **7** was added to show the presence of GBA2 and rhGBA1. To label rhGBA1 McIlvaine buffer (150 mM, pH 5.2) with 0.1% (v/v) Triton X-100 and 0.2% (w/v) sodium taurocholate (NaTc) was used. To label GBA2, McIlvaine buffer (150 mM, pH 5.8) was used.

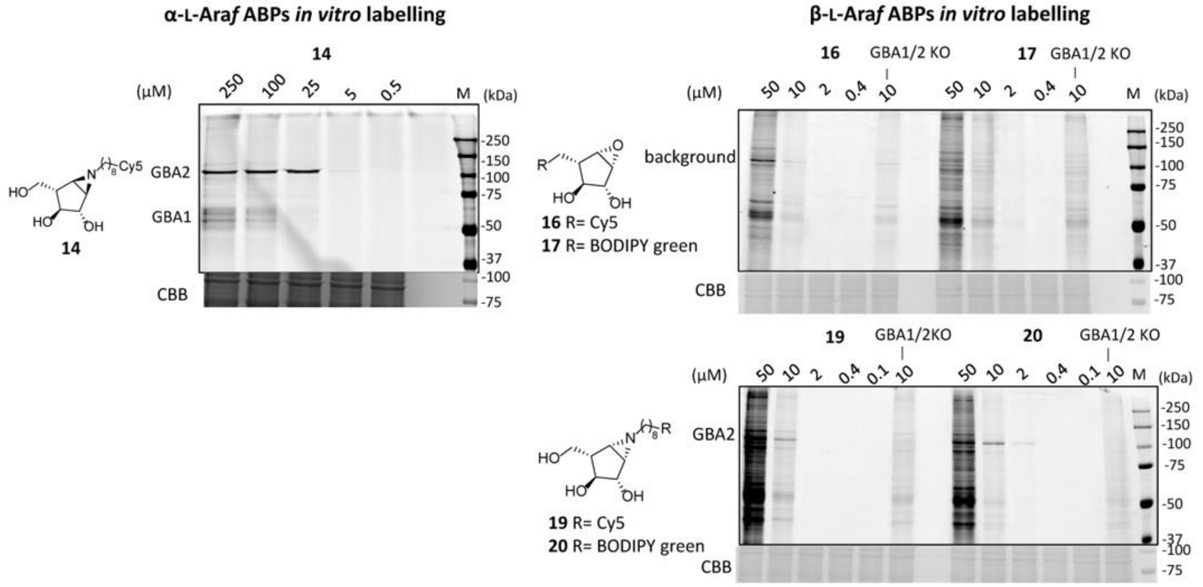

Figure S3-2. Fluorescent gel images of *in vitro* labelling of β-glucosidases after 30 min incubation at 37 °C with α-L-Ara*f* and β-L-Ara*f* ABPs in HEK293T cell lystates containing endogenous GBA1 and overexpressed GBA2/GBA3.

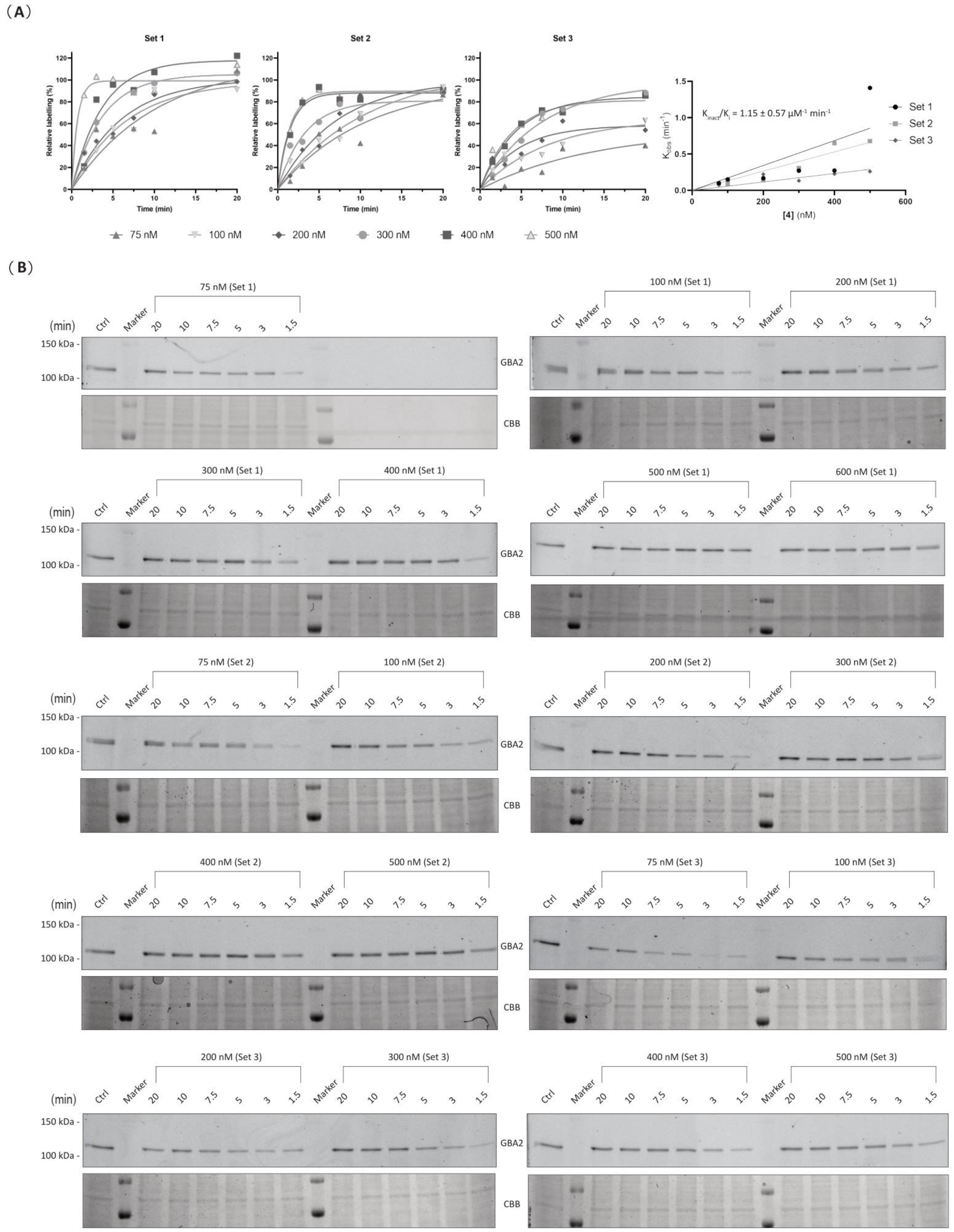

Figure S4-1. Determination of labelling kinetics for ABP **4** on GBA2. Lysate of HEK293T GBA1/2 KO GBA2 OE cells were labelled with ABP **4** *in vitro* at different concentrations and for different times. A) Percentage of GBA2 labelling by ABP **4** (left graphs), data was quantified from three independent sets of fluorescent gels under the depicted conditions. From these graphs a rate constant *k* was derived for each concentration of ABP **4**. Afterwards *k* vs [ABP **4**] (right graph) was plotted and a linear curve was fitted to derive the kinetic parameters (k_{inact}/K_I ratio). B) Wet gel slabs used to determine relative labelling of GBA2 by ABP **4** and their corresponding Coomassie brilliant blue protein stains.

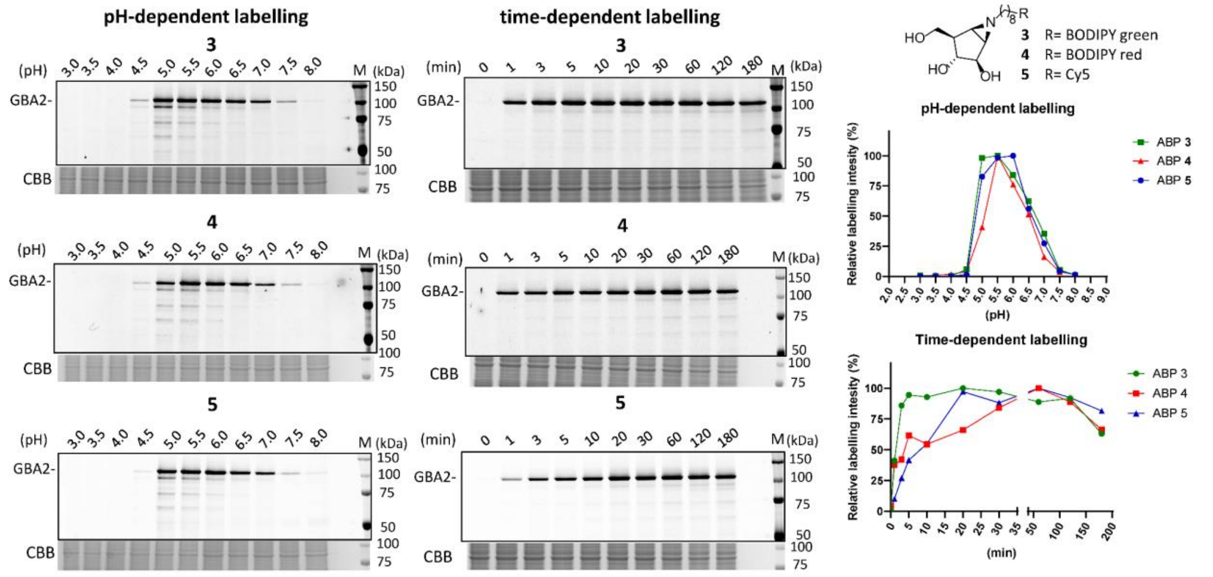

Figure S4-2. Fluorescent gel images of *in vitro* pH- or time-dependent labelling of GBA2 after incubation of β-D-Ara*f* ABPs **3-5** (500 nM) in lysates of HEK293T cells containing endogenous GBA1 and GBA2/GBA3 OE.

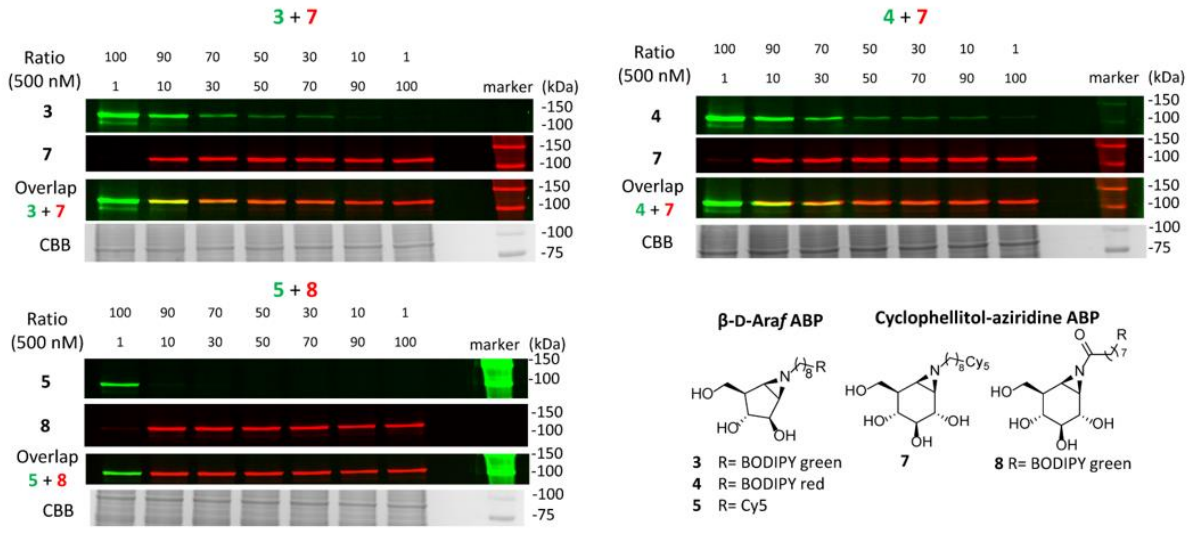

Figure S5. Fluorescent gel images of *in vitro* labelling of GBA2: comparison between β-D-Ara*f* aziridine ABPs **3-5** and cyclophellitol-aziridine ABPs **7** and **8**. GBA1/2 KO GBA2 OE HEK293T cell lysates were labeled with equimolar total amounts of β-D-Ara*f* ABP **3-5** and cyclophellitol aziridine ABP **7** (or **8**) mixtures in varying ratio (mixed ABP final concentration was 500 nM). Results indicate that ABP **3** and **4** have equal GBA2 labelling efficiency with ABP **7** (at 90:10 ratio of β-D-Ara*f* aziridine ABP: cyclophellitol-aziridine ABP), and ABP **5** was able to label GBA2 at 100:1 ratio when competing with ABP **8**.

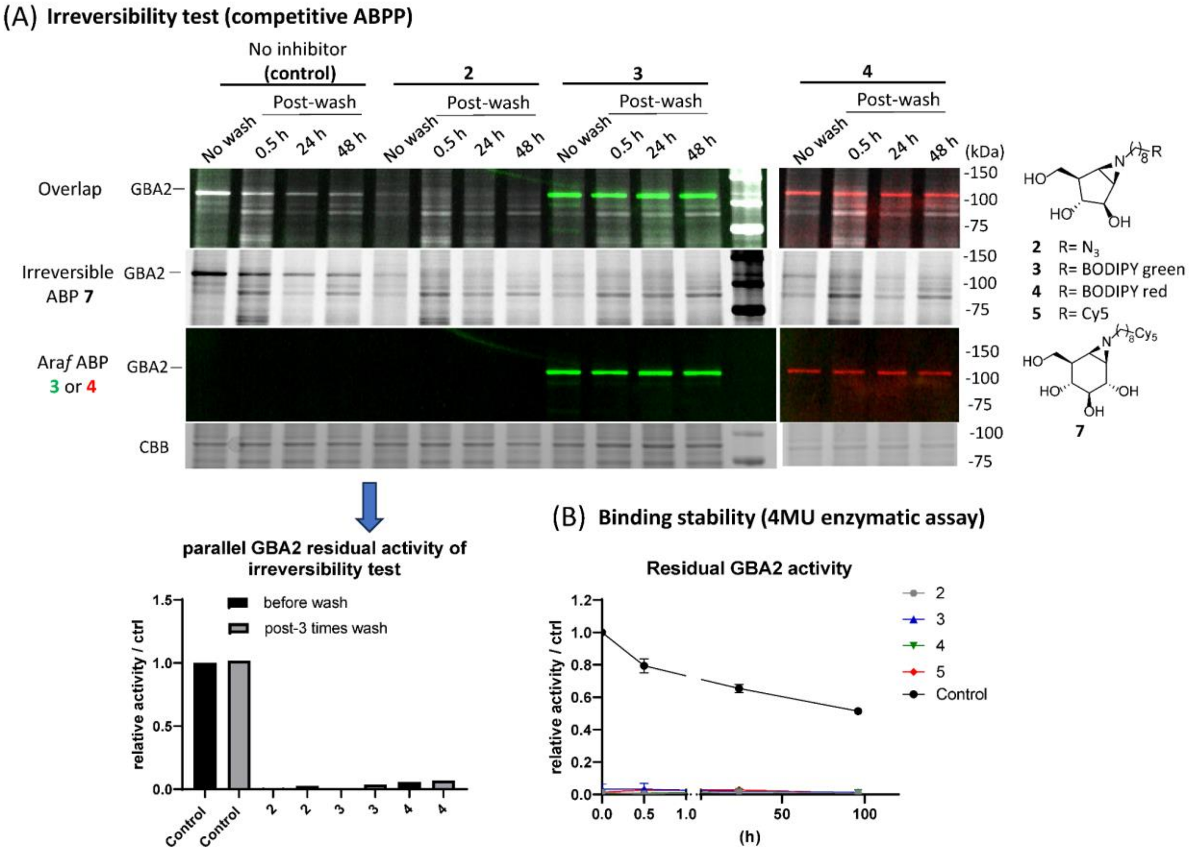

Figure S6. β-D-Ara*f* aziridine probes label GBA2 in an irreversible manner. A) Fluorescent gel images of GBA2 lysate were pre-treated with β-D-Ara*f* compound, then desalted, washed and subsequently labelled with 1 μM of ABP **7** for competition. Control: incubation with 0.5% DMSO followed by incubation with 1 μM of ABP **7**. Lanes: No wash = pre-treated GBA2 without the wash step and incubated with ABP **7** for 30 min at 37 °C; Post-wash = pre-treated GBA2 after 3 consecutive washing steps followed by incubation with ABP **7** for the described time. The 24 h and 48 h samples were incubated at 4 °C for enzyme stability reasons, whereas the 0.5 h set were incubated with ABP **7** for 30 min at 37 °C. A parallel 4MU enzymatic assay was conducted by taking a part of sample and evaluating enzyme activity after wash (result was showed below). B) Binding stability revealed by 4MU enzymatic assay. After washing, lysate was stored at 4°C until the time point for activity measurement was reached. Control (black dots) = lysate without inhibitor addition, 0 h = measured activity of lysate prior washing of centrifugal desalting column. Time (h) > 0 = measured activity of lysate at a certain time point after washing steps.

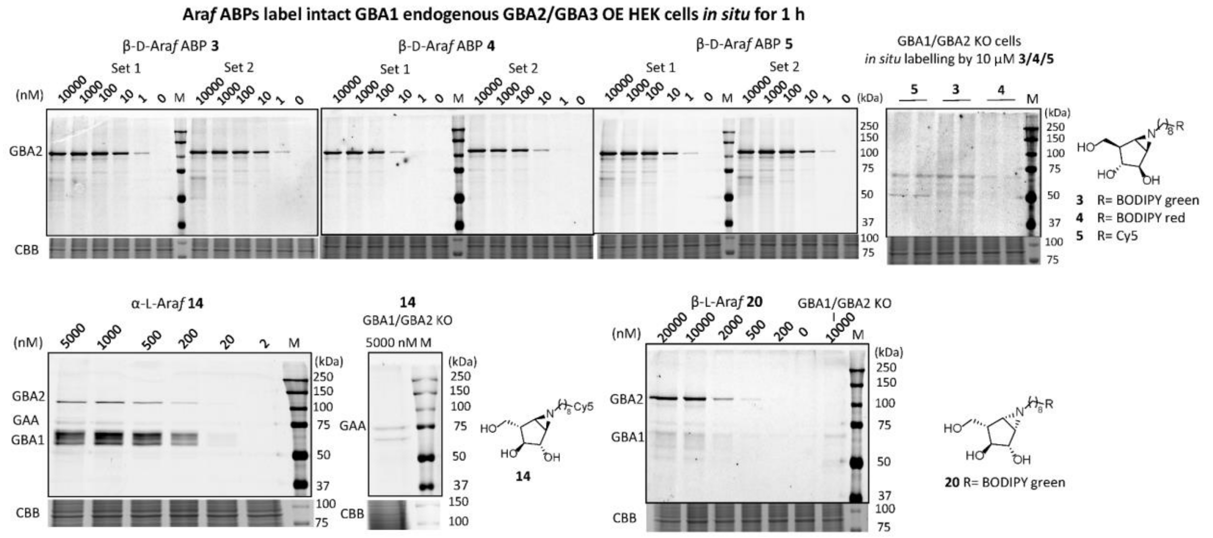

Figure S7. Fluorescent gel images of *in situ* labelling of intact HEK293T cells (GBA1 endogenous and GBA2/GBA3 OE) by arabinofuranosyl cyclitol configured ABPs **3-5**, **14** and **20** after 1 h incubation. GAA = Lysosomal α glucosidase (EC 3.2.1.20, CAZy GH31).

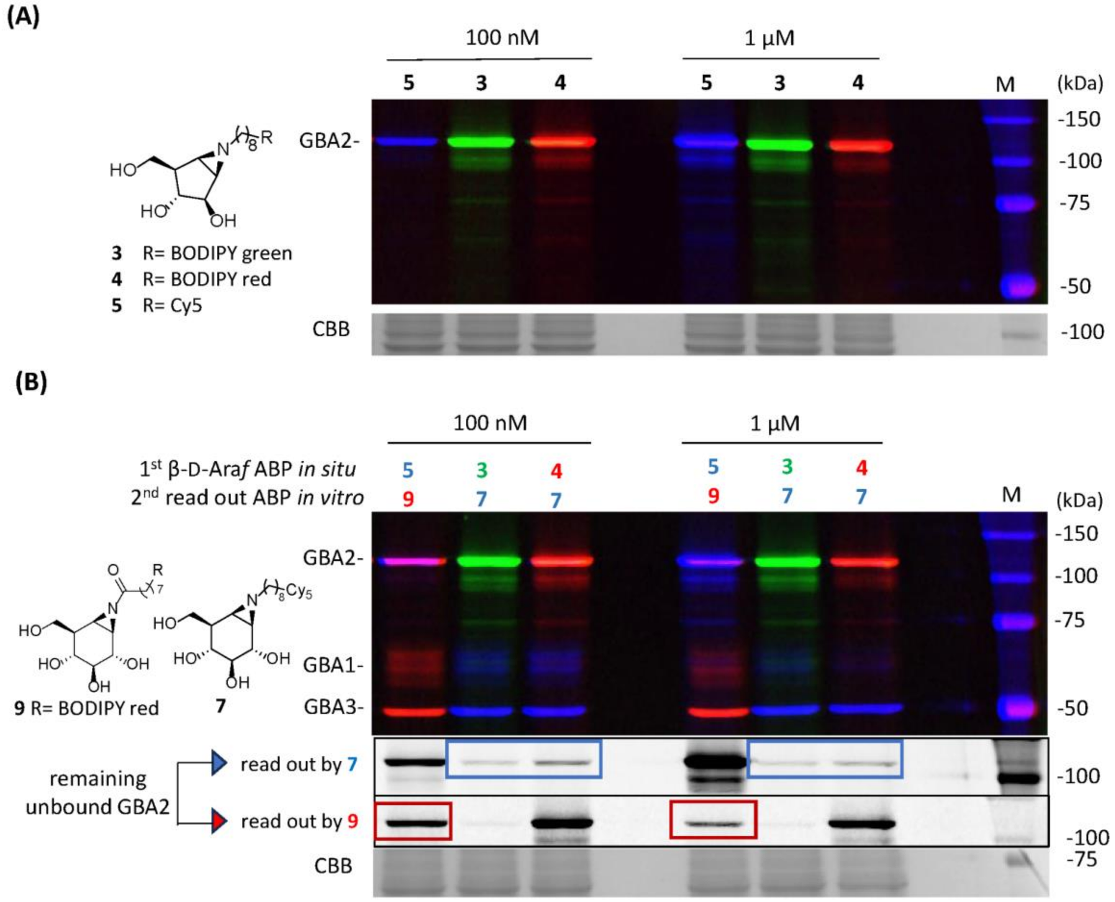

Figure S8. Fluorescent gel images of *in situ* labelling of intact HEK293T cells (endogenous GBA1 and GBA2/GBA3 OE) by β-D-Ara*f* aziridine ABPs **3-5** after 24 h incubation. Intact HEK293T cells were treated with β-D-Ara*f* ABPs for 24 h at 37 °C with 7% CO_2, then cells were collected and lysed for labelling studies with or without residual β-glucosidases activity read out. A) β-D-Ara*f* ABPs **3-5** labelled intact cells without additional labelling of residual β-glucosidases. B) β-D-Ara*f* ABPs **3-5** labelled intact cells with subsequent labelling of residual β-glucosidases by broad-spectrum cyclophellitol-aziridine ABP **7** (for **3** and **4**) or **11** (for **5**) at 500 nM.

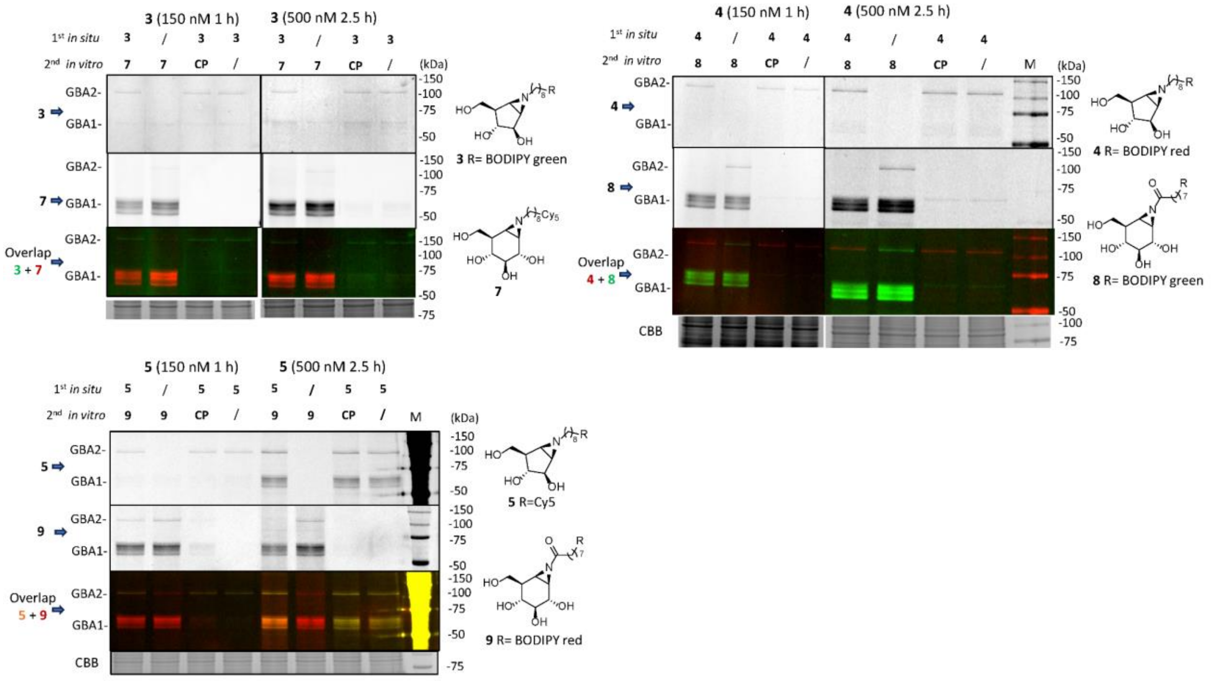

Figure S9. Fluorescent gel images of *in situ* labelling of wild-type HEK293T cells by β-D-Ara*f* aziridine ABPs **3-5**. β-D-Ara*f* ABPs were firstly incubated with intact wild-type HEK293T cells for 1 or 2.5 h (1st incubation) and then cells were harvested. Lysis buffers containing different competitors (1 µM ABP or cyclophellitol inhibitor (CP, 1 µM for 150 nM set, 2 µM for 500 nM set)) was added to the harvested cells, cells were lysed, and lysate was incubated on ice for 30 min and additional 30 min at 37 °C (2nd incubation). Labelling of residual β-glucosidases was performed using ABPs **7** (for **3**), **8** (for **4**), or **9** (for **5**) at 500 nM.

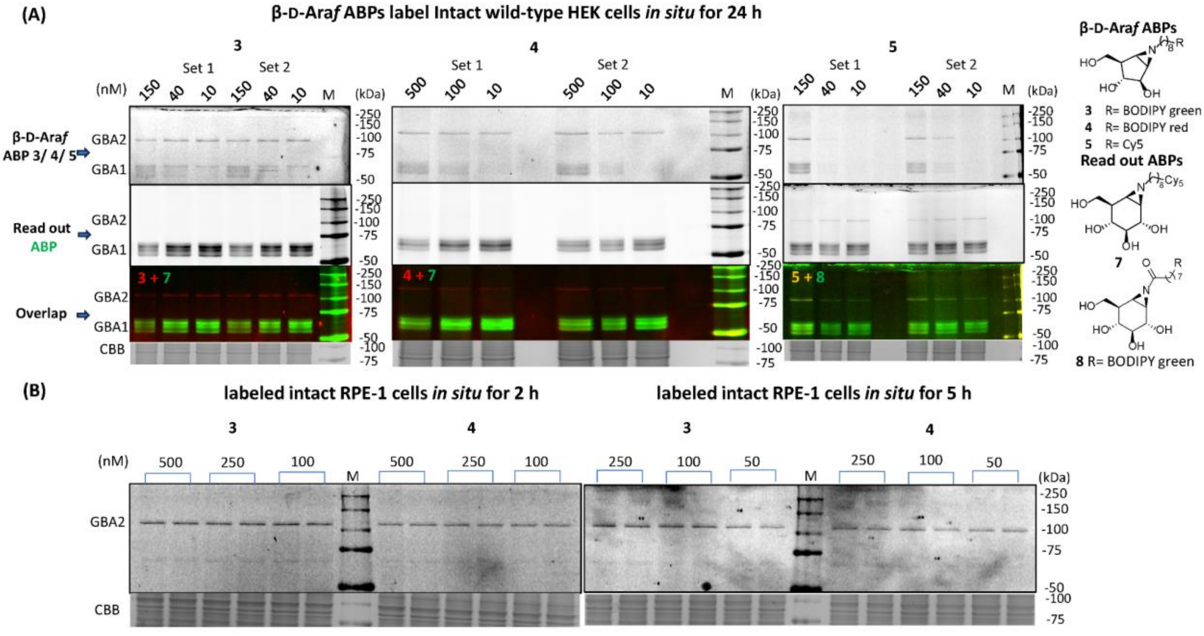

Figure S10. Fluorescent gel images of A) intact HEK293T wild-type cells labeled by β-D-Ara*f* aziridine ABPs **3**-**5**. Intact HEK293T wild-type cells were incubated for 24 h with the described concentrations of β-D-Ara*f* ABPs, cells were collected, lysed, and residual active GBA2 and GBA1 were labelled by **7** (for **3** and **4**) or **8** (for **5**) at 500 nM. B) intact RPE-1 cells labeled by described concentrations of ABPs **3**-**5** for 2 h or 5 h.

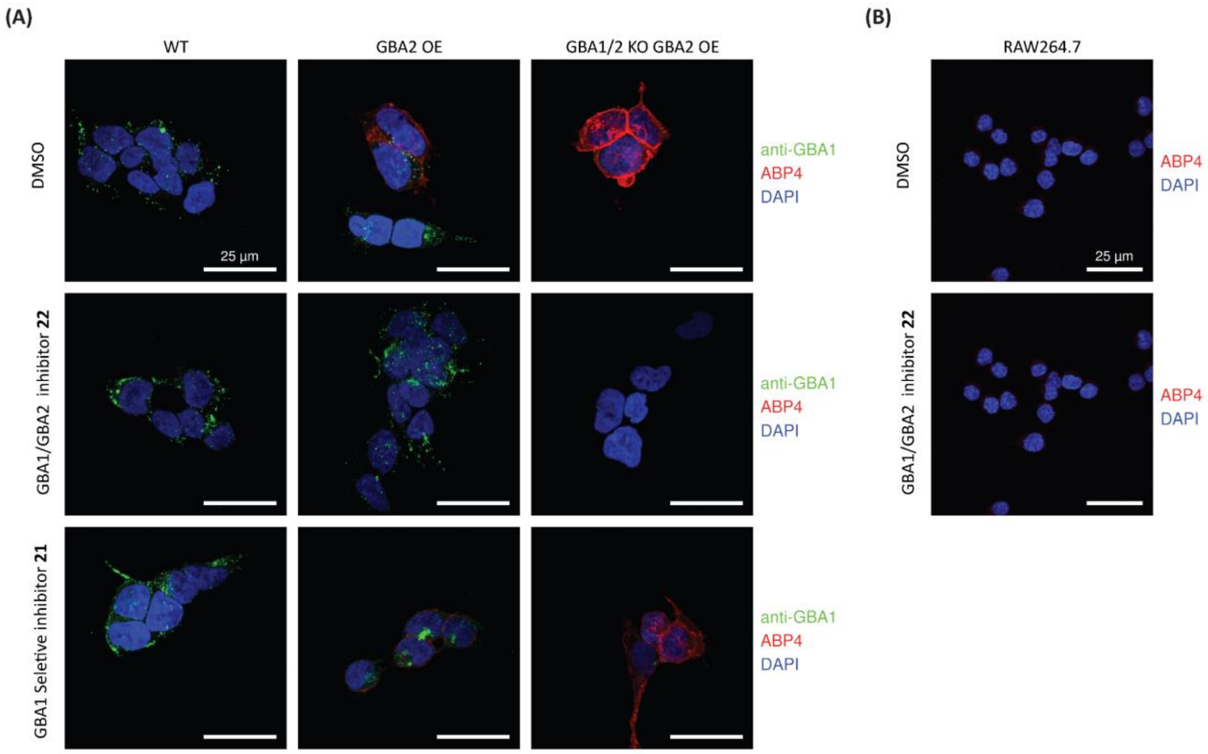

Figure S11. Confocal fluorescence microscopy of A) HEK293T wild-type (WT), GBA2 overexpression (OE) or GBA1/2 knock out (KO) + GBA2 overexpression (OE) cells. Cells were pre-treated with either DMSO, GBA1/GBA2 inhibitor **22** or GBA1 selective inhibitor **21**, and afterwards incubated with β-D-Ara*f* aziridine BODIPY-red ABP **4** (50 nM) for 2 h. Subsequently, cells were fixed and stained for GBA1 (Green) with an anti-GBA1 antibody. B) RAW264.7 cells pre-treated with either DMSO or GBA1/GBA2 inhibitor **22** and incubated β-D-Ara*f* aziridine ABP **4** for (50 nM) for 2 h. In all samples nuclei were stained with 10 µg/ml DAPI (Blue).

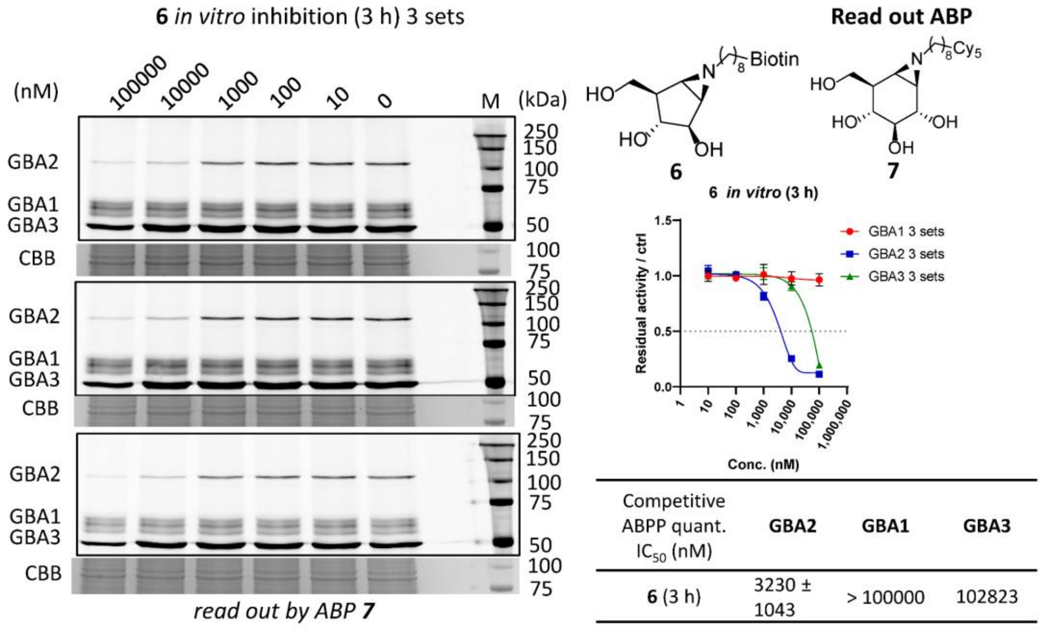

Figure S12. Assessment of activity and selectivity of biotin-tagged ABP **6** using an *in vitro* competitive ABPP assay. HEK293T cells containing endogenous GBA1 and GBA2/GBA3 OE lysate was incubated with ABP **6** for 3 h at 37 °C, and then the residual active β-glucosidases were subsequently labelled by ABP **7**. Competitive ABPP allow for the determination of apparent IC_{50} values by quantification of the fluorescence intensity of ABP **7** as described in experimental section.

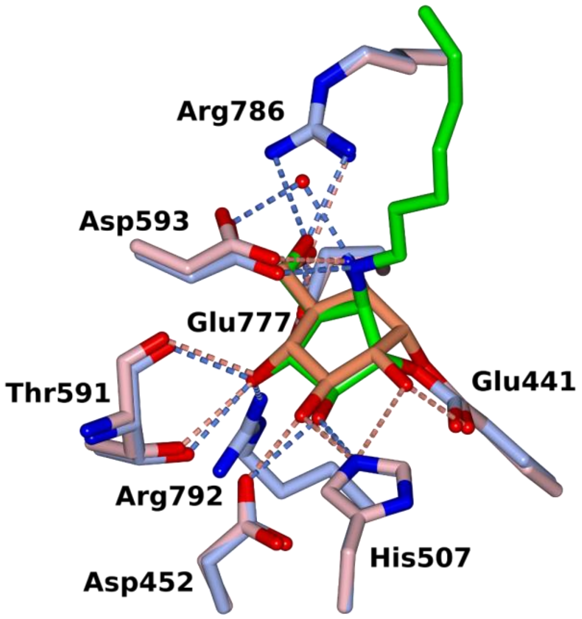

Figure S13. Overlay of structures of *Tx*GH116 complexes with cyclophellitol aziridine (8R06.pdb) and compound **2** (8R1M.pdb). C atoms are shown in orange for cyclophellitol aziridine and light pink for interacting side chains (with hydrogen bonds as coral dashed lines), and in green for **2** and ice blue for interacting side chains (with hydrogen bonds as blue dashed lines).

2 Biochemistry

2.1 Materials

Recombinant human GBA1 (rhGBA1, Imiglucerase) was kindly provided by Sanofi Genzyme (Cambridge, MA, USA). 4-Methylumbelliferyl-β-D-glucopyranoside substrates were purchase from Glycosynth (Warrington Cheshire, UK). Protein concentration was measured using the Pierce BCA assay kit (Thermo Fisher Scientific, Waltham, MA, USA). Harvested cells (cell pellets), cell lysates and tissue homogenates not used directly were stored at -80 °C.

2.2 Cell culture

HEK293T (CRL-3216™) cells were purchased from ATCC (Manassas, VA, USA). HEK293T cells were cultured in DMEM medium (Sigma-Aldrich), supplied with 10% (v/v) FCS, 0.1% (w/v) penicillin/streptomycin and 1% (v/v) Glutamax, under 37 °C and 5% or 7% CO_2. RPE-1 cells were purchased from ATCC (hTERT RPE-1, CRL-4000™). RPE-1 cells were cultured in HAMF12-DMEM medium (sigma-aldirch), supplied with 10% (v/v) FCS, 0.1% (w/v) penicillin/streptomycin and 1% (v/v) Glutamax, under 37 °C and 5% CO_2.

2.3 Generation of cells genetically modified for β-glucosidase expression

For overexpression of the different β-glucosidases, "wildtype" HEK293T cells, HEK293T cells with GBA2 knockout (KO) or HEK293T cells with both GBA1 and GBA2 KO were used, following by the overexpression (OE) of targeted β-glucosidases using the constructs as described in references.[1,2] Either GBA2 or GBA3 were overexpressed in GBA1/GBA2 KO HEK293T (generated by the CRISPR/Cas9 method[2]) to generate GBA1/2 KOs with GBA2 (or GBA3) for overexpression in HEK293T cells as indicated, or using "wildtype" HEK293T cells containing endogenous GBA1 and GBA2 to overexpress both GBA2 and GBA3, these were generated as described in our previous report.[1,2]

2.4 Cell lysis

Cells were cultured with the method described above to 80-90% confluency, and were isolated, washed with Dulbecco's phosphate buffered saline (PBS) and subsequently collected using the following method.[3] HEK293T and RPE-1 cells were easily detached from the culture plate by trypsin treatment after which some DMEM medium was added. The cells were collected by pipetting and DMEM medium was removed by centrifuging. The cells were washed with cold PBS three times, after which they were centrifuged to remove PBS and then stored at –80 °C for further use or lysed in potassium phosphate buffer (K_2HPO_4-KH_2PO_4, 25 mM, pH 6.5, supplemented with 0.1% (v/v) Triton X-100 and protease inhibitor cocktail (EDTA-free, Roche, Basel, Switzerland)) by sonication with a Polytron PT 1300D sonicator (Kinematica, Luzern, Switzerland). After sonication, lysed mixtures were centrifuged at 10,000 x *g* for 3 min at 4°C, and the supernatants where desired β-glucosidases exist were used for the experiment.

2.5 4MU fluorogenic substrate assay for apparent IC_{50} determination

4MU fluorogenic substrate assays (using recombination human rhGBA1 or lysates of HEK293T cells) were conducted in 96-well plates at 37 °C. Samples were diluted with McIlvaine buffer (150 mM citric acid-Na_2HPO_4) to a final volume of 25 µL, at the appropriate pH for each enzyme. Assays were performed by incubating the samples with 100 µL 4-methylumbelliferyl-β-D-glucopyranoside substrate diluted in McIlvaine buffer for a period of 30 min. Enzyme activities and apparent IC_{50} values were determined by subtraction of the background (measured for incubations without enzyme) as previously described.[4] The substrate mixtures used for each enzyme were as follows: GBA1: 3.75 mM 4-MU-β-D-glucopyranoside in McIlvaine buffer (150 mM, pH 5.2, supplemented with 0.2% (w/v) sodium taurocholate, 0.1% (v/v) Triton X-100, 0.1% (w/v) bovine serum albumin (BSA)); GBA2: 3.75 mM 4 MU-β-D-glucopyranoside in McIlvaine buffer (150 mM, pH 5.8); GBA3: 3.75 mM 4-MU-β-D-glucopyranoside in McIlvaine buffer (150 mM, pH 6.0). To determine apparent IC_{50}: For GBA1, 3.16 ng (53 fmol) of rhGBA1 (recombinant human GBA1, Imiglucerase) was prepared in 12.5 µL McIlvaine buffer (150 mM, pH 5.2, supplemented with 0.1% (v/v) Triton X-100, 0.2% (w/v) sodium taurocholate, and 0.1% (w/v) bovine serum albumin (BSA)). The enzyme was incubated with 12.5 µL

of inhibitors diluted in McIlvaine buffer (150 mM, pH 5.2) at 37 °C for 30 min or 3 h. For GBA2, lysates of GBA1/GBA2 KO HEK293T cells with GBA2 overexpression were prepared in 12.5 µL McIlvaine buffer (150 mM, pH 5.8), and incubated with 12.5 µL of inhibitors diluted in McIlvaine buffer (150 mM, pH 5.8) at 37 °C for 30 min or 3 h. For GBA3, lysates of GBA1/GBA2 KO HEK293T cells with GBA3 overexpression were prepared in 12.5 µL McIlvaine buffer (150 mM, pH 6.0), and incubated with 12.5 µL of inhibitors diluted in McIlvaine buffer (150 mM, pH 6.0) at 37 °C for 30 min or 3 h. Subsequently, the samples were incubated with 100 µL 4-methylumbelliferyl-β-D-glucopyranoside substrates dissolved in McIlvaine buffer described above for a period of 30 min. After stopping the enzyme reaction with 200 µL 1 M NaOH-glycine (pH 10.3), 4-methylumbelliferone fluorescence was measured with a fluorimeter LS55 (Perkin Elmer, Waltham, MA, USA) with λ_{EX} 366 nm and λ_{EM} 445 nm. The IC_{50} value was calculated using Graphpad Prism 9.0 with the Nonlinear regression (curve fit) - [Inhibitor] vs. response - Variable slope (four parameters) equation.

2.6 Visualization of ABP labelling towards β-glucosidases by activity-based protein profiling (ABPP) with SDS-PAGE

β-Glucosidases were labeled with excess of fluorescent ABPs at optimum conditions at 37 °C for 30 min (if not otherwise stated). The total sample volume was 20–40 µL with 0.5–1% DMSO concentration in appropriate pH McIlvaine buffer (150 mM). To label lysates containing all β-glucosidases (GBA1, GBA2 and GBA3), ABP **7** was used at pH 6.0 in McIlvaine buffer. To label lysate only containing GBA2 (GBA1/2 KO and GBA2 OE cell lysate), ABP (**3**-**5**, or **7**) was incubated at pH 5.8 in McIlvaine buffer was used. To label rhGBA1 (Imiglucerase), ABP (**10** or **11**) at pH 5.2 in McIlvaine buffer supplemented with 0.1% (v/v) Triton X-100 and 0.2% (w/v) sodium taurocholate were used. After incubation with the corresponding ABPs, proteins were denatured by boiling the samples with 5× Laemmli buffer (50% (v/v) 1 M Tris-HCl, pH 6.8, 50% (v/v) 100% glycerol, 10% (w/v) DTT, 10% (w/v) SDS, 0.01% (w/v) bromophenol blue, diluted into 1x for use) for 5 min at 98 °C, and separated by electrophoresis on 10% SDS-PAGE gels run at 90 V for 1.0-1.5 h. Wet slab-gels were imaged using the Typhoon FLA 9500 scanner (GE Healthcare, Eindhoven, The Netherlands) at λ_{EX} 473 nm and λ_{EM} ≥ 510 nm for BODIPY green fluorescence ; at λ_{EX} 532 nm and λ_{EM} ≥ 575 nm for the BODIPY red fluorescence ; at λ_{EX} 635 nm and λ_{EM} ≥ 665 nm for Cy5 fluorescence. Afterwards, wet slab-gels were stained by Coomassie brilliant blue (CBB) G250 or R250 for total protein staining and scanned with a ChemiDocTM MP Imaging System (Bio-Rad, Veenendaal, The Netherlands).

2.7 Determination of kinetic parameters of ABP 4

The kinetic parameters of ABP **4** for GBA2 was determined by SDS-PAGE analysis in lysates of HEK293T GBA1/2 knock out GBA2 overexpression cells in which the relative intensity of the band on wet gel slabs (compared to labelling control) was used to calculate the inactivation parameters of GBA2. Lysates were made as previous cell lysis description. The lysates were incubated with different concentrations of ABP **4** (concentration were chosen so 100% labelling could be achieved for any given concentration) for multiple incubation times. 4 µg of lysate diluted in 150 mM McIlvaine buffer (pH 5.8) was added in 1.5 ml Eppendorf tube and 2.22 µl of a 10x concentrated ABP **4** (yielding final probe concentrations of 500, 400, 300, 200, 100 and 75 nM at constant 0.5 % DMSO) was added for each incubation time (20, 10, 7.5, 5, 3 and 1.5 min) at 37 °C. The 100% labelling control was incubated with 1 µM ABP **4** for 60 min at 37 °C. The labelling was stopped by the addition of 4x Laemmli sample buffer and subsequent boiling of samples at 98 °C for 5 min. After SDS-PAGE separation, wet gel slabs were scanned on Typhoon FLA9500 at λ_{Ex} = 532 nm and λ_{Em} ≥ 575 nm for the BODIPY-red fluorescence. Relative intensity of bands on wet gel slabs was determined in Fiji ImageJ. First the relative intensity of the bands was plotted against incubation time for each concentration of ABP **4** and the plotted data were fitted with one-phase exponential association function to obtain the rate constant *k* at each ABP **4** concentration. Secondly, the *k* values were plotted against ABP **4** concentration. Since the labelling kinetics of ABP **4** were extremely fast, no plateau could be observed in this graph so only the kinetic parameter K_{inact}/K_I could be derived by using linear curve

fitting. All kinetic calculation were done in GraphPad Prism 9.0. Afterwards, wet slab gels were stained by Coomassie brilliant blue G250 for total protein staining and imaged with ChemiDoc MP.

2.8 β-Glucosidase inactivation visualized by competitive ABPP with SDS-PAGE

In general, samples containing GBA1, GBA2 or GBA3 (see the exact constitution of these samples in the described case) are first incubated (*in vitro* or *in situ*) with the inactivator compounds, subsequently, the fluorescent readout ABPs were added and incubated with the mixture to reveal the residual active β-glucosidase enzymes. Briefly, for *in vitro* β-glucosidase inactivation, cell lysates containing β-glucosidases were first incubated with inactivators *in vitro* for 30 min (or for described time), after which a fluorescent readout ABP was added to reveal the residual active β-glucosidases and samples were incubated for 30 min at 37 °C, following the procedures described above for protein denaturation, SDS-PAGE and fluorescence scanning. For *in situ* β-glucosidase inactivation, intact living HEK293T cells expressing β-glucosidases were first incubated with fluorescent ABP **3**-**5** (*in situ*) for 24 h at 37°C under 7% CO_2, then cells were harvest and lysed as description in cell lysis. Subsequently, a fluorescent readout ABP (**7**-**9**) was added and samples were incubated for 30 min at 37°C *in vitro*, followed by procedures described above for protein denaturation, SDS-PAGE and fluorescence scanning. Of note, a fluorescent readout ABP with different scanning wavelength from the inactivator with a fluorophore reporter should be chosen. For example, if ABP **3** with BODIPY-green reporter is first used to inactivate β-glucosidases, the readout ABP should be ABP **7** with Cy5 reporter or ABP **9** with BODIPY-red reporter. Competitive ABPP quantified apparent IC_{50} values were determined according to the ABP-emitted fluorescence of the read out ABP quantified by ImageQuant (GE Healthcare, Chicago, IL, USA) and calculated with Graphpad Prism 9.0 using Analyze - Nonlinear regression (curve fit) – One phase association.

2.9 Irreversibility evaluation by competition with irreversible ABP

The irreversible inhibition of the compounds was also evaluated using lysates of HEK293T GBA1/2 KO and GBA2 OE. A total of 60 µg of lysate was incubated with or without 1 µM β-D-Ara*f* cyclitol aziridines for 1 h at 37 °C, a part of the lysate was taken and used for a parallel activity measurement using a fluorogenic 4-MU assay. Subsequently, the lysate mixture was washed by passing through Zeba™ spin desalting columns with 40k MWCO (Thermo Scientific™) to remove excess compounds. The wash was repeated every 2 h, with 3 washes conducted in total, and the lysate was kept on ice or at 4 °C to maintain the activity during the wash steps. After the last wash step, the same volume of lysate (25 µL) was taken and incubated with irreversible β-glucosidase ABP **7** at 1 µM for 30 min at 37 °C, or for 24 h or 48 h at 4 °C. Then the competition result was analysed by SDS-PAGE fluorescence scanning as described above. In parallel, activity measurements were performed to show GBA2 activity recovery after desalting washing (Figure S6).

2.10 Stability of β-D-Ara*f* aziridine binding towards GBA2

A total of 90 µg of lysate of HEK293T GBA1/2KO GBA2 OE cells in potassium phosphate buffer (25 mM, pH 6.5, supplemented with 0.1% (v/v) Triton X-100 and protease inhibitor cocktail) was diluted with 60 µL McIlvaine buffer (150 mM, pH 5.8) and incubated with or without 1 µM β-D-Ara*f* aziridine ABP for 1 h at 37 °C. The total 100 µL sample was passed through a desalting column as described above and samples were kept at 4 °C until GBA2 activity was measured by 4MU-β-D-Glc fluorogenic substrate assay at time points 0.5 h, 24 h and 96 h. The 4MU-β-D-Glc fluorogenic substrate assays for GAB2 activity measurement were performed in triplicate with 10 µL sample as enzyme source, and the activity of GBA2 incubated without inhibitors was used as a control.

2.11 Reactivity of β-D-Ara*f* aziridines towards GBA2 mutants

For overexpression of GBA2 mutants, HEK293T GBA1/GBA2 KO cells were used. GBA2-E527G, GBA2-D677G, or GBA2- E527G/D677G double mutants containing a myc tag were generated as described previously for COS-7 cells.[5] Next, 500 nM β-D-Ara*f* cyclitol aziridine ABPs **3-5** or cyclophellitol-aziridine ABP **7** were incubated with the corresponding lysate for 30 min at 37 °C, followed by SDS-PAGE and fluorescence scanning as described above. Subsequently, proteins in the wet slab gels

were transferred to nitrocellulose membrane for western blotting. Ponceau S staining was used to show total protein loading. Mouse anti myc (α-myc) primary antibody and secondary antibody consisting of donkey anti mouse with Alexa 488 or goat anti mouse Alexa 532 were used to visualize GBA2 mutants with the myc tag.

2.12 Reactivity of β-D-Ara*f* aziridines towards GBA2 orthologue in other species

β-D-Ara*f* aziridine ABP **3**-**5** or ABP **7** was incubated with homogenates of zebrafish (*Danio rerio*) larvae or mice brain for 1 h at 37 °C. The homogenates were obtained as follows. Zebrafish larvae were kept at a constant temperature of 28.5°C and raised in egg water (60 µg L-1 sea salt, Sera Marin) for 115 h, larvae were collected and sacrificed for making homogenates, a total 10-20 µg larvae homogenates were used. Extracts of mouse brain were obtained from existing mice extraction stocks that were stored at –20 °C, a total of 10-20 µg mouse brain homogenates were used. The zebrafish homogenates were passed through a desalting column (in the same way as described for the irreversibility evaluation above) to remove interference from biological pigments when scanning at λ_{EX} 473 nm and $\lambda_{EM} \geq 510$ nm for BODIPY green fluorescence.

2.13 *In situ* intact cells labelling and inhibition assays

Confluent HEK293T cells expressing human endogenous GBA1 and GBA2/GBA3 OE, wild-type HEK293T cells or wild-type human retinal pigment epithelial-1 (RPE-1) cells were cultured in 12-well plates for duplicates or triplicates with (or without) compounds (**3**-**5**) for the described incubation times using the conditions as described for the cell culture. The cells were harvested as described in the cell lysis method. After determination of the protein concentration by BCA assay, the lysates were adjusted to 10 µL by addition of potassium phosphate buffer in order to normalize the amount of protein loaded (to 10-25 µg total protein per measurement), and subjected to the ABPP as described above.

2.14 *In situ* visualization of GBA2 for confocal microscopy

HEK293T WT, GBA2 overexpression (GBA1 endogenous and GBA2 OE) and GBA1/GBA2 knockout (KO) GBA2 overexpression (GBA1/2 KO GBA2 OE) cells were cultured in DMEM High Glucose (Capricon Scientific) supplemented with 10% fetal calf serum, 1% (v/v) GlutaMax and 0.1% (w/v) penicillin/streptomycin.[2,6] Cells were grown to approximately 75% confluence on Ø15 mm glass coverslips coated with 0.1 mg/ml poly-L-Lysine.

Samples were pretreated for 16 h with 100 nM of a GBA1 inhibitor (**21**[8], 0.5% DMSO), 500 nM of a GBA1 and GBA2 inhibitor (**22**[9], 0.5% DMSO) or with 0.5% DMSO alone. GBA2 was subsequently incubated by the addition of 50 nM ABP **4** (BODIPY-Red) to cells for 2 h at 37 °C with 5% CO_2. Unbound probe was washed away with PBS and cells were fixed with 4% paraformaldehyde (Alfa Aesar)/PBS for 15 min at RT. After fixation the cells were washed with PBS and, if antibody stain was needed, directly permeabilized with 0.1% Tween20 (Sigma-Aldrich)/TBS for 30 min at RT. After gentle washing in PBS, the samples were blocked using 3% (w/v) BSA (Sigma-Aldrich)/PBS for 30 min at RT. Immunofluorescence staining was performed with Mouse-anti-GBA1 (8E4, generated in Aerts-lab[10] 1:250 in 3% BSA-PBS and visualized with Donkey-anti-Mouse-Alexa647 (Molecular Probes) 1:1000 in 3% BSA-PBS. Nuclei were stained with 10 µg/ml DAPI (Sigma-Aldrich). Afterwards the samples were mounted on ProLong Diamond (Molecular Probes) and imaged on a Nikon Eclipse Ti2 confocal microscope with a 100x/1.49 Numerical Aperture SR HP Apo TIRF oil immersion objective equipped with a PMT detector.

HEK293T GBA2 knockout cells were grown on Ø15 mm glass coverslips coated with 0.1 mg/ml poly-L-Lysine until 30% confluence was reached and transfected with mouse-GBA2 C-terminally tagged with GFP using PEI.[7] The cells transiently overexpressing mouse-GBA2-GFP were then stained by incubation with 50 nM ABP **4** for 2 h at 37 °C with 5% CO_2, after washing and fixation as described above, samples were imaged by confocal microscopy.

RAW264.7 cells were cultured in DMEM High Glucose at 37°C under 5% CO_2, supplemented with 10% fetal calf serum, 1% (v/v) GlutaMax and 0.1% (w/v) penicillin/streptomycin. RAW264.7 cells were grown to approximately 75% confluence on Ø15 mm glass and pretreated for 16 h with 500 nM

inhibitor **22** or with 0.5% DMSO. Afterwards, cells were stained by the addition of 50 nM ABP **4** for 2 h incubation, and followed by washing and fixation procedures for confocal microscopy as described above.

2.15 Expression, purification and crystallization of *Tx*GH116

A construct consisting of *Tx*GH116Δ1-18 cloned into pET30a was transformed into *E. coli* BL21(DE3) and used for the expression of *Tx*GH116 and its purification based on the method described.[11] *Tx*GH116 at 1 mg/ml in 20 mM Tris–HCl pH 8.0, 150 mM NaCl was crystallised in a sitting drop plate, in a 2:1 ratio to the well solution, which consisted of 20 % (w/v) polyethylene glycol 3000, 100 mM ammonium sulfate, 0.1 M 2-(N-morpholino) ethanesulfonic acid pH 6.0. A crystal was soaked by the addition of a 10 mM solution of β-D-arabinofuranosyl cyclitol aziridine-azidooctyl **2** dissolved in water to a final concentration of 3.3 mM in the drop, and fished after 68 hours into liquid nitrogen without additional cryoprotection.

2.16 Data collection and refinement

Data were collected at beamline io4 at the Diamond Light Source (UK), processed using DIALS[12] and scaled with *AIMLESS*[13]. There are 2 molecules per asymmetric unit and the space group is $P2_12_12_1$. The structure was solved using *Phaser*[14] with PDB entry 5BVU as the model, and refined using REFMAC5[15] interspersed with rounds of model building in *Coot*[16]. The ligand was built and dictionary restraints generated using *AceDRG*[17]. The programs were run through the *CCP4I2*[18] graphical interface. Data collection and refinement statistics are given in Table S2.

3 Chemistry

3.1 General

All anhydrous solvents were dried over 4 Å molecular sieves. Reactions were performed under Argon atmosphere. Unless stated otherwise, solvents were removed under reduced pressure at 40 °C. Reactions were monitored by TLC analysis using Merck 25 DC plastikfolien 60 F_{254} with detection by UV (254 nm) or by spraying with molybdene stain ($(NH_4)_6Mo_7O_{24}\cdot 4H_2O$ (25 g/L) and $(NH_4)_4Ce(SO_4)_4\cdot 2H_2O$ in 10% sulfuric acid) followed by charring at approx. 150 °C. Column chromatography was performed manually using either Baker or Screening Device silica gel 60 (0.04-0.063 mm) or a Biotage Isolera™ flash purification system using silica gel cartridges (Screening Device SilicaSep HP, particle size 15-40 µm, 60A) in the indicated solvents. High resolution mass spectra were recorded by direct injection (2 µL of a 2 µM solution in water/acetonitrile; 50/50; v/v and 0.1% formic acid) on a mass spectrometer (Thermo Finnigan LTQ Orbitrap) equipped with an electrospray ion source in positive mode with resolution R = 60000 at m/z 400 (mass range m/z = 150-2000) and dioctylpthalate (m/z = 391.2842) as a "lock mass". The high-resolution mass spectrometer was calibrated prior to measurements with a calibration mixture (Thermo Finnigan). LC-MS analysis was performed on a LCQ Advantage Max (Thermo Finnigan) ion-trap spectrometer (ESI+) coupled to a Surveyor HPLC system (Thermo Finnigan) equipped with a C18 column (Gemini, 4.6 mm x 50 mm, 3 µM particle size, Phenomenex) equipped with buffers A: H_2O, B: acetonitrile (MeCN), C: 1% TFA; or an Agilent technologies 1260 infinity LC-MS with a 6120 Quadrupole MS system equipped with buffers A: H_2O, B: acetonitrile (MeCN) and C: 100 mM NH_4OAc. ^{1}H-NMR and ^{13}C-NMR spectra were recorded on Bruker AV-400 or Bruker AV-600. ^{13}C-NMR spectra are Attached Proton Test (APT) spectra with phase inverted (180°). Chemical shifts were given in ppm (δ) relative to MeOD-d_4 (3.31 ppm, ^{1}H-NMR and 49.00 ppm, ^{13}C-NMR) and coupling constants are given in Hz. The following abbreviations are used to describe peak patterns when appropriate: s (singlet), d (doublet), t (triplet), q (quartet), quint (quintet), m (multiplet), Ar (aromatic), C_q (quarternary carbon). 2D NMR experiments (HSQC, COSY) were carried out to assign protons and carbons of the new structures and numbering and assignation follows the general numbering shown in compound **2** (see below).

Compounds used in this work were synthesized at the Bio-organic Synthesis Department, Leiden Institute of Chemistry at Leiden University, according to published methods: β-D-arabinofuranosyl cyclitol-aziridine **1,**[19] α-L-arabinofuranosyl cyclitol-configured ABPs **12**,[20] **13**,[20] and **14**,[20] β-L-arabinofuranosyl cyclitol-configured compounds **15**[21] and **16-20**,[22] cyclophellitol-configured probes **7**[23], **8**[24], **9**[25], **10**[8] and **11**[26] and inhibitors **21**[8] and **22**[9] were synthesized according to the described procedures. New β-L-arabinofuranosyl ABPs **2**-**6** were synthesized as described below.

3.2 Synthesis of β-D-arabinofuranosyl cyclitol aziridine ABPs

Starting from β-D-arabinofuranosyl cyclitol aziridine **1** that was prepared according to the literature[19], we employed standard conditions for aziridine *N*-alkylation to obtain *N*-azidooctyl β-D-arabinofuranosyl cyclitol aziridine **2**, enabling further functionalization using click chemistry using fluorescent- and biotin-containing alkynes that were prepared according to the literature (Scheme S1).[27]

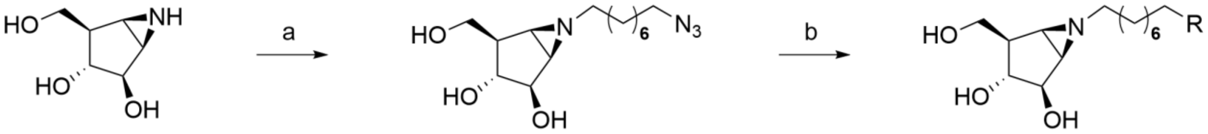

1 **2**

3, R = BODIPY-green
4, R = BODIPY-red
5, R = Cy5
6, R = biotin

Scheme S1. Synthesis of ABPs. Reagents and conditions: a) 1-azido-8-iodooctane, K_2CO_3, DMF; 31% yield; b) alkyne, $CuSO_4$, sodium ascorbate, DMF; 61% yield for **3**; 42% yield for **4**; 22% yield for **5**; 40% yield for **6**.

3.3 Experimental procedures and characterization data

N-azidooctyl β-D-arabinofuranosyl cyclitol aziridine (2)

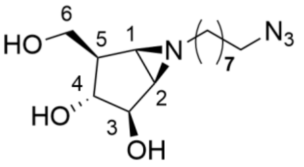

A flask was charged with β-D-arabinofuranosyl cyclitol aziridine (**1**) (82 mg, 0.56 mmol) and anhydrous DMF (2.5 mL). 1-azido-8-iodooctane[28] (321 mg, 1.14 mmol) and potassium carbonate (235 mg, 1.70 mmol) were added and the mixture was heated to 80 °C and stirred overnight. The mixture was then concentrated to dryness and the product was purified by neutralized silica gel chromatography (0% → 10% MeOH in CH_2Cl_2) to afford the product as a fluffy powder in 31% yield (51.7 mg, 0.17 mmol). ^{1}H-NMR (MeOD, 400 MHz) : *δ* 3.90 (dd, *J* = 6.3, 3.0 Hz, 1H, H–3), 3.76 (dd, *J* = 10.2, 4.5 Hz, 1H, H–6a), 3.66 (t, *J* = 10.0 Hz, 1H, H–6b), 3.28 (t, *J* = 6.8 Hz, 2H, CH_2–N_3), 3.22 – 3.14 (m, 1H, H–4), 2.30 (dt, *J* = 11.7, 7.6 Hz, 1H, C*H*HN), 2.16 (dd, *J* = 5.3, 2.7 Hz, 1H, H–1), 2.12 (dd, *J* = 5.3, 3.0 Hz, 1H, H–2), 1.99 (dt, *J* = 11.7, 7.3 Hz, 1H, CH*H*N), 1.92 (ddd, *J* = 9.8, 7.4, 4.2 Hz, 1H, H–5), 1.63 – 1.52 (m, 4H, C*H*$_2$CH$_2$N, C*H*$_2$CH$_2$N$_3$), 1.46 – 1.22 (m, 8H, 4xCH_2). ^{13}C-NMR (MeOD, 101 MHz) : *δ* 80.2 (C–3) , 77.3 (C–4), 62.6 (C–6), 59.7 (CH_2N), 52.4 (CH_2N_3), 49.1 (C–5), 46.2 (C–2), 43.0 (C–1), 30.53, 30.49, 30.2, 29.9, 28.2, 27.7 (6xCH_2). HRMS: calcd. for $[C_{14}H_{26}N_4O_3 + H]^+$ 299.2078, found 299.2078.

β-D-Arabinofuranosyl cyclitol aziridine BODIPY-red-tagged ABP (3)

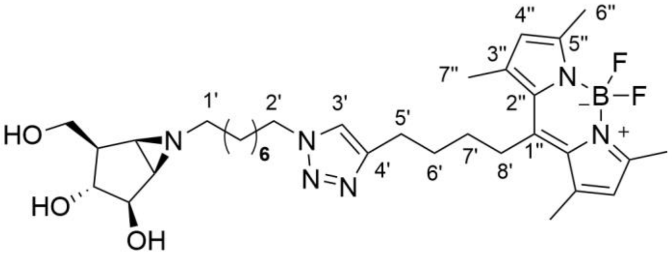

A flask was charged with *N*-azidooctyl β-D-arabinofuranosyl cyclitol aziridine (**2**) (5.1 mg, 17 µmol) and DMF (0.7 mL), BODIPY-green alkyne[29] (9.9 mg, 34 µmol), $CuSO_4$ (33 µL of 100 mM solution in H_2O) and

sodium ascorbate (50 µL of 100 mM solution in H_2O) were added and the solution was stirred overnight at room temperature. The volatiles were removed under reduced pressure and the product was purified by neutralized silica gel chromatography (0% → 10% MeOH in CH_2Cl_2) followed by semi-preparative reversed phase HPLC (linear gradient: 42% → 48%, solutions used: A: 50 mM NH_4HCO_3 in H_2O, B: acetonitrile). Freeze drying yielded the product as a yellow powder in 61% yield (6.0 mg, 10 µmol). ^{1}H-NMR (MeOD, 600 MHz) : δ 7.74 (s, 1H, H–3'), 6.12 (s, 2H, 2xH–4''), 4.35 (t, J = 7.0 Hz, 2H, CH_2–2'), 3.88 (dd, J = 6.3, 3.0 Hz, 1H, H–3), 3.76 (dd, J = 10.2, 4.4 Hz, 1H, H–6a), 3.65 (t, J = 10.0 Hz, 1H, H–6b), 3.17 (dd, J = 8.0, 6.3 Hz, 1H, H–4), 3.06 – 3.00 (m, 2H, CH_2–8'), 2.79 (t, J = 7.2 Hz, 2H, CH_2–5'), 2.44 (s, 6H, 2xCH_3–6'' or 2xCH_3–7''), 2.39 (s, 6H, 2xCH_3–6'' or 2xCH_3–7''), 2.25 (dt, J = 11.7, 7.2 Hz, 1H, H–1'a), 2.13 (dd, J = 5.2, 2.8 Hz, 1H, H–1), 2.08 (dd, J = 5.2, 3.1 Hz, 1H, H–2), 1.99 – 1.82 (m, 6H, H–5, H–1'b, CH_2–6', CH_2), 1.70 – 1.61 (m, 2H, CH_2–7'), 1.52 (quint, J = 7.4, 6.3 Hz, 2H, CH_2), 1.37 – 1.22 (m, 8H, 4xCH_2). ^{13}C-NMR (MeOD, 151 MHz) : δ 154.9, 148.5, 147.9, 142.2, 132.6 (5xC_q Ar), 123.4 (C–3'), 122.7 (C–4''), 80.2 (C–3), 77.4 (C–4), 62.6 (C–6), 59.7 (C–1'), 51.2 (C–2'), 46.2 (C–2), 43.0 (C–1), 32.4 (C–7'), 31.2 (CH_2), 30.8 (C–6'), 30.49, 30.40, 29.9 (3xCH_2), 29.1 (C–8'), 28.1, 27.3 (2xCH_2), 25.9 (C–5'), 16.5, 14.4 (C–6'', C–7''). HRMS: calcd. for $[C_{33}H_{49}N_6O_3BF_2 + H]^+$ 627.4000, found 627.4009. **Note:** The ^{13}C-NMR signal of C–5 overlaps with the MeOD multiplet and is therefore unassigned.

β-D-Arabinofuranosyl cyclitol aziridine BODIPY-red-tagged ABP (4)

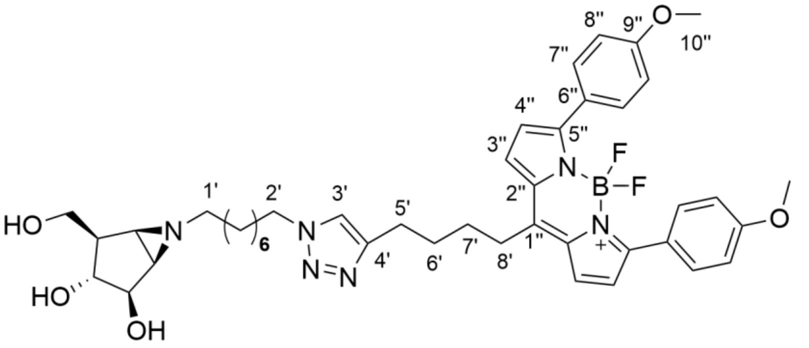

A flask was charged with *N*-azidooctyl β-D-arabinofuranosyl cyclitol aziridine (**2**) (4.5 mg, 15 µmol) and anhydrous DMF (0.7 mL), BODIPY-red alkyne[29] (16.2 mg, 34 µmol), $CuSO_4$ (33 µL of 100 mM solution in H_2O) and sodium ascorbate (50 µL of 100 mM solution in H_2O) were added and the solution was stirred overnight at room temperature. The volatiles were removed under reduced pressure and the product was purified by semi-preparative reversed phase HPLC (linear gradient: 49% → 55%, solutions used: A: 50 mM NH_4HCO_3 in H_2O, B: acetonitrile). Freeze drying yielded the product as a dark blue powder in 42% yield (4.9 mg, 6.3 µmol). ^{1}H-NMR (MeOD, 600 MHz) : δ 7.84 (d, J = 8.9 Hz, 4H, 4xH–7''), 7.69 (s, 1H, H–3'), 7.43 (d, J = 4.3 Hz, 2H, 2xH–3''), 6.97 (d, J = 8.9 Hz, 4H, 4xH–8''), 6.69 (d, J = 4.3 Hz, 2H, 2xH–4''), 4.32 (t, J = 7.0 Hz, 2H, CH_2–2'), 3.87 (dd, J = 6.2, 3.0 Hz, 1H, H–3), 3.85 (s, 6H, 2xCH_3–10''), 3.75 (dd, J = 10.3, 4.4 Hz, 1H, H–6a), 3.64 (t, J = 10.0 Hz, 1H, H–6b), 3.16 (dd, J = 8.0, 6.3 Hz, 1H, H–4), 3.06 (t, J = 7.2 Hz, 2H, CH_2–8'), 2.78 (t, J = 6.8 Hz, 2H, CH_2–5'), 2.22 (dt, J = 11.7, 7.3 Hz, 1H, H–1'a), 2.10 (dd, J = 5.2, 2.8 Hz, 1H, H–1), 2.06 (dd, J = 5.2, 3.1 Hz, 1H, H–2), 1.96 – 1.80 (m, 8H, H–5, H–1'b, CH_2–6', CH_2–7', CH_2), 1.49 (quint, J = 7.4 Hz, 2H, CH_2), 1.32 – 1.18 (m, 8H, 4xCH_2). ^{13}C-NMR (MeOD, 151 MHz) : δ 162.2, 158.8, 148.6, 146.8, 137.5 (5xC_q Ar), 132.2 (t, J = 4.4 Hz, C–7''), 128.4 (C–3''), 126.5 (C_q Ar), 123.3 (C–3'), 121.0 (C–4''), 114.6 (C–8''), 80.2 (C–3), 77.4 (C–4), 62.7 (C–6), 59.6 (C–1'), 55.8 (C–10''), 51.2 (C–2'), 46.2 (C–2), 43.0 (C–1), 34.1, 31.2 (2xCH_2), 31.0 (C–8'), 30.48, 30.40, 30.32, 29.9, 28.2, 27.3 (6xCH_2), 25.8 (C–5'). HRMS: calcd. for $[C_{43}H_{53}N_6O_5BF_2 + H]^+$ 783.4211, found 783.4221. **Note:** The ^{13}C-NMR signal of C–5 overlaps with the MeOD multiplet and is therefore unassigned.

β-D-Arabinofuranosyl cyclitol aziridine Cy5-tagged ABP (5)

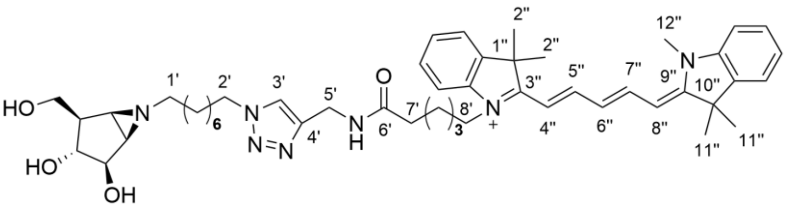

A flask was charged with *N*-azidooctyl β-D-arabinofuranosyl cyclitol aziridine (**2**) (5.1 mg, 17 µmol) and DMF (0.7 mL), Cy5-alkyne (18.1 mg, 32 µmol), $CuSO_4$ (33 µL of 100 mM solution in H_2O) and sodium ascorbate (50 µL of 100 mM solution in H_2O) were added and the solution was stirred overnight at room temperature. The volatiles were removed under reduced pressure and the product was purified by semipreparative reversed phase HPLC (linear gradient: 45% → 51%, solutions used: A: 50 mM NH_4HCO_3 in H_2O, B: acetonitrile). Freeze drying yielded the product as a blue powder in 22% yield (3.19 mg, 3.7 µmol). ^{1}H-NMR (MeOD, 600 MHz) : δ 8.25 (td, *J* = 13.9, 13.3, 2.7 Hz, 2H, H–5'', H–7''), 7.84 (s, 1H, H–3'), 7.50 (dd, *J* = 7.5, 1.1 Hz, 2H, 2xCH Ar), 7.42 (tdd, *J* = 7.5, 6.1, 1.2 Hz, 2H, 2xCH Ar), 7.33 – 7.24 (m, 4H, 4xCH Ar), 6.62 (t, *J* = 12.4 Hz, 1H, H–6''), 6.28 (dd, *J* = 13.8, 2.6 Hz, 2H, H–4'', H–8''), 4.40 (s, 2H, CH_2–5'), 4.36 (t, *J* = 7.1 Hz, 2H, CH_2–2'), 4.09 (t, *J* = 7.5 Hz, 2H, CH_2–8'), 3.88 (dd, *J* = 6.3, 3.0 Hz, 1H, H–3), 3.76 (dd, *J* = 10.2, 4.4 Hz, 1H, H–6a), 3.67 – 3.71 (m, 4H, H–6b, CH_3–12''), 3.16 (dd, *J* = 8.0, 6.2 Hz, 1H, H–4), 2.30 – 2.22 (m, 3H, H–1'a, CH_2–7'), 2.13 (dd, *J* = 5.2, 2.8 Hz, 1H, H–1), 2.09 (dd, *J* = 5.2, 3.0 Hz, 1H, H–2), 1.96 (dt, *J* = 11.8, 7.0 Hz, 1H, H–1'b), 1.92 – 1.78 (m, 5H, H–5, 2xCH_2), 1.75 – 1.68 (m, 14H, 2xCH_3–2'', 2xCH_3–11'', CH_2), 1.56 – 1.43 (m, 4H, 2xCH_2), 1.37 – 1.24 (m, 8H, 4xCH_2). ^{13}C-NMR (MeOD, 151 MHz) : δ 175.8, 175.4, 174.6 (C–6', C–3'', C–9''), 155.56, 155.48 (C–5'', C–7''), 146.1, 144.2, 143.5, 142.6, 142.5 (C–4', 4xC_q Ar), 129.79, 129.75 (2xCH Ar), 126.6 (C–6''), 126.29, 126.23 (2xCH Ar), 124.2 (C–3'), 123.42, 123.29 (2xCH Ar), 112.0, 111.9 (2xCH Ar), 104.4, 104.2 (C–4'', C–8''), 80.2 (C–3), 77.4 (C–4), 62.7 (C–6), 59.7 (C–1'), 51.3 (C–2'), 50.54, 50.51 (C–1'', C–10''), 46.2 (C–2), 44.8 (C–8'), 43.0 (C–1), 36.5 (C–7'), 35.6 (C–5'), 31.5 (C–12''), 31.3, 30.51, 30.44, 29.9, 28.19, 28.12 (6xCH_2), 27.94, 27.79 (C–2'', C–11''), 27.38, 27.30, 26.4 (3xCH_2). HRMS: calcd. for $[C_{49}H_{68}N_7O_4]^+$ 818.5327, found 818.5349 **Note:** The ^{13}C-NMR signal of C–5 overlaps with the MeOD multiplet and is therefore unassigned.

β-D-Arabinofuranosyl cyclitol aziridine biotin-tagged ABP (6)

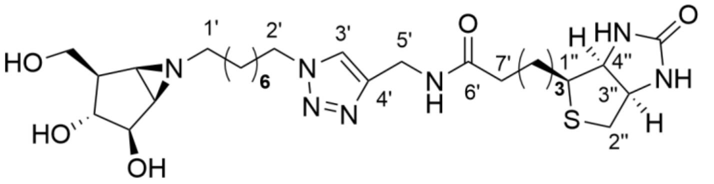

A flask was charged with *N*-azidooctyl β-D-arabinofuranosyl cyclitol aziridine (**2**) (6.2 mg, 21 µmol) and DMF (0.9 mL), Biotin-alkyne (7.0 mg, 25 µmol), $CuSO_4$ (17 µL of 1 M solution in H_2O) and sodium ascorbate (18 µL of 1 M solution in H_2O) were added and the solution was stirred overnight at room temperature. The volatiles were removed under reduced pressure and the product was purified by semi-preparative reversed phase HPLC (linear gradient: 19% → 25%, solutions used: A: 50 mM NH_4HCO_3 in H_2O, B: acetonitrile). Freeze drying yielded the product as a white powder in 40% yield (4.83 mg, 8.3 µmol). ^{1}H-NMR (MeOD, 600 MHz) : δ 7.84 (s, 1H, H–3'), 4.50 (dd, *J* = 7.9, 4.8 Hz, 1H, H–3''), 4.42 (s, 2H, CH_2–5'), 4.37 (t, *J* = 7.1 Hz, 2H, CH_2–2'), 4.29 (dd, *J* = 7.9, 4.5 Hz, 1H, H–4''), 3.89 (dd, *J* = 6.3, 3.0 Hz, 1H, H–3), 3.76 (dd, *J* = 10.2, 4.4 Hz, 1H, H–6a), 3.65 (t, *J* = 10.0 Hz, 1H, H–6b), 3.24 – 3.14 (m, 2H, H–4, H–1''), 2.93 (dd, *J* = 12.8, 5.0 Hz, 1H, H–2''a), 2.71 (d, *J* = 12.7 Hz, 1H, H–2''b), 2.32 – 2.18 (m, 3H, H–1'a, CH_2–7'), 2.15 (dd, *J* = 5.2, 2.8 Hz, 1H, H–1), 2.11 (dd, *J* = 5.2, 3.0 Hz, 1H, H–2), 1.99 (dt, *J* = 11.7, 6.9 Hz, 1H, H–1'b), 1.95 – 1.84 (m, 3H, H–5, CH_2),

1.77 – 1.49 (m, 6H, 3xCH_2), 1.42 (quint, *J* = 7.7 Hz, 2H, CH_2), 1.38 – 1.25 (m, 8H, 4xCH_2). ^{13}C-NMR (MeOD, 151 MHz) : δ 176.0, 166.1 (2xCO), 146.3 (C_q Ar), 124.2 (CH Ar), 80.2 (C–3), 77.4 (C–4), 63.3 (C–8'), 62.7 (C–6), 61.6 (C–7'), 59.7 (C–1'), 57.0 (C–5'), 51.4 (C–2'), 46.2 (C–2), 43.0 (C–1), 41.1 (C–6'), 36.5 (C–4'), 35.6 (C–3'), 31.3, 30.52, 30.45, 30.0, 29.7, 29.4, 28.2, 27.4, 26.7 (9xCH_2). HRMS: calcd. for $[C_{27}H_{45}N_7O_5S + H]^+$ 580.3276, found 580.3279. **Note:** The ^{13}C-NMR signal of C–5 overlaps with the MeOD multiplet and is therefore unassigned.

3.4 NMR data

^{1}H-NMR spectrum of *N*-azidooctyl β-D-arabinofuranosyl cyclitol aziridine **2**

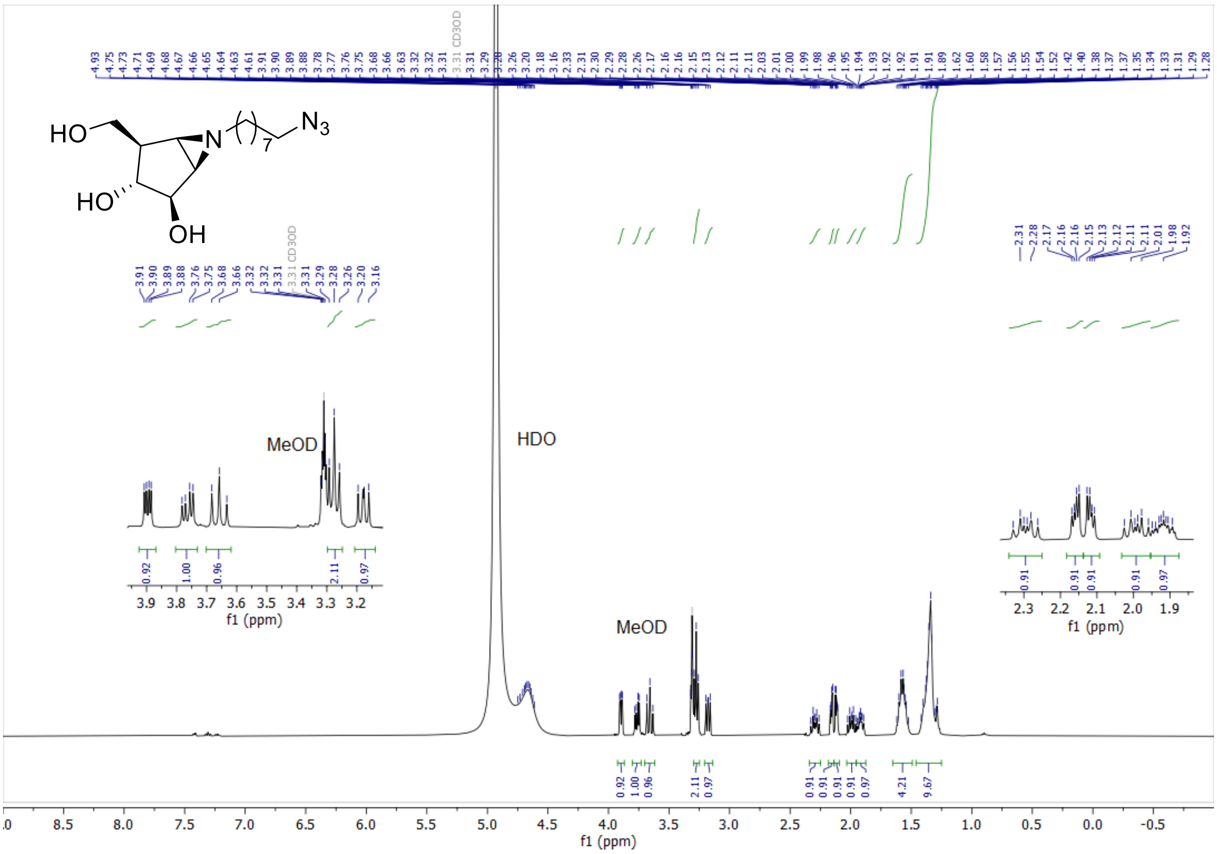

APT-NMR spectrum of *N*-azidooctyl β-D-arabinofuranosyl cyclitol aziridine **2**

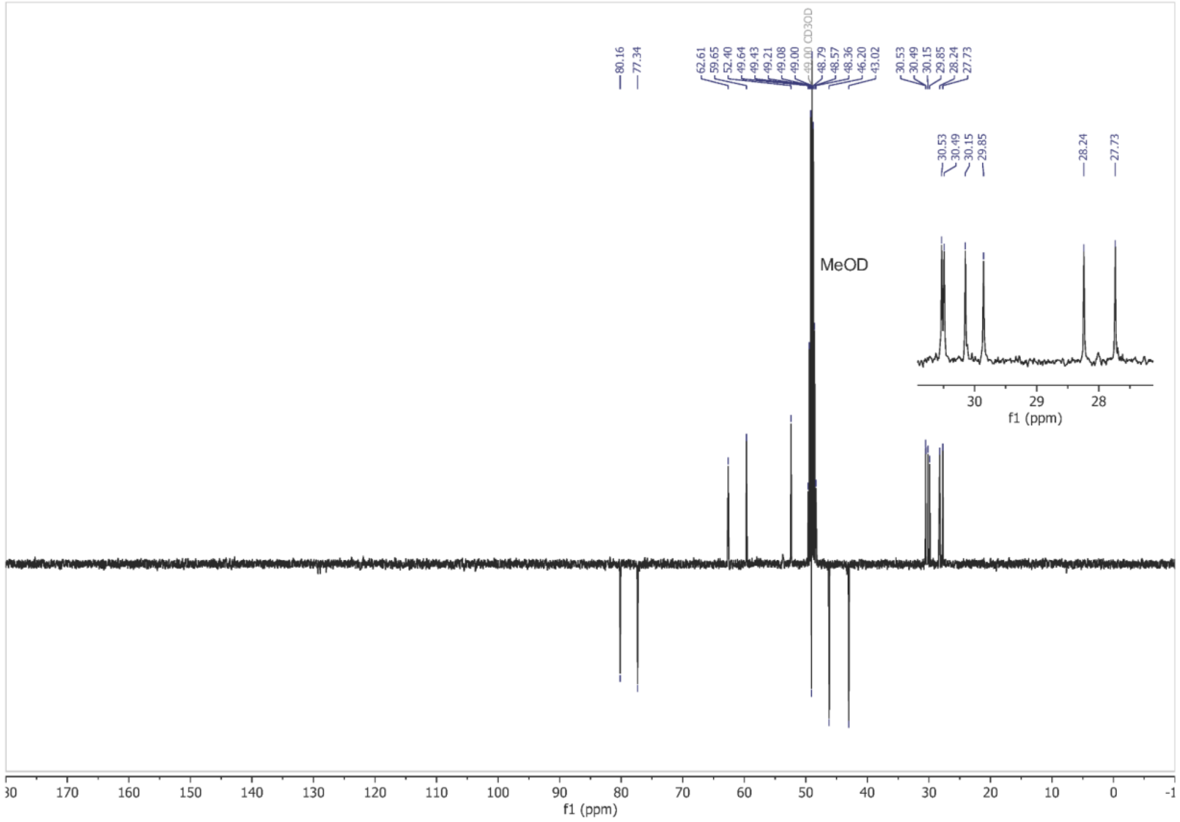

^{1}H-NMR spectrum of β-D-arabinofuranosyl cyclitol aziridine BODIPY-red-tagged ABP **3**

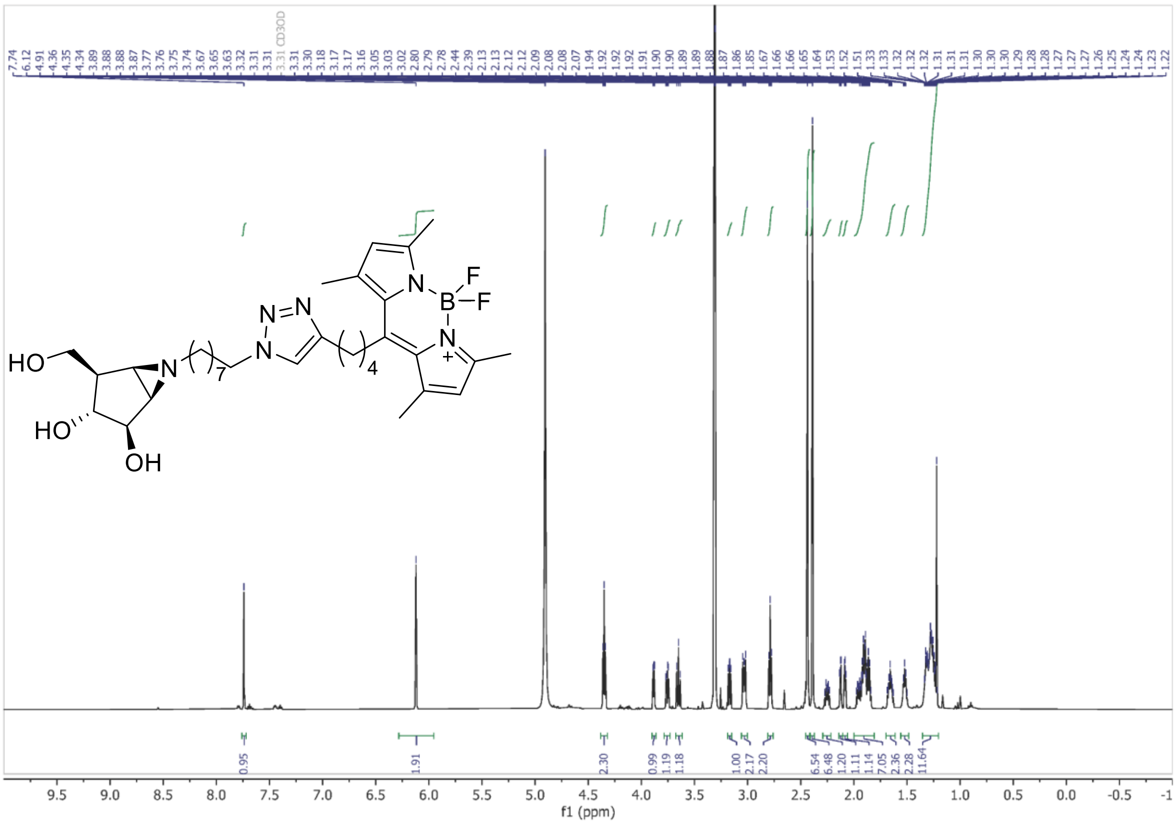

APT-NMR spectrum of β-D-arabinofuranosyl cyclitol aziridine BODIPY-red-tagged ABP **3**

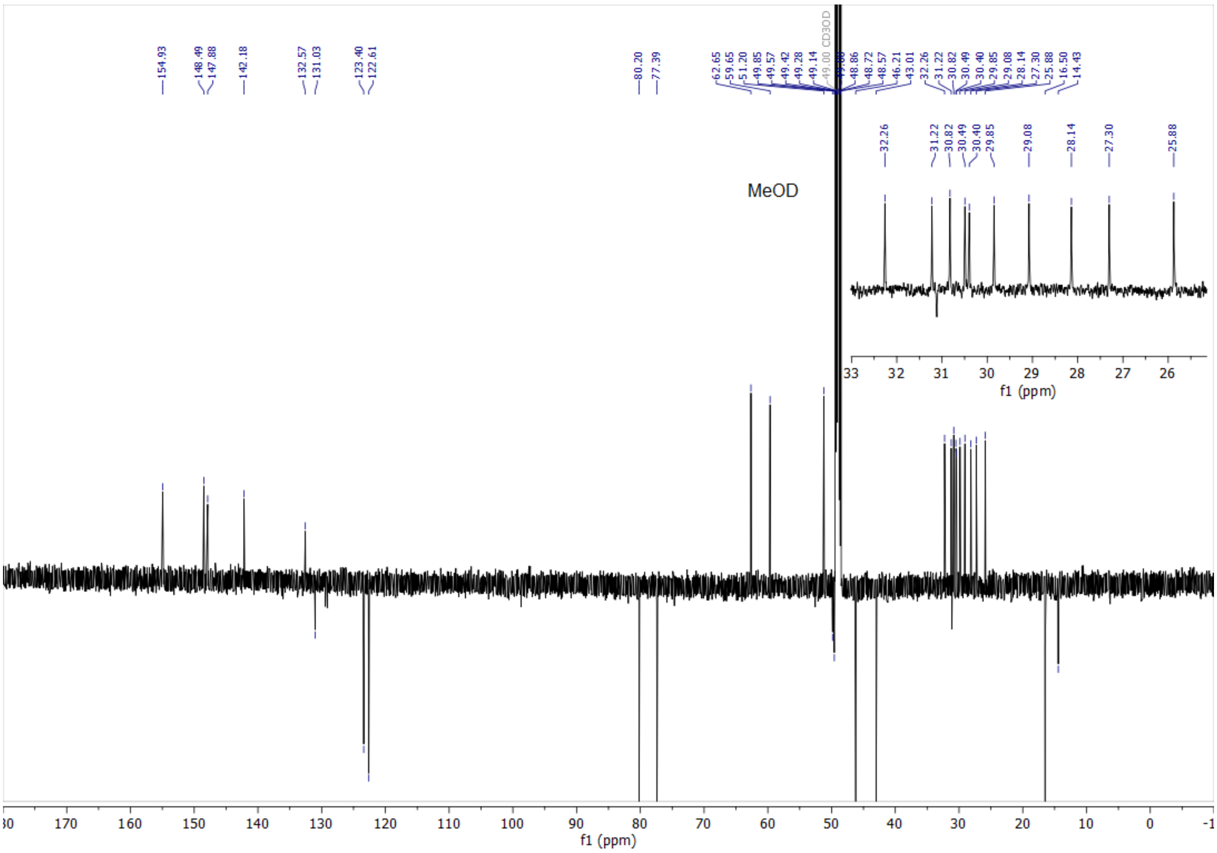

COSY-NMR spectrum of β-D-arabinofuranosyl cyclitol aziridine BODIPY-red-tagged ABP **3**

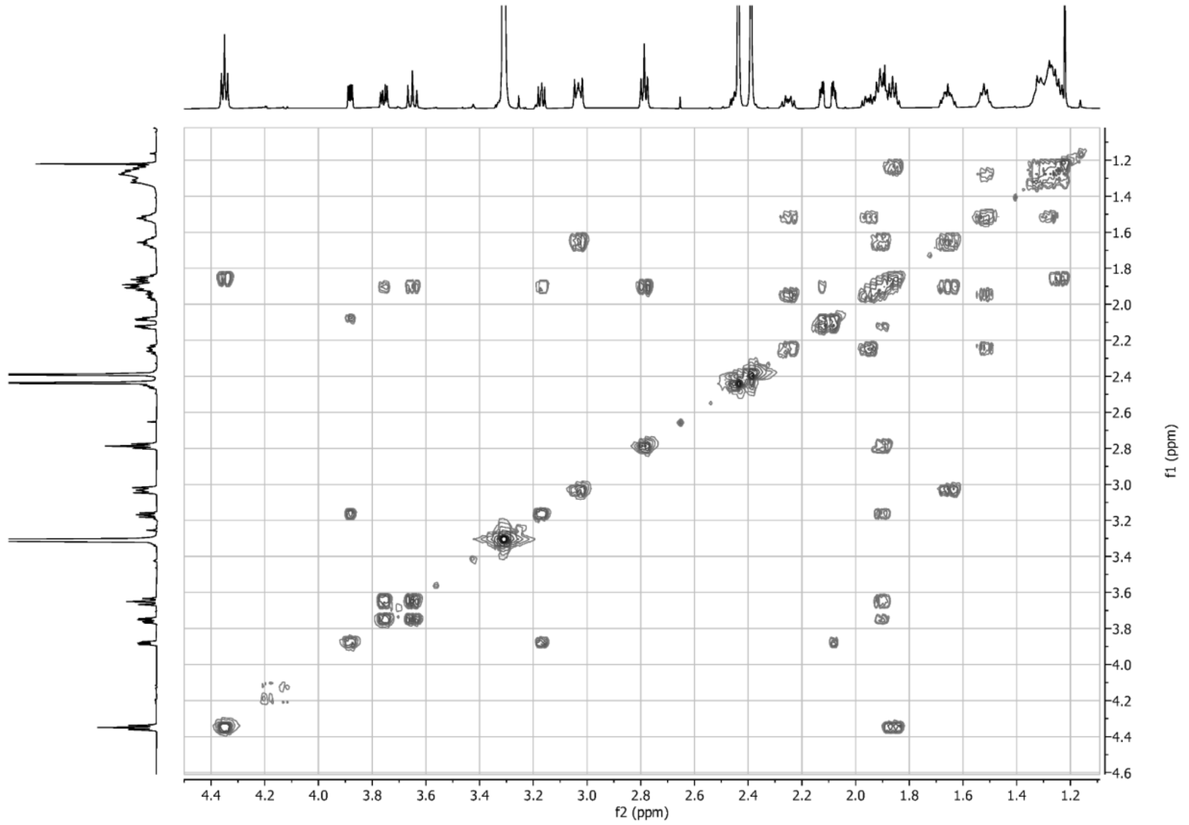

HSQC-NMR spectrum of β-D-arabinofuranosyl cyclitol aziridine BODIPY-red-tagged ABP **3**

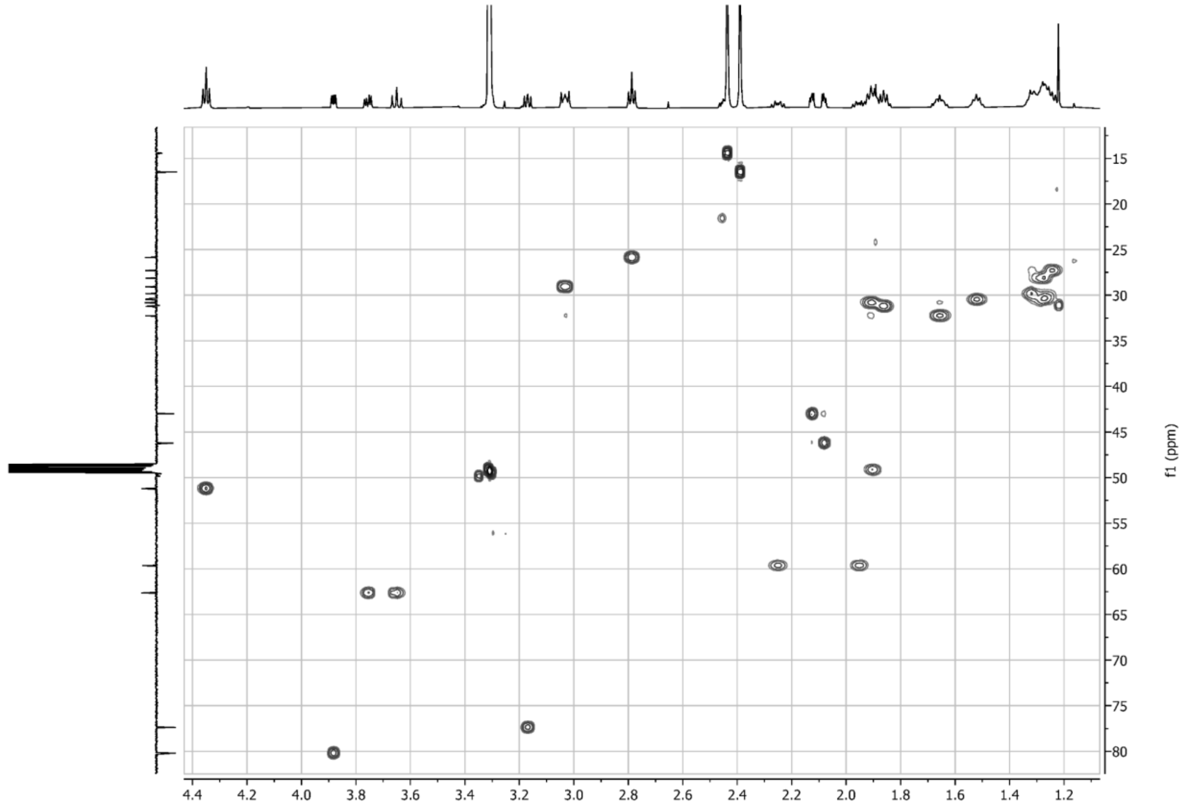

^{1}H-NMR spectrum of β-D-arabinofuranosyl cyclitol aziridine BODIPY-red-tagged ABP **4**

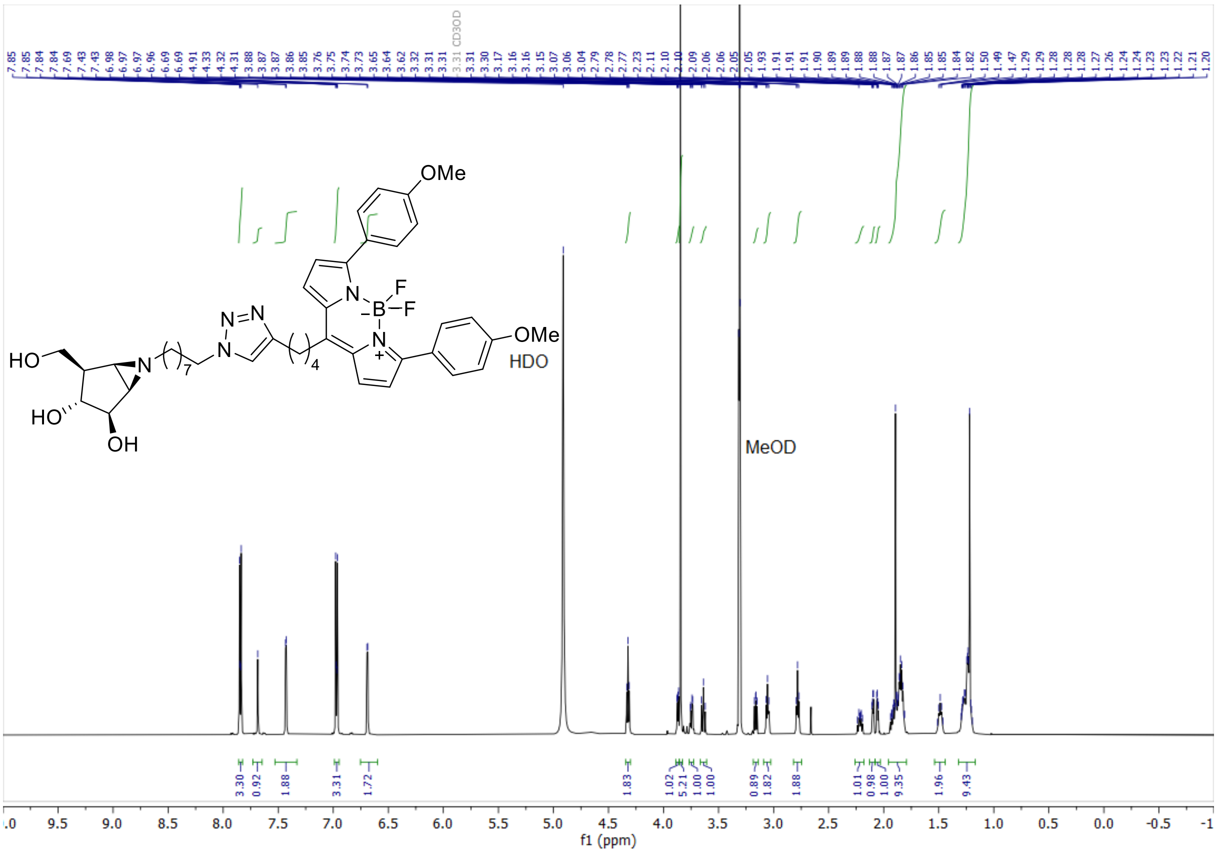

APT-NMR spectrum of β-D-arabinofuranosyl cyclitol aziridine BODIPY-red-tagged ABP **4**

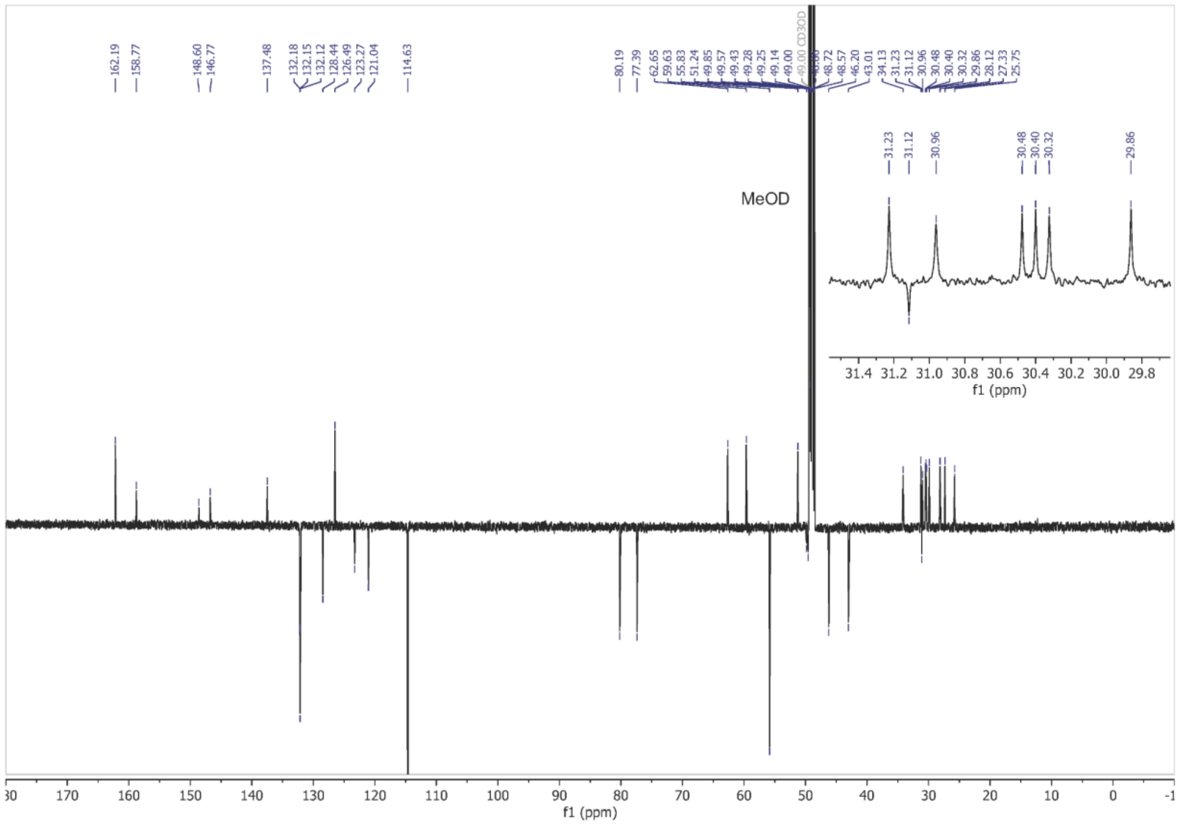

COSY-NMR spectrum of β-D-arabinofuranosyl cyclitol aziridine BODIPY-red-tagged ABP **4**

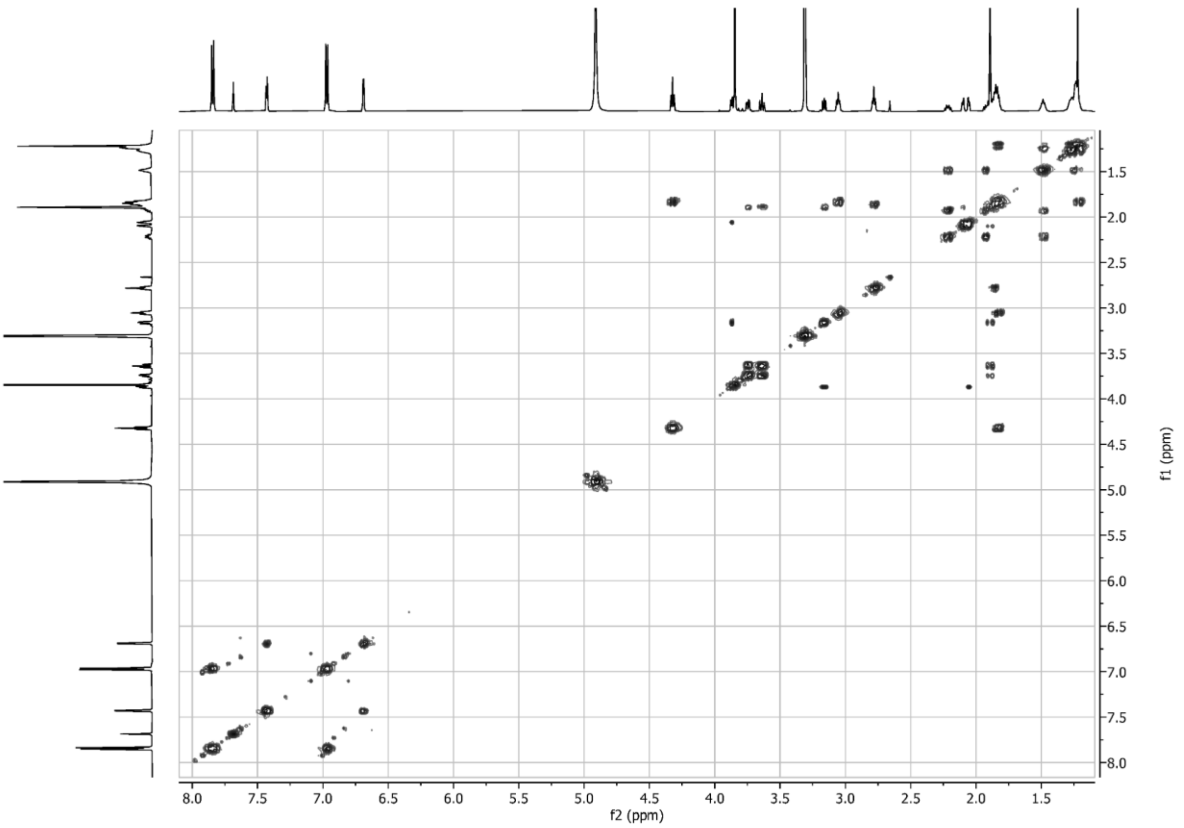

HSQC-NMR spectrum of β-D-arabinofuranosyl cyclitol aziridine BODIPY-red-tagged ABP **4**

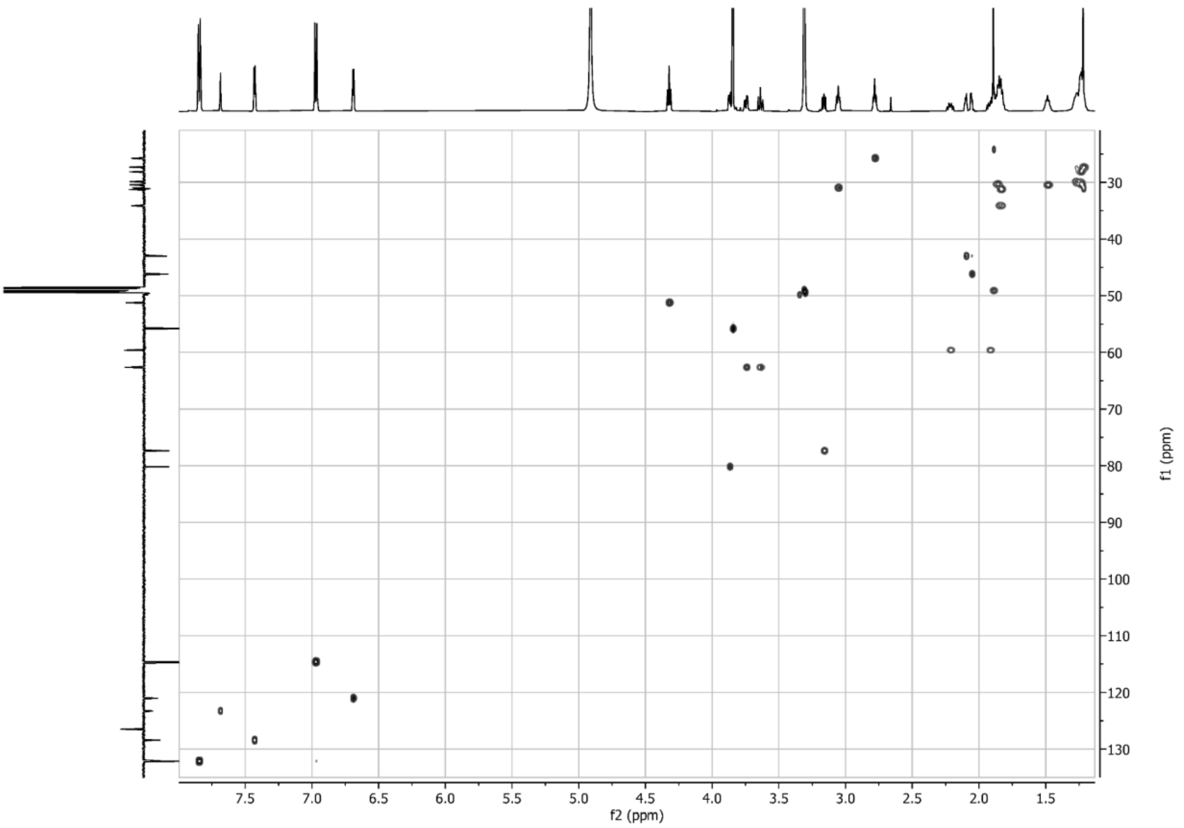

^{1}H-NMR spectrum of β-D-arabinofuranosyl cyclitol aziridine Cy5-tagged ABP **5**

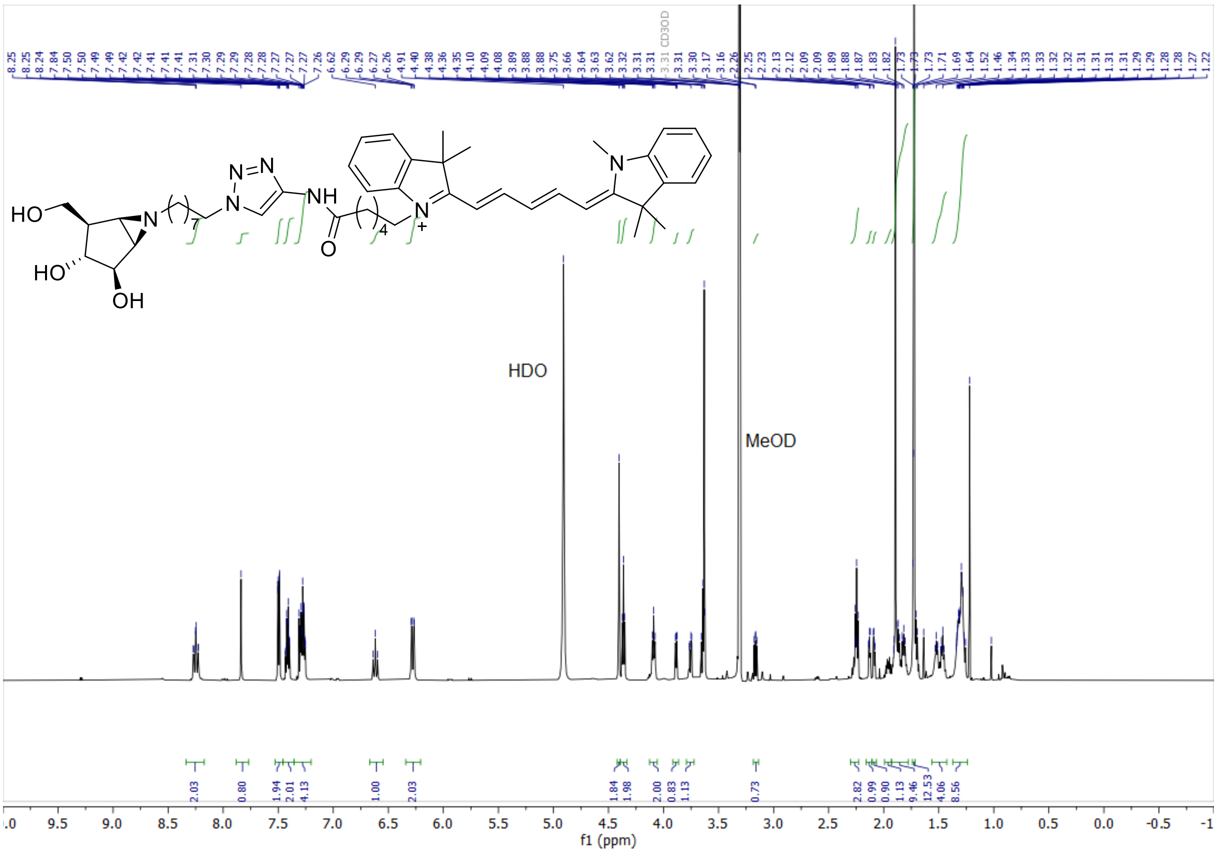

APT-NMR spectrum of β-D-arabinofuranosyl cyclitol aziridine Cy5-tagged ABP **5**

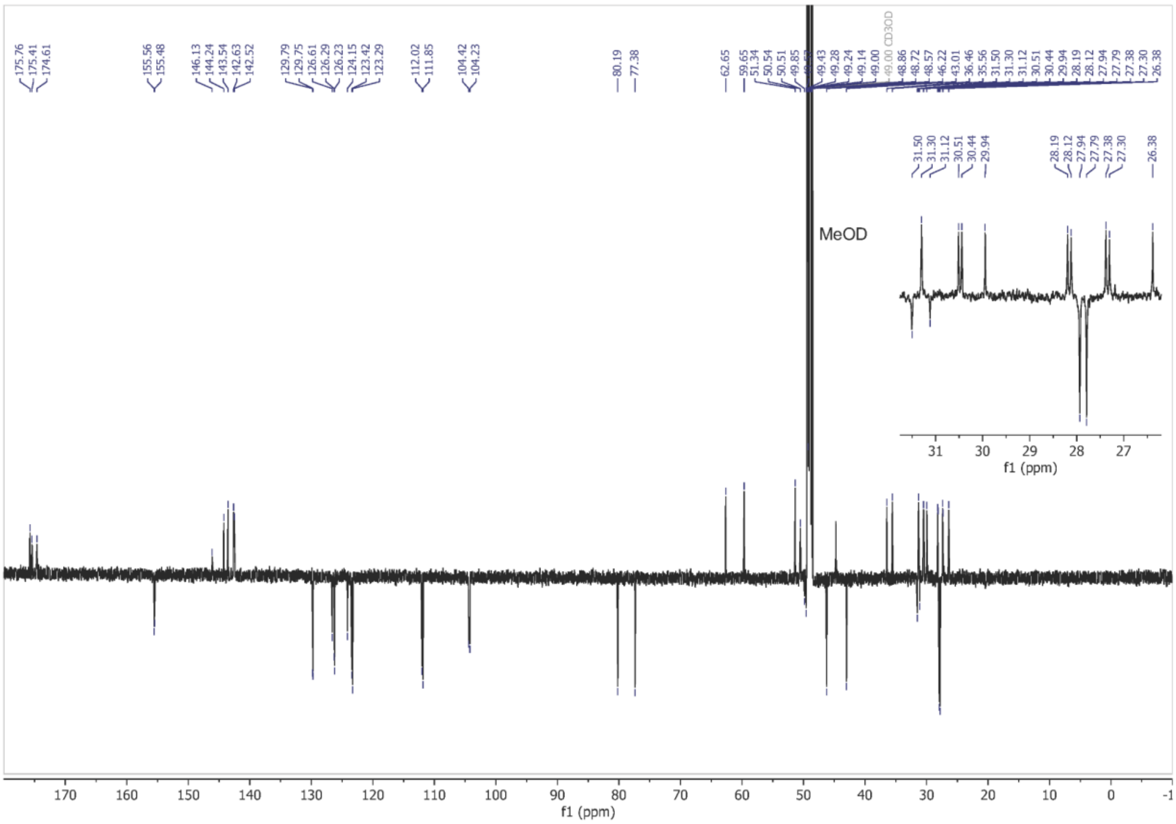

COSY-NMR spectrum of β-D-arabinofuranosyl cyclitol aziridine Cy5-tagged ABP **5**

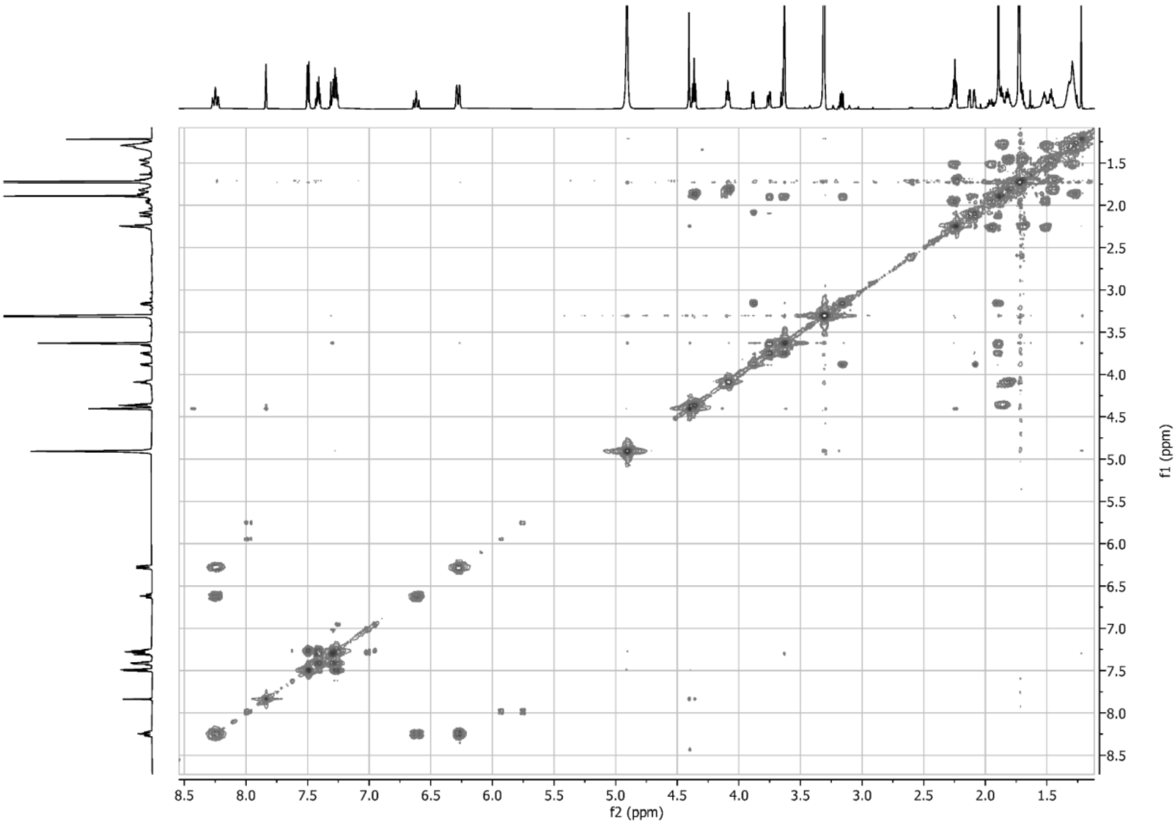

HSQC-NMR spectrum of β-D-arabinofuranosyl cyclitol aziridine Cy5-tagged ABP **5**

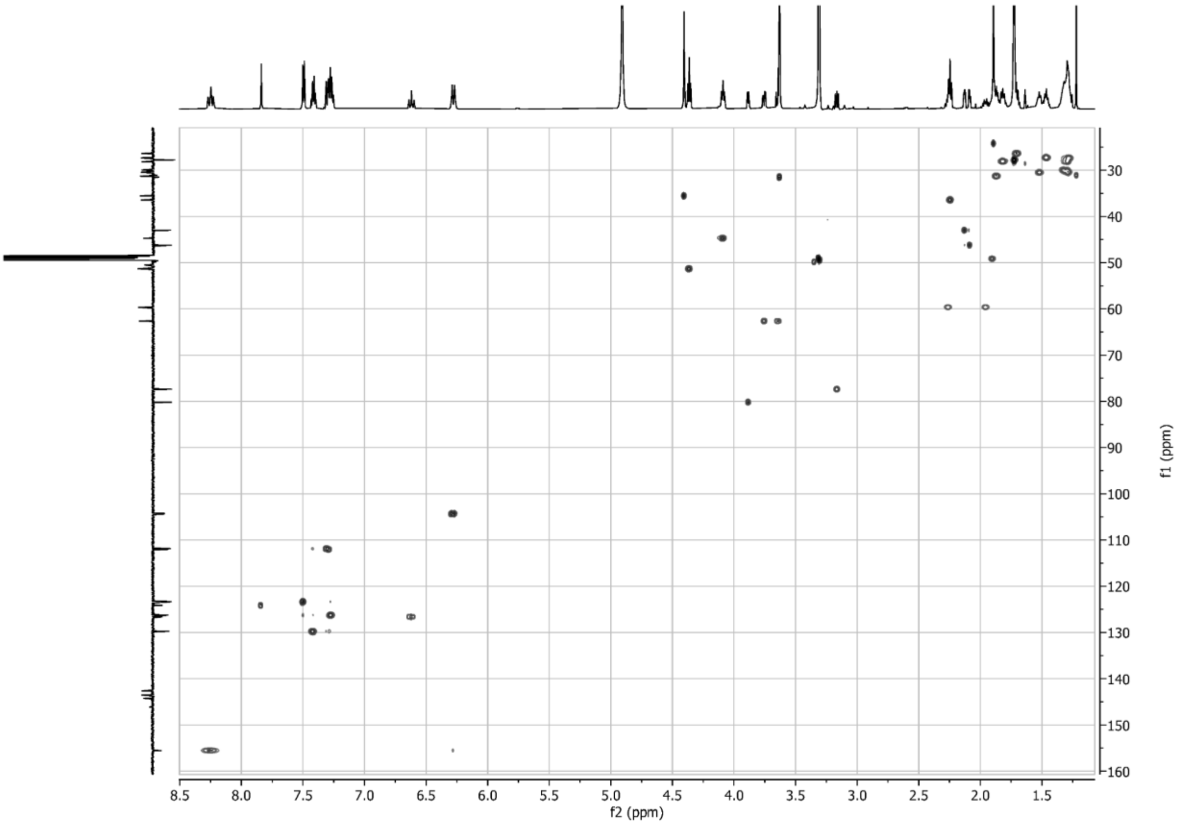

^{1}H-NMR spectrum of β-D-arabinofuranosyl cyclitol aziridine biotin-tagged ABP **6**

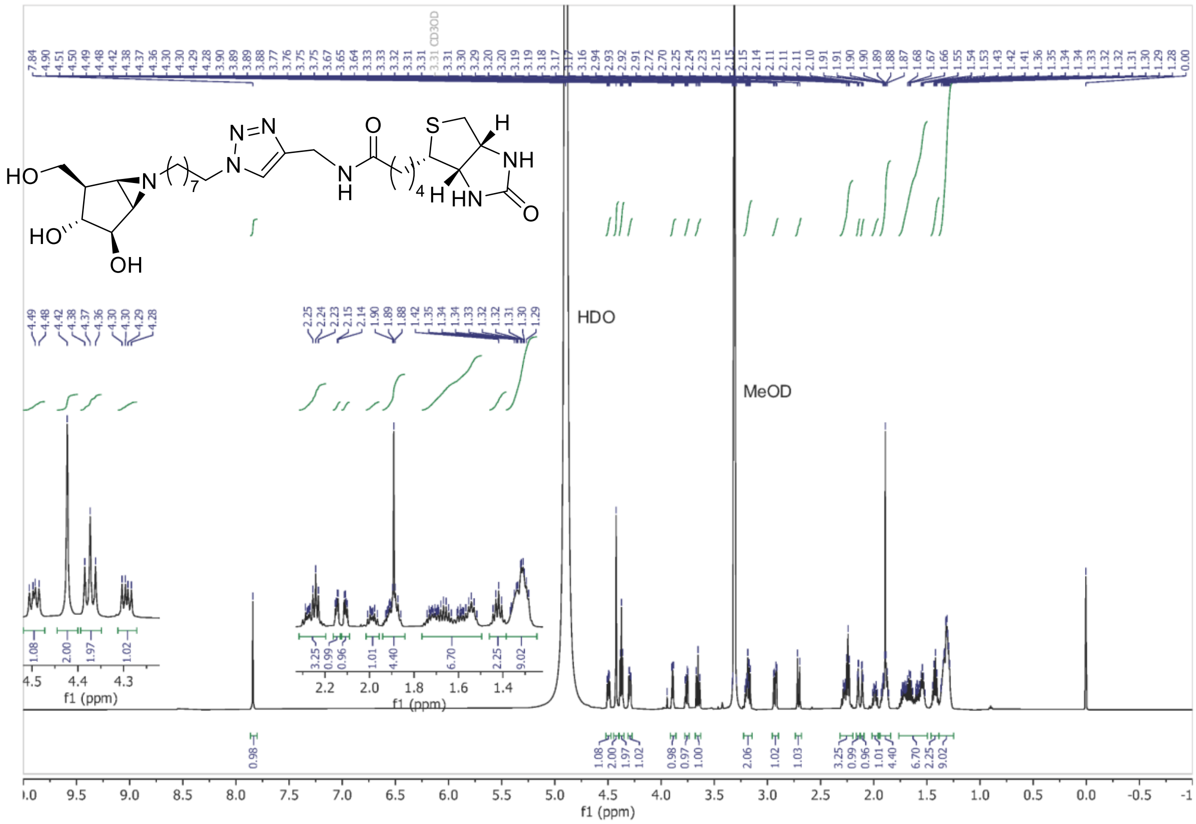

APT-NMR spectrum of β-D-arabinofuranosyl cyclitol aziridine biotin-tagged ABP **6**

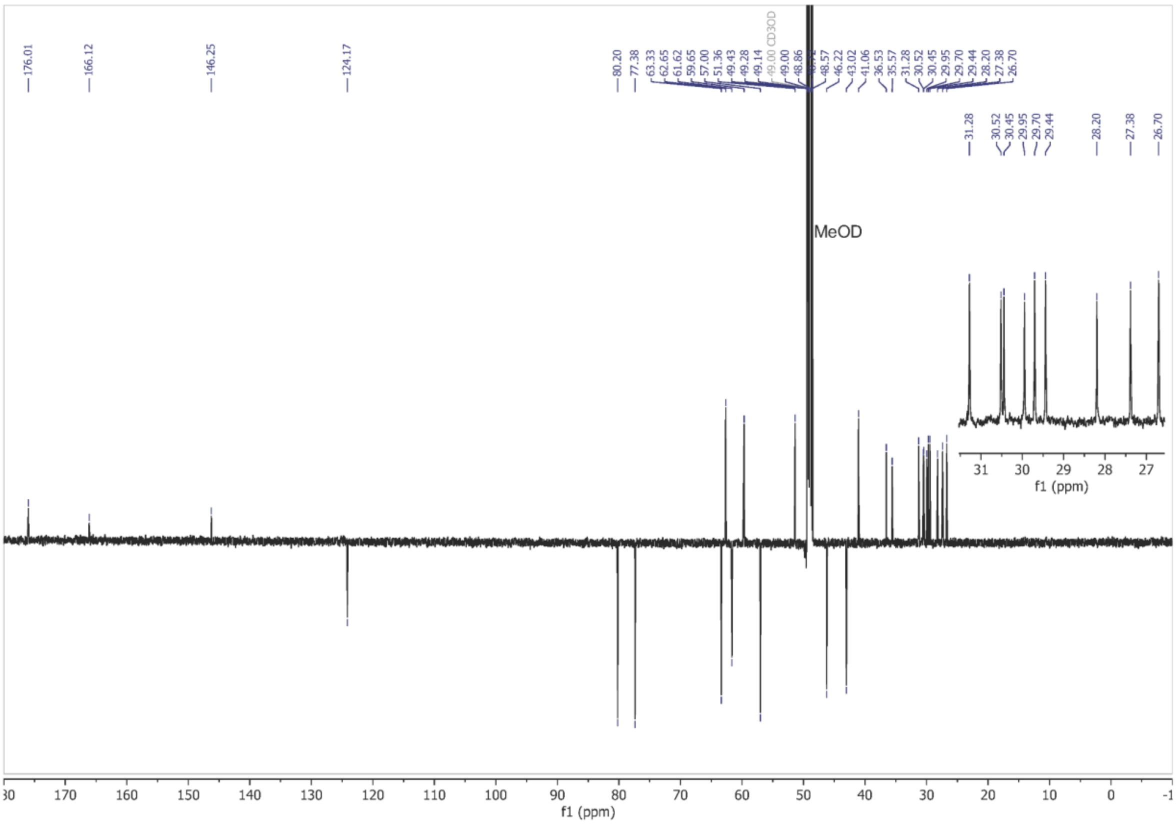

COSY-NMR spectrum of β-D-arabinofuranosyl cyclitol aziridine biotin-tagged ABP **6**

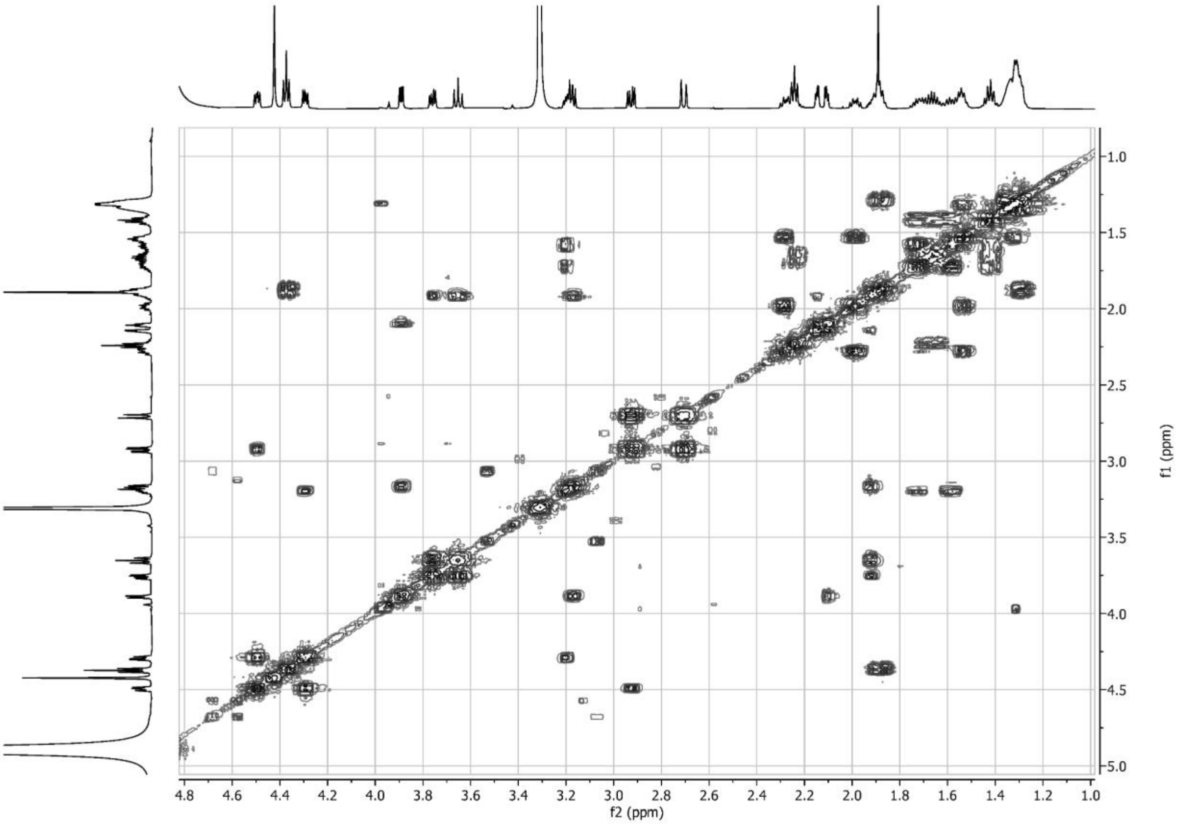

HSQC-NMR spectrum of β-D-arabinofuranosyl cyclitol aziridine biotin-tagged ABP **6**

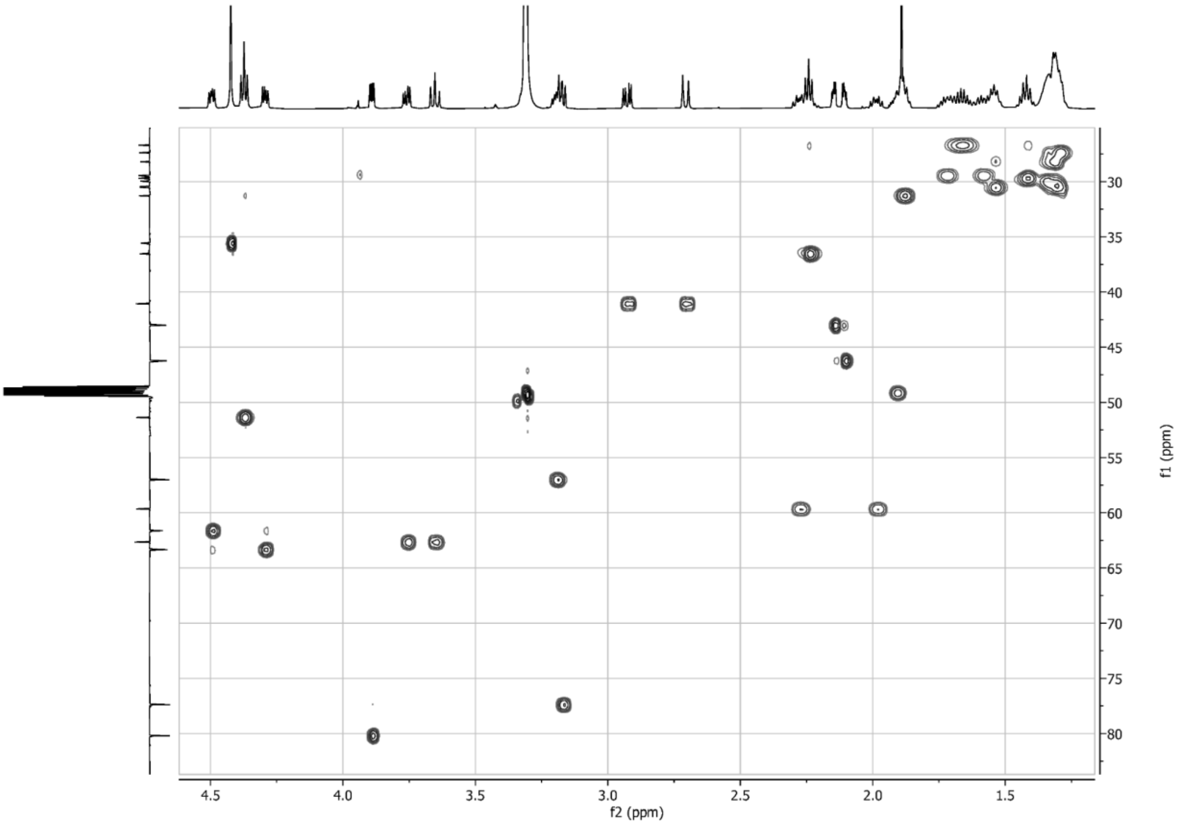

4 Abbreviations

Abbreviations	Full name
4MU-β-D-Glc	4-Methylumbelliferyl-β-D-glucopyranoside
ABP	Activity-based probe
ABPP	Activity-based protein profiling
AMP-DNM	Adamantane-pentyl deoxynojirimycin
Ara*f*	Arabinofuransyl
BCA	Bicinchoninic acid
BLAST	Basic local alignment search tool
CBE	Conduritol B epoxide
CP	Cyclophellitol
DMSO	Dimethyl sulfoxide
GBA	Glucocerebrosidase/ Glucosylceramidase
GBA1/GBA2 KO	Both GBA1 and GBA2 knockout
GBA2/GBA3 OE	Both GBA2 and GBA3 overexpression/overexpressing/overexpressed
GD	Gaucher disease
GlcCer	Glucosylceramide
GlcChol	Glucosyl cholesterol
NaTc	Sodium taurocholate
NBD-GlcCer	Nitrobenzoxadiazole-tagged glucosylceramide
NPC	Niemann Pick disease type C
PBS	Phosphate buffered saline
rhGBA1	Recombinant human GBA1 (Imiglucerase, Cerezyme®)
SDS-PAGE	Sodium dodecyl sulfate–polyacrylamide gel electrophoresis
WB	Western blot

5 References

1. A. R. Marques, M. Mirzaian, H. Akiyama, P. Wisse, M. J. Ferraz, P. Gaspar, K. Ghauharali-van der Vlugt, R. Meijer, P. Giraldo, P. Alfonso, P. Irun, M. Dahl, S. Karlsson, E. V. Pavlova, T. M. Cox, S. Scheij, M. Verhoek, R. Ottenhoff, C. P. van Roomen, N. S. Pannu, M. van Eijk, N. Dekker, R. G. Boot, H. S. Overkleeft, E. Blommaart, Y. Hirabayashi and J. M. Aerts, Glucosylated cholesterol in mammalian cells and tissues: formation and degradation by multiple cellular beta-glucosidases, *J. Lipid. Res.*, 2016, **57**, 451-463.
2. Q. Su, S. P. Schröder, L. T. Lelieveld, M. J. Ferraz, M. Verhoek, R. G. Boot, H. S. Overkleeft, J. M. Aerts, M. Artola and C. L. Kuo, Xylose-configured cyclophellitols as selective inhibitors for glucocerebrosidase, *ChemBioChem*, 2021, **22**, 3090-3098.
3. C. L. Kuo, E. van Meel, K. Kytidou, W. W. Kallemeijn, M. Witte, H. S. Overkleeft, M. Artola and J. M. Aerts, Activity-based probes for glycosidases: profiling and other applications, *Methods Enzymol.*, 2018, **598**, 217-235.
4. C. L. Kuo, W. W. Kallemeijn, L. T. Lelieveld, M. Mirzaian, I. Zoutendijk, A. Vardi, A. H. Futerman, A. H. Meijer, H. P. Spaink, H. S. Overkleeft, J. M. Aerts and M. Artola, In vivo inactivation of glycosidases by conduritol B epoxide and cyclophellitol as revealed by activity-based protein profiling, *FEBS J.*, 2019, **286**, 584-600.
5. W. W. Kallemeijn, M. D. Witte, T. M. Voorn-Brouwer, M. T. Walvoort, K. Y. Li, J. D. Codée, G. A. van der Marel, R. G. Boot, H. S. Overkleeft and J. M. Aerts, A sensitive gel-based method combining distinct cyclophellitol-based probes for the identification of acid/base residues in human retaining beta-glucosidases, *J. Biol. Chem.*, 2014, **289**, 35351-35362.
6. D. Lahav, B. Liu, R. van den Berg, A. van den Nieuwendijk, T. Wennekes, A. T. Ghisaidoobe, I. Breen, M. J. Ferraz, C. L. Kuo, L. Wu, P. P. Geurink, H. Ovaa, G. A. van der Marel, M. van der Stelt, R. G. Boot, G. J. Davies, J. M. Aerts and H. S. Overkleeft, A fluorescence polarization activity-based protein profiling assay in the discovery of potent, selective inhibitors for human nonlysosomal glucosylceramidase, *J. Am. Chem. Soc.*, 2017, **139**, 14192-14197.
7. R. G. Boot, M. Verhoek, W. Donker-Koopman, A. Strijland, J. van Marle, H. S. Overkleeft, T. Wennekes and J. M. Aerts, Identification of the non-lysosomal glucosylceramidase as beta-glucosidase 2, *J. Biol. Chem.*, 2007, **282**, 1305-1312.
8. M. Artola, C. L. Kuo, L. T. Lelieveld, R. J. Rowland, G. A. van der Marel, J. D. C. Codée, R. G. Boot, G. J. Davies, J. M. Aerts and H. S. Overkleeft, Functionalized cyclophellitols are selective glucocerebrosidase inhibitors and induce a bona fide neuropathic Gaucher model in zebrafish, *J. Am. Chem. Soc.*, 2019, **141**, 4214-4218.
9. K. Y. Li, J. Jiang, M. D. Witte, W. W. Kallemeijn, W. E. Donker-Koopman, R. G. Boot, J. M. Aerts, J. D. Codée, G. A. van der Marel and H. S. Overkleeft, Exploring functional cyclophellitol analogues as human retaining beta-glucosidase inhibitors, *Org. Biomol. Chem.*, 2014, **12**, 7786-7791.
10. J. M. Aerts, W. E. Donker-Koopman, G. J. Murray, J. A. Barranger, J. M. Tager and A. W. Schram, A procedure for the rapid purification in high yield of human glucocerebrosidase using immunoaffinity chromatography with monoclonal antibodies, *Anal. Biochem.*, 1986, **154**, 655-663.
11. R. Charoenwattanasatien, S. Pengthaisong, I. Breen, R. Mutoh, S. Sansenya, Y. Hua, A. Tankrathok, L. Wu, C. Songsiririthigul, H. Tanaka, S. J. Williams, G. J. Davies, G. Kurisu and J. R. Cairns, Bacterial beta-glucosidase reveals the structural and functional basis of genetic defects in human glucocerebrosidase 2 (GBA2), *ACS Chem. Biol.*, 2016, **11**, 1891-1900.
12. D. G. Waterman, G. Winter, R. J. Gildea, J. M. Parkhurst, A. S. Brewster, N. K. Sauter and G. Evans, Diffraction-geometry refinement in the DIALS framework, *Acta Crystallogr. D*, 2016, **72**, 558-575.
13. P. R. Evans and G. N. Murshudov, How good are my data and what is the resolution?, *Acta Crystallogr. D*, 2013, **69**, 1204-1214.

14. A. J. McCoy, R. W. Grosse-Kunstleve, P. D. Adams, M. D. Winn, L. C. Storoni and R. J. Read, Phaser crystallographic software, *J. Appl. Crystallogr.*, 2007, **40**, 658-674.
15. G. N. Murshudov, P. Skubak, A. A. Lebedev, N. S. Pannu, R. A. Steiner, R. A. Nicholls, M. D. Winn, F. Long and A. A. Vagin, REFMAC5 for the refinement of macromolecular crystal structures, *Acta Crystallogr. D*, 2011, **67**, 355-367.
16. P. Emsley, B. Lohkamp, W. G. Scott and K. Cowtan, Features and development of Coot, *Acta Crystallogr. D*, 2010, **66**, 486-501.
17. F. Long, R. A. Nicholls, P. Emsley, S. Graaeulis, A. Merkys, A. Vaitkus and G. N. Murshudov, AceDRG: a stereochemical description generator for ligands, *Acta Crystallogr. D*, 2017, **73**, 112-122.
18. L. Potterton, J. Agirre, C. Ballard, K. Cowtan, E. Dodson, P. R. Evans, H. T. Jenkins, R. Keegan, E. Krissinel, K. Stevenson, A. Lebedev, S. J. McNicholas, R. A. Nicholls, M. Noble, N. S. Pannu, C. Roth, G. Sheldrick, P. Skubak, J. Turkenburg, V. Uski, F. von Delft, D. Waterman, K. Wilson, M. Winn and M. Wojdyr, CCP4i2: the new graphical user interface to the CCP4 program suite, *Acta Crystallogr. D*, 2018, **74**, 68-84.
19. O. Lopez Lopez, J. G. Fernandez-Bolanos, V. H. Lillelund and M. Bols, Aziridines as a structural motif to conformational restriction of azasugars, *Org. Biomol. Chem.*, 2003, **1**, 478-482.
20. N. G. S. McGregor, M. Artola, A. Nin-Hill, D. Linzel, M. Haon, J. Reijngoud, A. Ram, M. N. Rosso, G. A. van der Marel, J. D. C. Codée, G. P. van Wezel, J. G. Berrin, C. Rovira, H. S. Overkleeft and G. J. Davies, Rational design of mechanism-based inhibitors and activity-based probes for the identification of retaining alpha-l-arabinofuranosidases, *J. Am. Chem. Soc.*, 2020, **142**, 4648-4662.
21. N. G. S. McGregor, J. Coines, V. Borlandelli, S. Amaki, M. Artola, A. Nin-Hill, D. Linzel, C. Yamada, T. Arakawa, A. Ishiwata, Y. Ito, G. A. van der Marel, J. D. C. Codée, S. Fushinobu, H. S. Overkleeft, C. Rovira and G. J. Davies, Cysteine nucleophiles in glycosidase catalysis: application of a covalent beta-l-arabinofuranosidase inhibitor, *Angew. Chem. Int. Ed.*, 2021, **60**, 5754-5758.
22. V. Borlandelli, W. Offen, O. Moroz, A. Nin-Hill, N. McGregor, L. Binkhorst, A. Ishiwata, Z. Armstrong, M. Artola, C. Rovira, G. J. Davies and H. S. Overkleeft, beta-l-Arabinofurano-cyclitol aziridines are covalent broad-spectrum inhibitors and activity-based probes for retaining beta-l-arabinofuranosidases, *ACS Chem. Biol.*, 2023, **18**, 2564-2573.
23. S. P. Schröder, J. W. van de Sande, W. W. Kallemeijn, C. L. Kuo, M. Artola, E. J. van Rooden, J. Jiang, T. J. M. Beenakker, B. I. Florea, W. A. Offen, G. J. Davies, A. J. Minnaard, J. M. Aerts, J. D. C. Codée, G. A. van der Marel and H. S. Overkleeft, Towards broad spectrum activity-based glycosidase probes: synthesis and evaluation of deoxygenated cyclophellitol aziridines, *Chem. Commun.*, 2017, **53**, 12528-12531.
24. W. W. Kallemeijn, K. Y. Li, M. D. Witte, A. R. Marques, J. Aten, S. Scheij, J. Jiang, L. I. Willems, T. M. Voorn-Brouwer, C. P. van Roomen, R. Ottenhoff, R. G. Boot, H. van den Elst, M. T. Walvoort, B. I. Florea, J. D. Codée, G. A. van der Marel, J. M. Aerts and H. S. Overkleeft, Novel activity-based probes for broad-spectrum profiling of retaining beta-exoglucosidases in situ and in vivo, *Angew. Chem. Int. Ed.*, 2012, **51**, 12529-12533.
25. K.-Y. Li, J. Jiang, M. D. Witte, W. W. Kallemeijn, H. van den Elst, C.-S. Wong, S. D. Chander, S. Hoogendoorn, T. J. M. Beenakker, J. D. C. Codée, J. M. F. G. Aerts, G. A. van der Marel and H. S. Overkleeft, Synthesis of cyclophellitol, cyclophellitol aziridine, and their tagged derivatives, *Eur. J. Org. Chem.*, 2014, **2014**, 6030-6043.
26. M. D. Witte, W. W. Kallemeijn, J. Aten, K. Y. Li, A. Strijland, W. E. Donker-Koopman, A. M. van den Nieuwendijk, B. Bleijlevens, G. Kramer, B. I. Florea, B. Hooibrink, C. E. Hollak, R. Ottenhoff, R. G. Boot, G. A. van der Marel, H. S. Overkleeft and J. M. Aerts, Ultrasensitive in situ visualization of active glucocerebrosidase molecules, *Nat. Chem. Biol.*, 2010, **6**, 907-913.
27. T. Wennekes, R. J. B. H. N. van den Berg, W. Donker, G. A. van der Marel, A. Strijland, J. M. F. G. Aerts and H. S. Overkleeft, Development of adamantan-1-yl-methoxy-functionalized 1-

deoxynojirimycin derivatives as selective inhibitors of glucosylceramide metabolism in man, *J. Org. Chem.*, 2007, **72**, 1088-1097.

28. S. P. Schröder, J. W. van de Sande, W. W. Kallemeijn, C.-L. Kuo, M. Artola, E. J. van Rooden, J. Jiang, T. J. M. Beenakker, B. I. Florea, W. A. Offen, G. J. Davies, A. J. Minnaard, J. M. F. G. Aerts, J. D. C. Codée, G. A. van der Marel and H. S. Overkleeft, Towards broad spectrum activity-based glycosidase probes: synthesis and evaluation of deoxygenated cyclophellitol aziridines, *Chem. Commun.*, 2017, **53**, 12528-12531.
29. M. Verdoes, U. Hillaert, B. I. Florea, M. Sae-Heng, M. D. P. Risseeuw, D. V. Filippov, G. A. van der Marel and H. S. Overkleeft, Acetylene functionalized BODIPY dyes and their application in the synthesis of activity based proteasome probes, *Bioorganic Med. Chem. Lett.*, 2007, **17**, 6169-6171.

Appendix - raw gels

β-D-*Araf* ABPs *in vitro* labelling

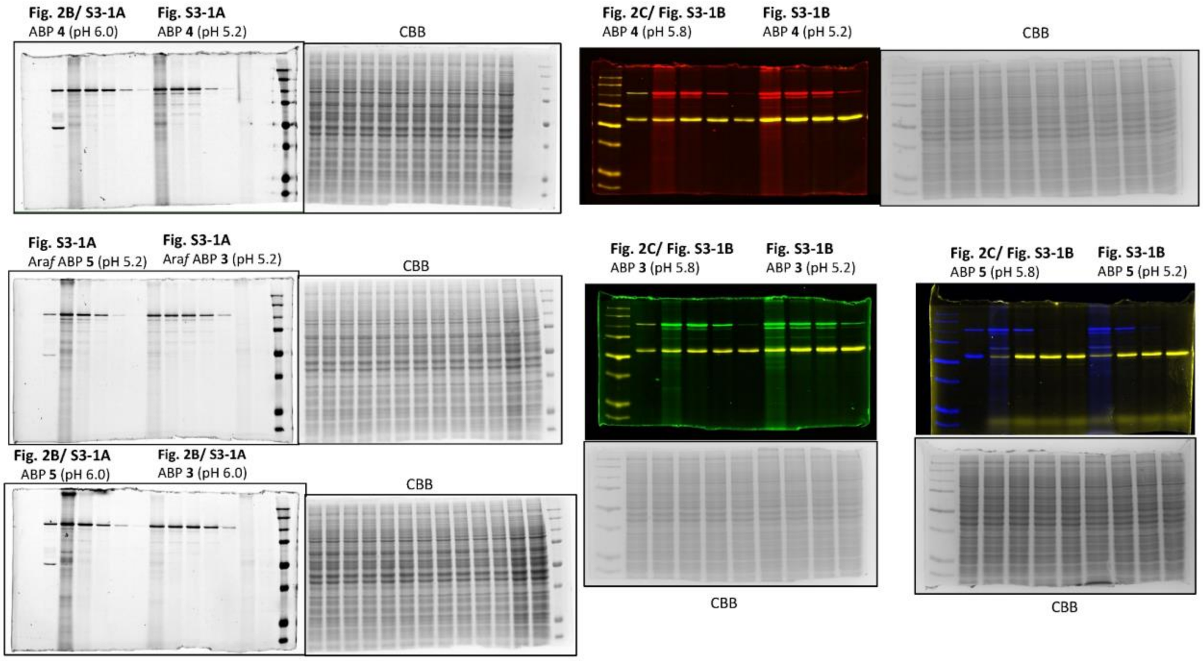

β-D-*Araf* ABPs *in vitro* labelling

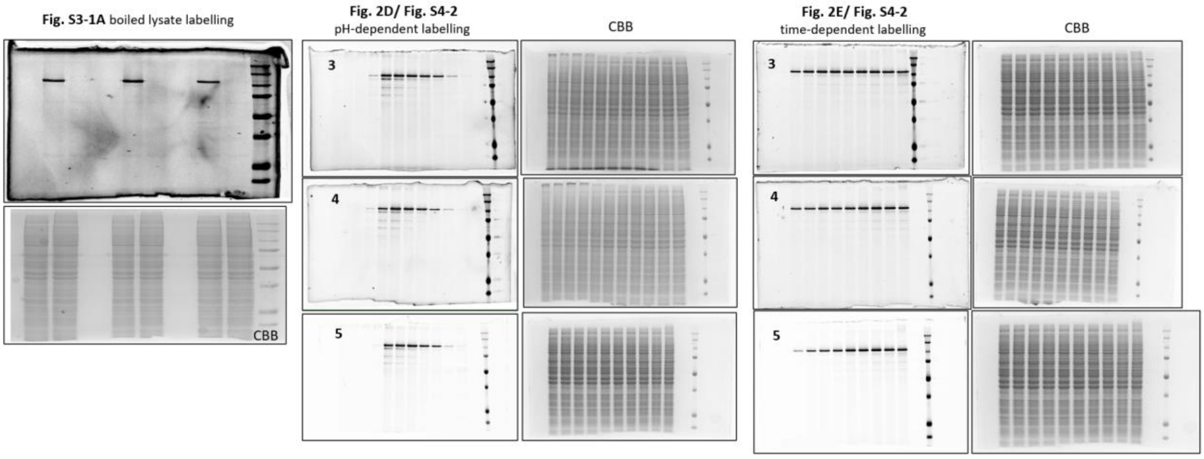

Reactivity of β-D-*Araf* ABPs *in vitro* towards GBA2 mutants

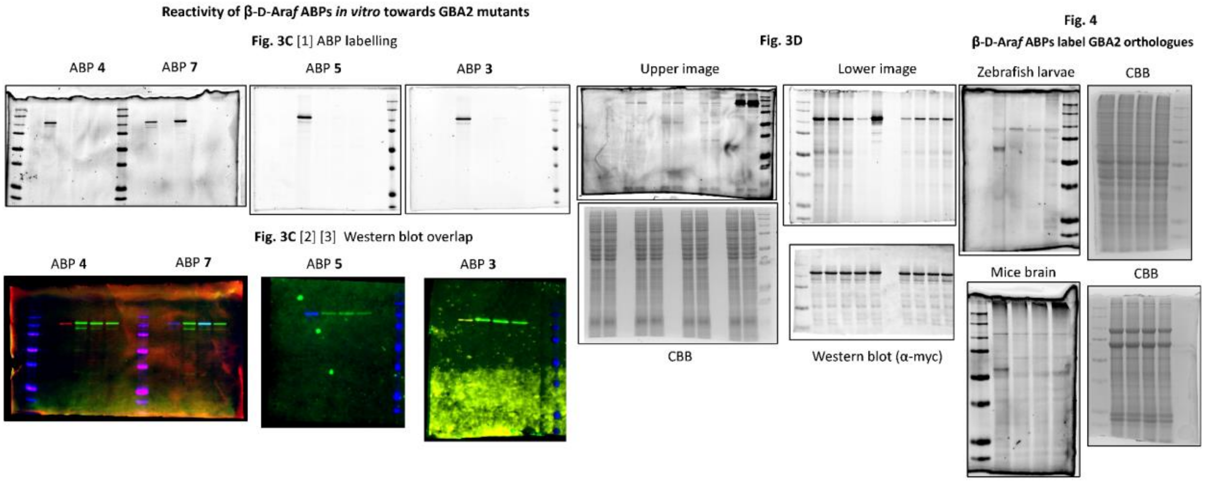

Fig. S4-1

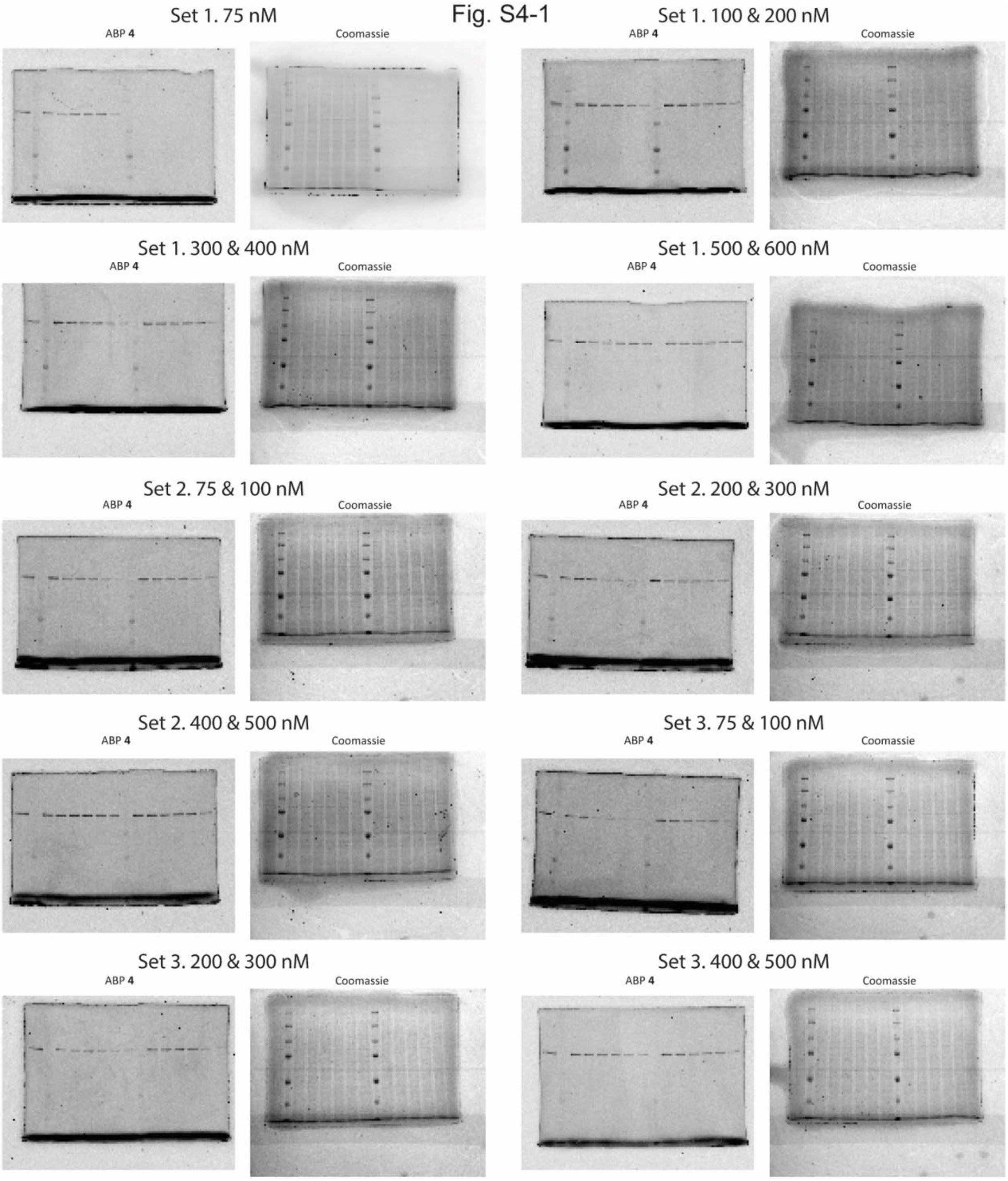
Set 1. 75 nM
ABP 4
Coomassie
Set 1. 100 & 200 nM
ABP 4
Coomassie
Set 1. 300 & 400 nM
ABP 4
Coomassie
Set 1. 500 & 600 nM
ABP 4
Coomassie
Set 2. 75 & 100 nM
ABP 4
Coomassie
Set 2. 200 & 300 nM
ABP 4
Coomassie
Set 2. 400 & 500 nM
ABP 4
Coomassie
Set 3. 75 & 100 nM
ABP 4
Coomassie
Set 3. 200 & 300 nM
ABP 4
Coomassie
Set 3. 400 & 500 nM
ABP 4
Coomassie

β-D-Ara*f* ABPs *in situ* labelling

Fig. 5A/ Fig. S7

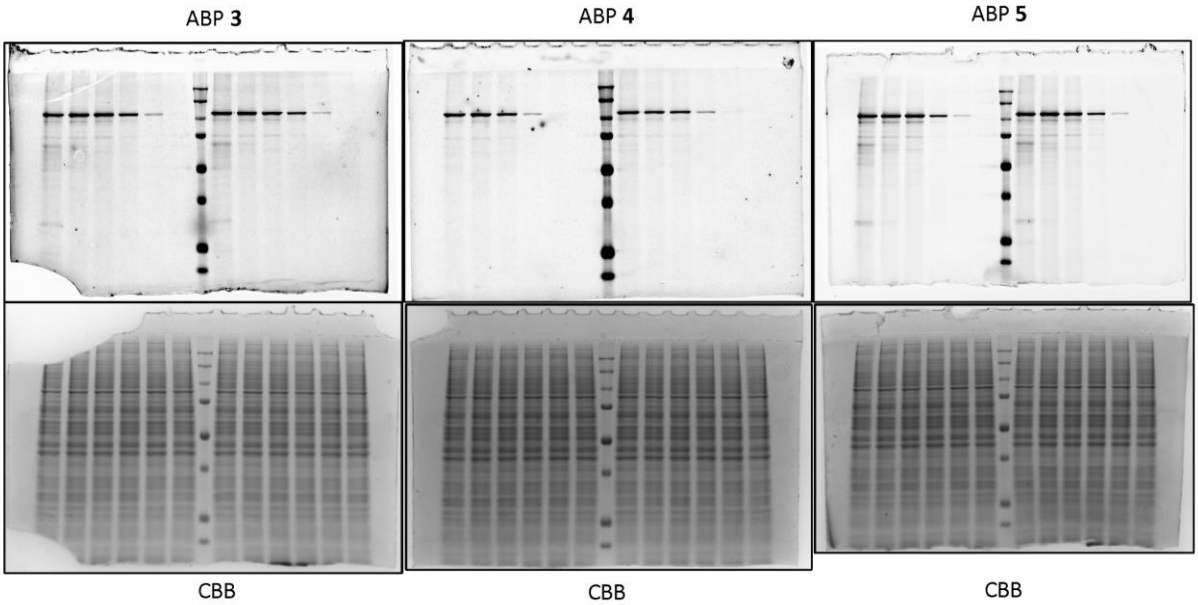

β-D-Ara*f* ABPs *in situ* labelling

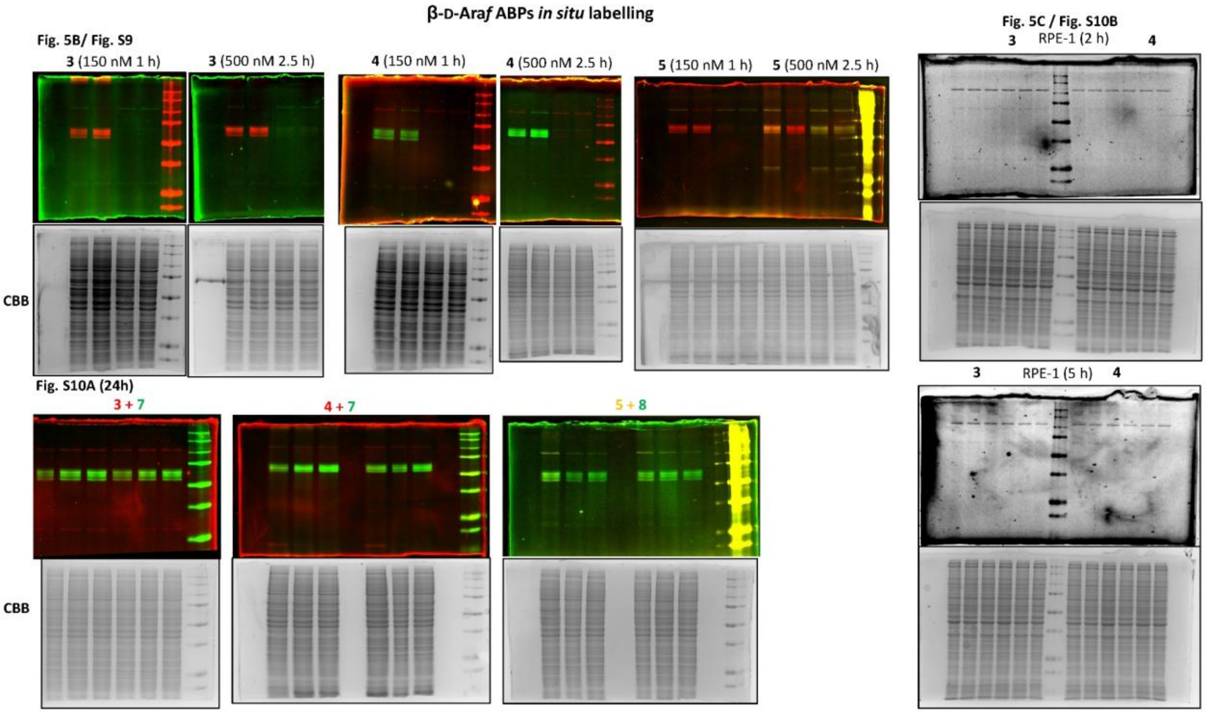

Fig. S5

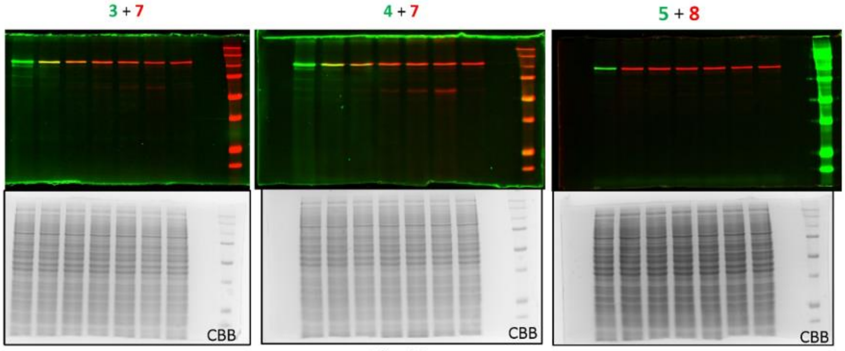

Fig. S8A

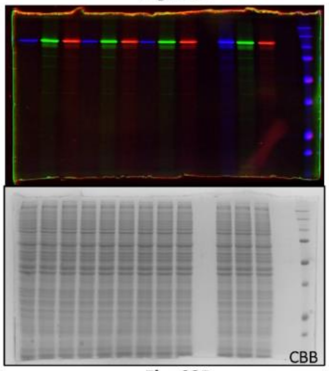

Fig. S6

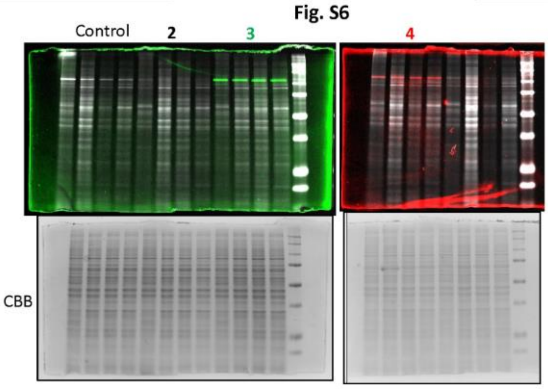

Fig. S8B

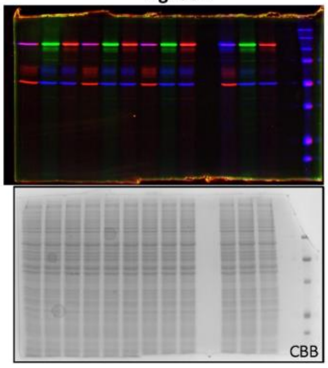

Fig. S3-2 α-L-Ara*f* ABPs *in vitro* labelling

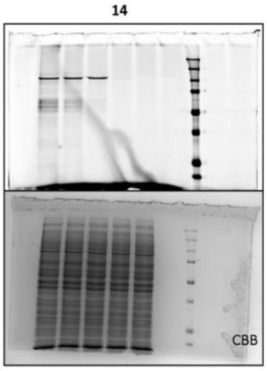

Fig. S7 α-L-Ara*f* ABPs *in situ* labelling

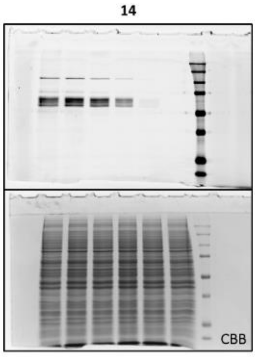

Fig. S3-2 β-L-Ara*f* ABPs *in vitro* labelling

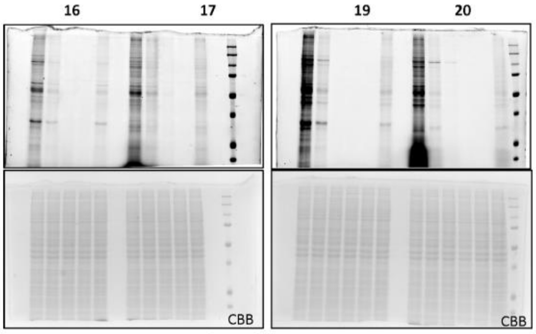

Fig. S7 β-L-Ara*f* ABPs *in situ* labelling

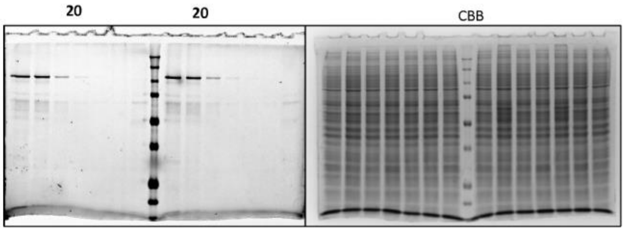

Fig. S12

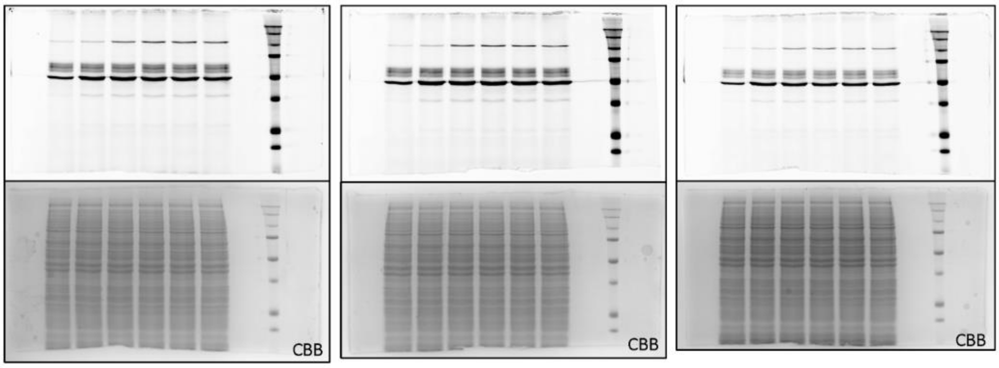

Supplement: SC-015-D3SC06146A-s001 [file SC-015-D3SC06146A-s001.pdf]
